# Assembly of Covalent Organic Frameworks into Colloidal Photonic Crystals

**DOI:** 10.1021/jacs.3c06265

**Published:** 2023-09-06

**Authors:** Javier Fonseca, Lingxin Meng, Pedro Moronta, Inhar Imaz, Cefe López, Daniel Maspoch

**Affiliations:** † Catalan Institute of Nanoscience and Nanotechnology (ICN2), CSIC, and Barcelona Institute of Science and Technology, Campus UAB, 08193 Bellaterra, Barcelona, Spain; ‡ Departament de Química, Facultat de Ciències, Universitat Autònoma de Barcelona, 08193 Bellaterra, Spain; § Instituto de Ciencia de Materiales de Madrid (ICMM), Consejo Superior de Investigaciones Científicas (CSIC), Calle Sor Juana Inés de la Cruz 3, 28049 Madrid, Spain; ∥ ICREA, Pg. Lluís Companys 23, 08010 Barcelona, Spain

## Abstract

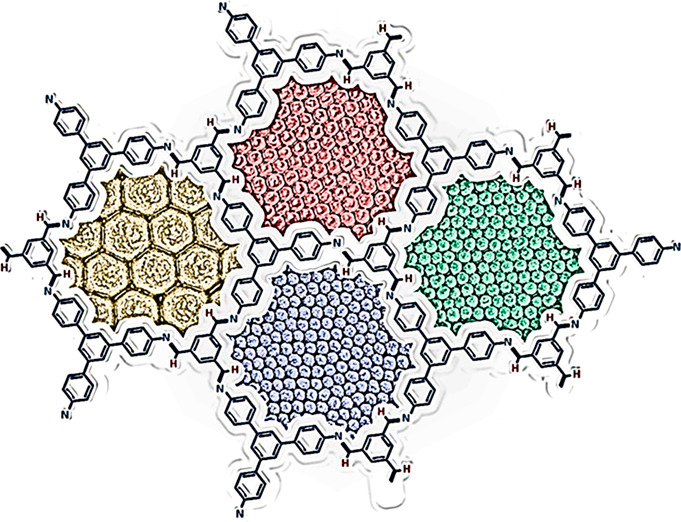

Self-assembly of colloidal particles into ordered superstructures is an important strategy to discover new materials, such as catalysts, plasmonic sensing materials, storage systems, and photonic crystals (PhCs). Here we show that porous covalent organic frameworks (COFs) can be used as colloidal building particles to fabricate porous PhCs with an underlying face-centered cubic (*fcc*) arrangement. We demonstrate that the Bragg reflection of these can be tuned by controlling the size of the COF particles and that species can be adsorbed within the pores of the COF particles, which in turn alters the Bragg reflection. Given the vast number of existing COFs, with their rich properties and broad modularity, we expect that our discovery will enable the development of colloidal PhCs with unprecedented functionality.

Colloidal photonic crystals (PhCs) or opals are periodically structured materials composed of assembled particles that can control the propagation and emission of photons.^1^ These self-assembled materials exhibit excellent optical properties, making them promising for applications such as photonics, optics and optoelectronics, sensing, solar photovoltaics, energy storage, biomedical engineering, environmental remediation, communications, and even quantum computing.^2^

Colloidal PhCs were first formed in the 1990s by self-assembly of spherical polystyrene colloidal particles.^3^ Almost simultaneously, silica particles, which are naturally forming opals, were also postulated as building particles for assembling colloidal PhCs.^4^ Significantly, the use of silica evidenced the possibility to impart to PhCs new functionalities (e.g., mesoporosity) coming from the colloidal building particles. However, to further extend the functions of PhCs, there is a need to discover and apply new functional materials as colloidal building particles into PhCs. In the past few years, more sophisticated polymeric (e.g., hydrogel) spheres, including functionalized polymeric particles and composite, Janus, or core–shell polymeric particles, have been incorporated into PhCs, thus opening the application of these crystals to displays, barcodes and sensors.^5,6^ In the field of porous PhCs, our group has introduced porous metal–organic frameworks (MOFs) as a new type of colloidal polyhedral particles to form porous PhCs.^7,8^ To date, however, the range of available porous colloidal particles for developing porous PhCs remains rather limited. Alternatively, researchers have explored other strategies to construct porous PhCs, such as etching of nonporous colloidal polymeric particles.^9^

Herein we introduce a new type of colloidal porous particles to assemble porous PhCs: purely organic crystalline covalent organic frameworks (COFs). COFs are a widely known class of porous crystalline materials that exhibit high surface areas and tunable pore sizes and compositions, and that have found a broad variety of applications, including catalysis, water harvesting, separation, and contaminant removal.^10,11^ However, unlike MOFs, they are difficult to obtain as single-crystalline particles with homogeneous sizes and shapes, which would be crucial for their self-assembly into ordered superstructures. To circumvent this barrier, we first reflected on earlier observations that COFs could be synthesized in the form of submicrometer spherical particles comprising aggregated nanocrystallites.^12^ We reasoned that by optimizing the synthesis of these spherical COF particles in terms of size monodispersity and colloidal stability, we could provoke self-assembly of COF particles into three-dimensional superstructures via depletion interactions. We further envisioned that precise control of the particle size would enable fabrication of colloidal PhCs with tunable photonic bandgaps. Here we report the results of experiments performed to prove these hypotheses.

We began by synthesizing monodisperse, submicrometer-sized colloidal spherical particles of the imine-linked TAPB-BTCA-COF.^12a^ Both 1,3,5-tris(4-aminophenyl)benzene (TAPB) (0.04 mmol) and benzene-1,3,5-tricarbaldehyde (BTCA) (0.04 mmol) were first dissolved in 5 mL of acetonitrile containing acetic acid (AcOH) (12 M). This solution was then stirred vigorously for 10 s and left undisturbed for 72 h at room temperature, resulting in the formation of a yellow-brown colloid (Figures 1a and S1). With this synthetic protocol, the size of TAPB-BTCA-COF particles composing this colloid was systematically tuned by modification of the amount of AcOH (12 M) from 1.2 to 0.2 mL. Field-emission scanning electron microscopy (FE-SEM) (Figure 1c) and dynamic light scattering (DLS) analysis (Figure S2) of the resulting colloids revealed the formation of monodisperse spherical TAPB-BTCA-COF particles with diameters of 179 ± 6 nm (1.2 mL of AcOH), 203 ± 3 nm (1.0 mL of AcOH), 220 ± 4 nm (0.8 mL of AcOH), 277 ± 5 nm (0.6 mL of AcOH), 416 ± 7 nm (0.4 mL of AcOH), and 785 ± 12 nm (0.2 mL of AcOH) (Figure 1c,d and Table S1). Powder X-ray diffraction (PXRD) of all synthesized TAPB-BTCA-COF particles indicated that they are crystalline, with patterns matching those reported for the AA-eclipsed stacking structure (Figure 1b). Nitrogen sorption measurements on these particles proved their microporosity, showing Brunauer–Emmett–Teller surface areas (SA_BET_) up to 1238 m^2^ g^–1^ (Figures S4–S21 and Table S1).^13^

**Figure 1:**
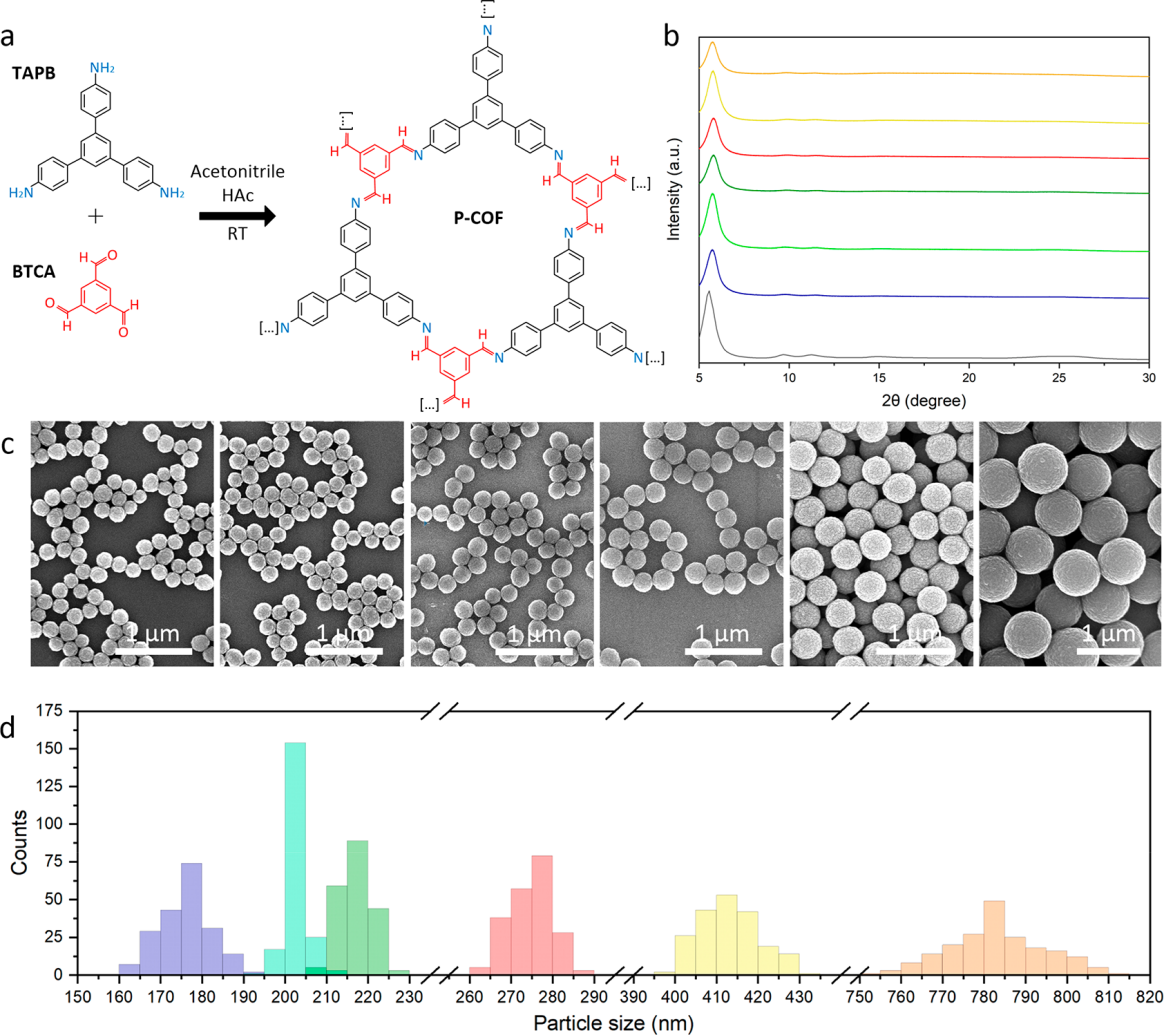
(a) Schematic of the formation of the imine-linked TAPB-BTCA-COF. (b) PXRD patterns, (c) FE-SEM images, and (d) size distribution histograms of spherical TAPB-BTCA-COF particles of different diameters. From bottom to top in (b) and left to right in (c): black, simulated; violet, 179 ± 6 nm; sky blue, 203 ± 3 nm; green, 220 ± 4 nm; red, 277 ± 5 nm; yellow, 416 ± 7 nm; orange, 785 ± 12 nm.

Next, we explored the self-assembly of TAPB-BTCA-COF particles into ordered three-dimensional superstructures on a solvophobic surface using an evaporation-induced self-assembly method. To this end, Sylgard 184 (polydimethylsiloxane, PDMS) was initially spread onto the surface of clean glass slides and subsequently cured at 120 °C to form a solvophobic surface on which the self-assembly reactions were to occur. Then a droplet of one TAPB-BTCA-COF colloid was placed onto this solvophobic surface and dried at 120 °C for 6 min, resulting in the formation of a colored film. FE-SEM images of the evaporated films revealed the formation of large, three-dimensional, ordered superstructures (Figure 2). As for the majority of spherical colloidal particles (e.g., silica, polystyrene, and acrylates),^14^ the TAPB-BTCA-COF particles self-assemble into the entropically favored face-centered cubic (*fcc*) lattice. The formation of this lattice was clearly illustrated by the characteristic top- and cross-sectional views of triangular arrangements of the spheres, which correspond to the (111) and (111̅) planes of an *fcc* arrangement, respectively (Figure 2b). FE-SEM also showed that the arrangement of these spheres reaches lengths of up to 300 μm without any cracks and with only minimal point defects, stacking faults, and dislocations (Figure 2). Note that the evaporation conditions are critical to controlling the order of the self-assembled superstructures. For instance, we found that the degree of three-dimensional ordering of TAPB-BTCA-COF particles decreased when the solvent was evaporated off at temperatures lower than or higher than 120 °C (Figures S24–S28). Similarly, we confirmed that other self-assembly approaches, including heat-assisted vertical deposition and centrifugation, could also yield COF-superstructures, albeit with a lower degree of ordering than that obtained with the solvent-evaporation method at 120 °C (Figures S29 and S30).

**Figure 2:**
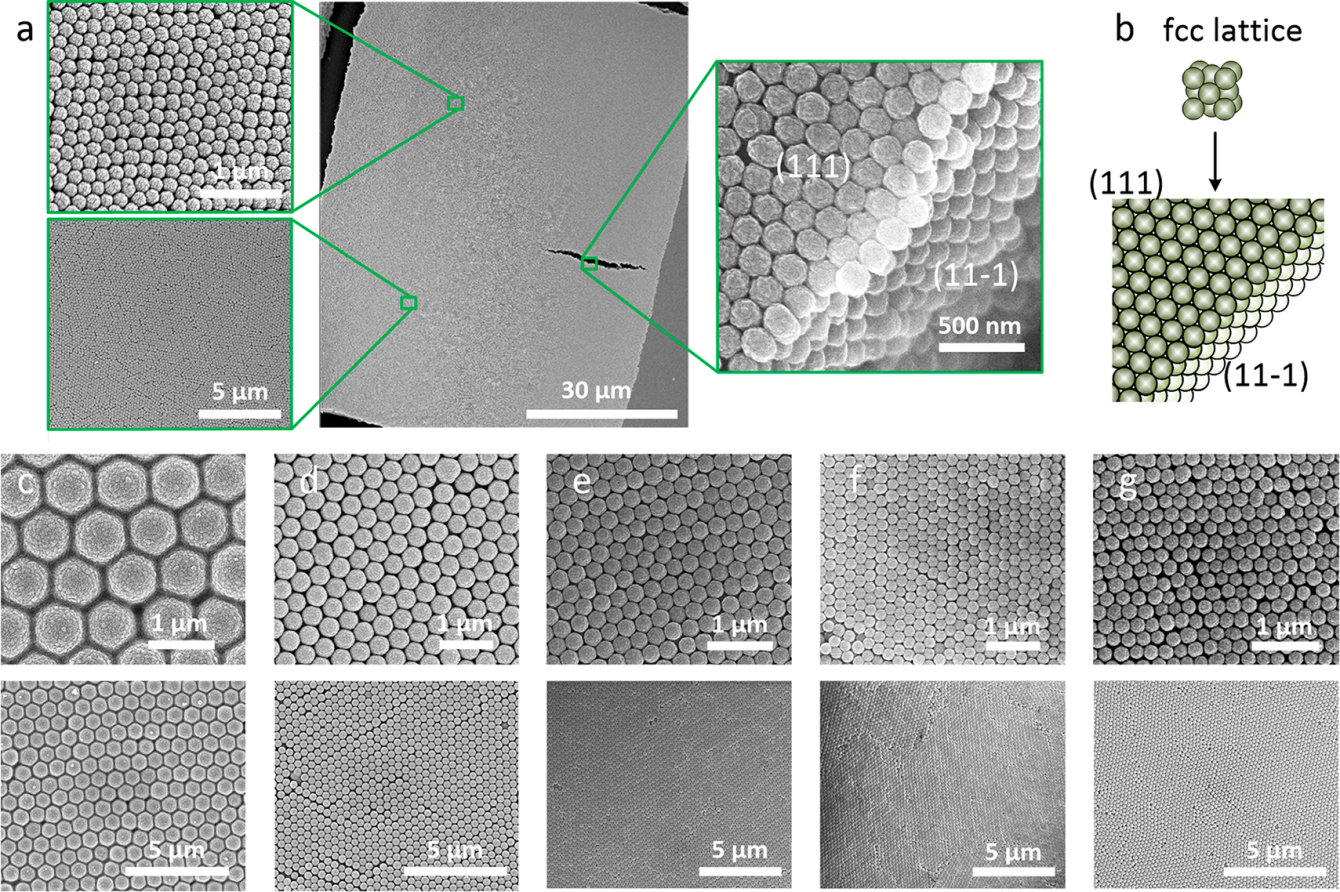
(a) FE-SEM images of a self-assembled superstructure comprising TAPB-BTCA-COF particles (179 ± 6 nm). The two magnified sections (left) show that order is maintained throughout the superstructure. The magnified section (right) is the edge of the superstructure, revealing the order in all three dimensions. (111) and (111̅) surfaces can be seen. (b) Simulation of the formation of an *fcc* lattice. (c–g) FE-SEM images of superstructures comprising TAPB-BTCA-COF particles of (c) 785 ± 12 nm, (d) 416 ± 7 nm, (e) 277 ± 5 nm, (f) 220 ± 4 nm, and (g) 203 ± 3 nm.

Another crucial factor that we identified is that the presence of unreacted linkers in the colloid is essential for colloidal stability and therefore for self-assembly of the TAPB-BTCA-COF particles. The presence of unreacted linkers was confirmed by ^1^H NMR analysis of the supernatant (Figure S31) once the TAPB-BTCA-COF particles had been separated out from the colloid by centrifugation. Clear evidence of the importance of the unreacted linkers was found when such linkers were removed from the colloids by consecutive centrifugation/washing steps. Under these new conditions, the resulting TAPB-BTCA-COF particles exhibited a lower colloidal stability, which precluded their ability to form ordered assembled structures. Thus, unreacted linkers are cardinal for stabilizing TAPB-BTCA-COF colloids and for their subsequent self-assembly into ordered structures. We attributed this to the notion that the linkers also act as depletants to induce attraction among the TAPB-BTCA-COF particles.

Another remarkable feature of the various self-assembled COF particle films that we observed is their structural colors, ranging from violet to red depending on their constituent particle size, as typically seen upon formation of colloidal PhCs (Figure 3a). To optically characterize the PhCs comprising TAPB-BTCA-COF spheres with different diameters, the optical reflections were recorded using reflectance spectrophotometry at normal incidence (θ = 0°), as shown in Figures 3b, S33 and S34. The Bragg reflection or photonic bandgaps caused by the (111) crystallographic planes of the *fcc* lattices were evidenced by a clear band in each spectrum. As expected, these Bragg reflection spectral positions shifted linearly toward shorter wavelengths as the diameter of TAPB-BTCA-COF particles decreased (Table S2).

**Figure 3:**
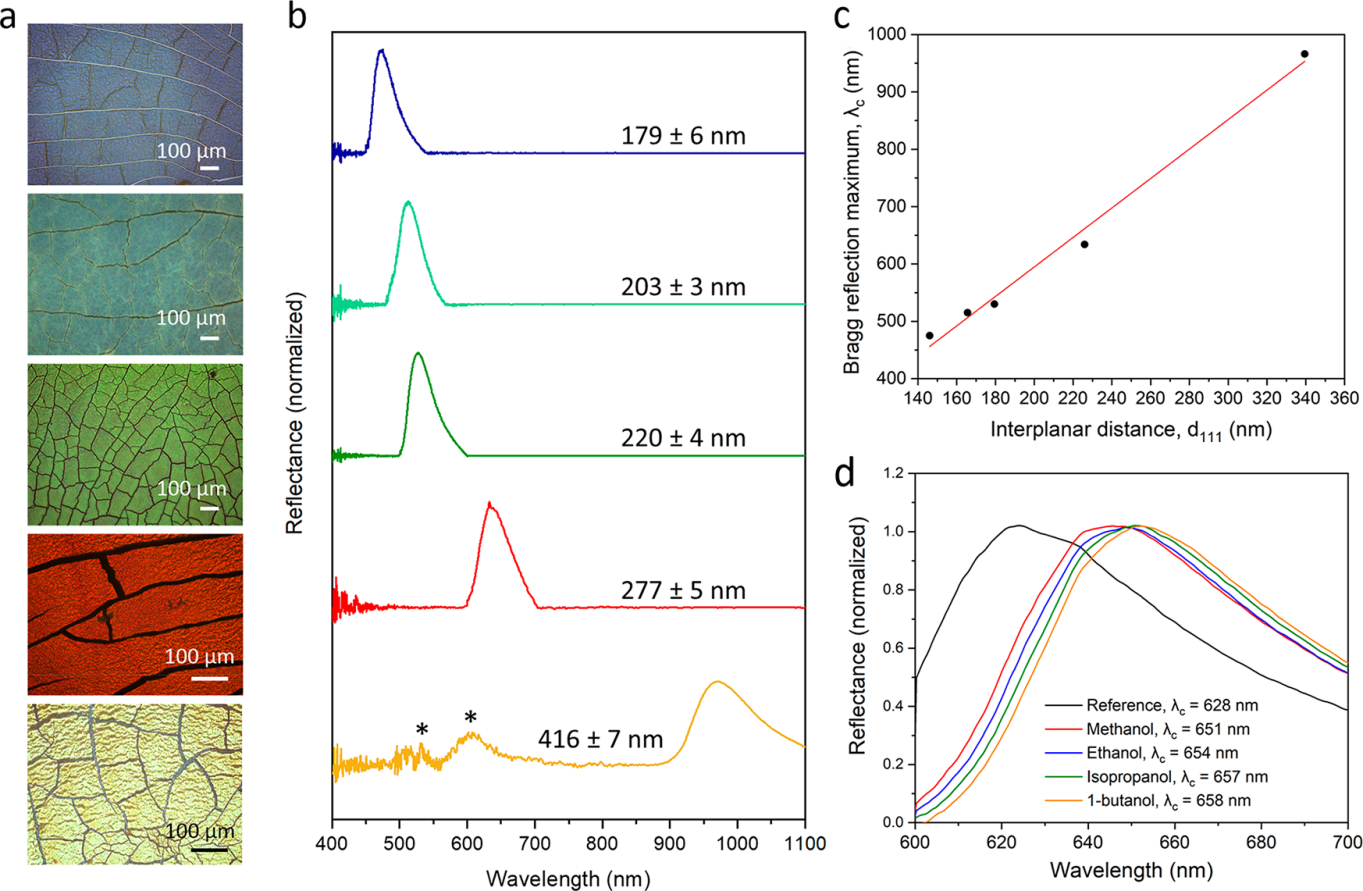
(a) Optical images and (b) optical reflectance spectra at normal incidence (θ = 0°) of the PhCs comprising TAPB-BTCA-COF particles of different sizes. The peaks marked with an asterisk correspond to high-energy photon bands. (c) Bragg reflection maximum (λ_c_) plotted against the interplanar distance (*d*_111_) and fitted to the Bragg–Snell law. (d) Optical reflectance spectra at normal incidence (θ = 0°) for the PhC comprising TAPB-BTCA-COF particles (277 ± 5 nm) as-made and after exposure to alcohol vapors.

Figure 3c shows the Bragg reflection maxima or bandgap centers (λ_c_) plotted against the interplanar distance (*d*_111_). For an *fcc* lattice, the interplanar spacing for (111) planes is given by *d*_111_ = 0.816·*D*, where *D* is the diameter of the TAPB-BTCA-COF particles. We fitted λ_c_ using the Bragg–Snell law for normal incidence: λ_c_ = 2·*n*·*d*_111_, where *n* is the effective index of refraction of the PhC. From the slope of the fitted curve, it is possible to calculate the effective refractive index (*n*_eff_) to be 1.46. Next, we calculated the refractive index of COF particles by averaging the effective dielectric function: ϵ_eff_ = *n*_eff_^2^ = *n*_c_^2^·*V*_c_ + *n*_m_^2^·*V*_m_, where *n*_c_ and *n*_m_ are the refractive indices of the COF particles and surrounding medium, respectively, and *V*_c_ and *V*_m_ are their corresponding volume fractions. For (111) planes in the *fcc* lattice, the volume fractions of COF particles and the surrounding medium are approximately 0.74 and 0.26, respectively.^15^ Since the surrounding medium was air and *n*_air_ is 1.00,^16^ the refractive index (*n*_c_) of COF particles was determined to be 1.59, which is close to the values found in polymeric materials.^17^

Moreover, colloidal PhCs comprising COF particles with diameters larger than 277 ± 5 nm (specifically, 416 ± 7 or 785 ± 12 nm) exhibited optical reflectance in the spectral range corresponding to higher-energy photonic bands, usually referred to as second-order diffraction (Figures 3b and S33). In this range, a more complex photonic band structure describes the states accessible to photons.^18,19^ The reflectance spectra exhibited by these PhCs exhibit a set of spectral fluctuations that do not always correspond to the presence of forbidden bands.

An interesting property of PhCs built from porous materials is their potential to adsorb species within their pores. We sought to test examples of this adsorption, exploring any consequential changes in the Bragg reflection maximum. To this end, we first measured the N_2_ adsorption–desorption isotherm of these PhCs, from which we calculated SA_BET_ values close to those exhibited by their TAPB-BTCA-COF building particles (up to 990 m^2^ g^−1^; Figures S36–S53 and Table S1). Having confirmed the microporous character of the photonic structures, we next exposed an evacuated PhC comprising TAPB-BTCA-COF particles (diameter: 277 ± 5 nm) to vapors of one of various alcohols, including methanol, ethanol, isopropanol, and 1-butanol. In each case, we found a red shift in λ_c_ when the PhC was exposed to the alcohol vapors (methanol, λ_c_ = 651 nm; ethanol, λ_c_ = 654 nm; isopropanol, λ_c_ = 657 nm; 1-butanol, λ_c_ = 658 nm; Figure 3d and Table S3). Significantly, after the alcohol molecules had been evacuated from the COF pores by heating at 120 °C for 30 min, λ_c_ shifted back to the initial value of the original PhC (S54).

Having demonstrated the self-assembly of spherical TAPB-BTCA-COF particles into colloidal PhCs, we extended the formation of COF-based PhCs to a second imine-linked COF, hereafter termed TAPB-TP-COF (where TP is terephthalaldehyde; Figure 4a). Using a synthetic protocol similar to that used for TAPB-BTCA-COF, a colloid composed of monodisperse urchin-like-shaped TAPB-TP-COF particles with a diameter of 282 ± 14 nm was synthesized (Figures S55–S59, S61–S63 and Table S4). Afterward, we self-assembled these COF particles on a solvophobic surface using the evaporation-induced method. Despite not exhibiting a perfect spherical shape, FE-SEM of the resulting red film revealed that these urchin-like particles can also self-assemble into an *fcc* arrangement, forming a colloidal PhC exhibiting a photonic bandgap at λ_c_ = 664 nm together with a high-energy photonic band feature (Figures 4b–d and S65–S67).

**Figure 4:**
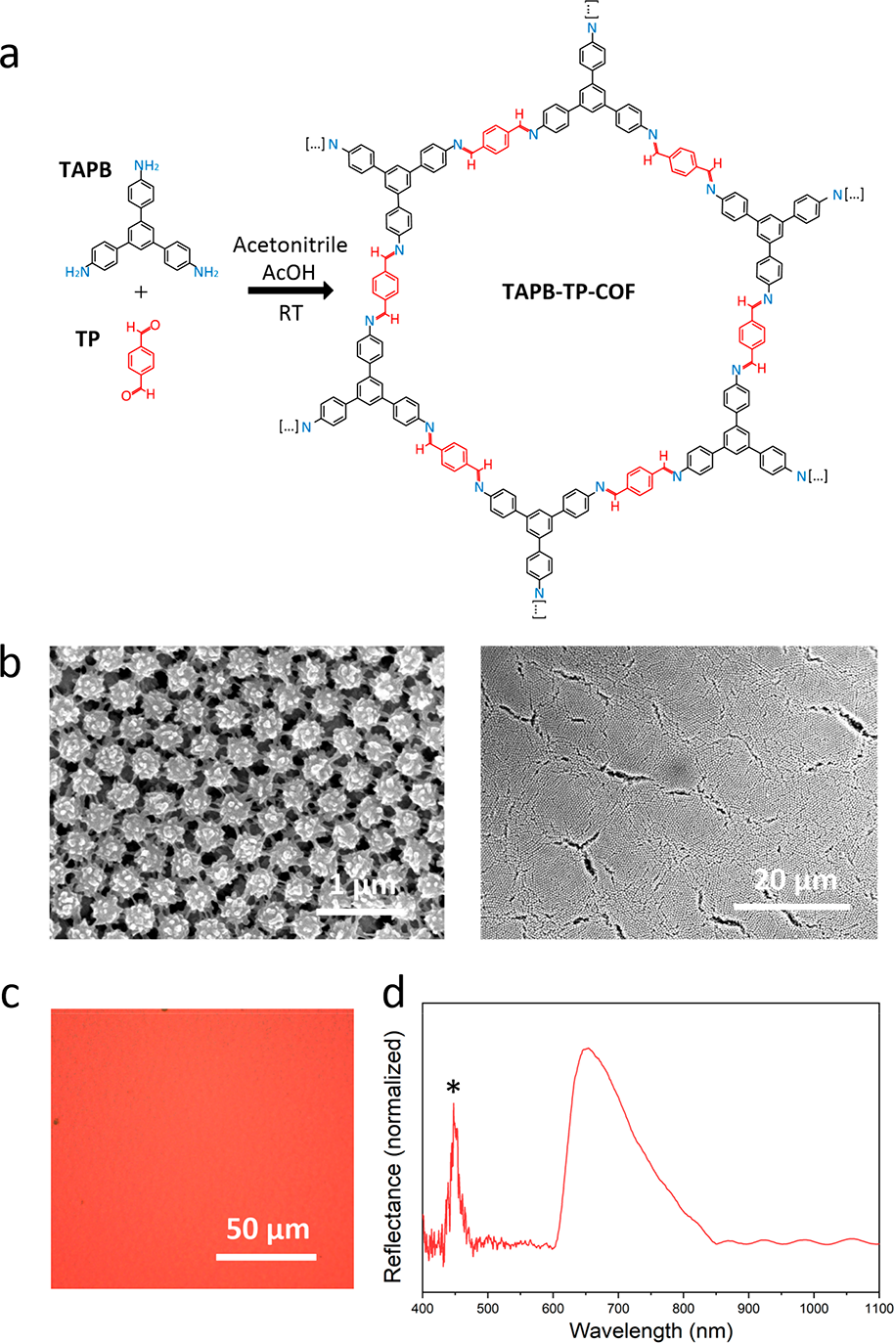
(a) Schematic of the formation of TAPB-TP-COF. (b) FE-SEM images of a superstructure comprising urchin-like-shaped TAPB-TP-COF particles. (c) Optical image and (d) optical reflectance spectrum at normal incidence (θ = 0°) of the PhC comprising TAPB-TP-COF particles.

In conclusion, we have reported the first-ever example of the self-assembly of monodisperse COF particles into porous colloidal PhCs. The resulting PhCs exhibit an *fcc* lattice and corresponding photonic bandgap that can be tuned either by controlling the size of the COF particles or by changing the species adsorbed in the pores of the COF particles. Our findings suggest the potential applicability of COF-based PhCs as responsive materials or sensors. We believe that this first demonstration of the possibility to use COFs to create long-range-ordered superstructures opens the door to the future design of new sensing materials (e.g., through the use of COF-10 or COF-DL229 for ammonia and iodine sensing),^20^ catalysts, PhCs, and storage systems.

## Supporting Information Available

Detailed synthesis, DLS analysis, FT-IR spectra, FE-SEM data and images, nitrogen adsorption–desorption isotherms, optical images, optical reflectance spectra, TGA analysis, and PXRD diffractograms (PDF)
