# Supplementary material for: Assembly of Covalent Organic Frameworks into Colloidal Photonic Crystals
Source: J Am Chem Soc. 2023 Sep 6;145(37):20163–8. doi: 10.1021/jacs.3c06265 (PMC10515629; doi:10.1021/jacs.3c06265)
Supplement: Supplementary file 1 — ja3c06265_si_001.pdf [file ja3c06265_si_001.pdf]

Supporting Information

Assembly of Covalent Organic Frameworks into Colloidal Photonic Crystals

Javier Fonseca,[ab] Lingxin Meng,[ab] Pedro Moronta,[c] Inhar Imaz,[*ab] Cefe López,[*c] and Daniel Maspoch[*abd]

a Catalan Institute of Nanoscience and Nanotechnology (ICN2), CSIC and The Barcelona Institute of Science and Technology, Campus UAB, Bellaterra, Barcelona, Spain
b Departament de Química, Facultat de Ciències, Universitat Autònoma de Barcelona, 08193 Bellaterra, Spain
c Instituto de Ciencia de Materiales de Madrid (ICMM), Consejo Superior de Investigaciones Científicas (CSIC), Calle Sor Juana Inés de la Cruz 3, E-28049 Madrid, Spain
d ICREA, Pg. Lluıs Companys 23, Barcelona, Spain

Email: inhar.imaz@icn2.cat; c.lopez@csic.es; daniel.maspoch@icn2.cat

Table of Content

1. **Materials and methods**
   1.1. Chemicals
   1.2. Characterization methods

2. **Material synthesis**
   2.1. Synthesis of colloidal spherical TAPB-BTCA-COF particles with different sizes
   2.2. Synthesis of colloidal urchin-like shaped TAPB-TP-COF particles
   2.3. Treatment of colloidal COF particles for their subsequent characterization as individual particles (no as self-assembled superstructures)
   2.4. Self-assembly of colloidal covalent organic framework particles into photonic crystals
   2.5. Guest sorption measurements

3. **Supplementary figures**

Figure S1. Photograph of a colloidal solution of TAPB-BTCA-COF particles.
Figure S2. Dynamic light scattering histogram of the size distribution of spherical TAPB-BTCA-COF particles with different diameters: (a) mean average size: 258. 7 nm (PdI: 0.009) (measured from FE-SEM images: 179 ± 6 nm); (b) mean average size: 259. 5 nm (PdI: 0.057) (measured from FE-SEM images: 203 ± 3 nm); (c) mean average size: 279. 4 nm (PdI: 0.027) (measured from FE-SEM images: 220 ± 4 nm); (d) mean average size: 343 nm (PdI: 0.073) (measured from FE-SEM images: 277 ± 5 nm); (e) mean average size: 443.4 nm (PdI: 0.124) (measured from FE-SEM images: 416 ± 7 nm); and (f) mean average size: 1141.0 nm (PdI: 0.197) (measured from FE-SEM images: 785 ± 12 nm).
Figure S3. FT-IR spectra of spherical of TAPB (black) and BTCA (dark blue) organic linkers, and TAPB-BTCA-COF particles (from up to bottom: orange, 785 ± 12 nm; yellow, 416 ± 7 nm; red, 277 ± 5 nm; green, 220 ± 4; sky blue, 203 ± 3 nm; and violet, 179 ± 6 nm). Note that these spectra show the presence of the typical imine (C=N) stretching band of at ~ 1624 cm^{-1}, indicative of the successful condensation reaction between the amino groups of TAPB and the aldehyde groups of BTCA.
Figure S4. Nitrogen adsorption-desorption isotherm of TAPB-BTCA-COF (179 ± 6 nm) at 77 K.
Figure S5. BETSI analysis of TAPB-BTCA-COF (179 ± 6 nm) (S_{BET} = 1238 m^2 g^{-1}).
Figure S6. BETSI regression diagnostics for TAPB-BTCA-COF (179 ± 6 nm) (S_{BET} = 1238 m^2 g^{-1}).
Figure S7. Nitrogen adsorption-desorption isotherm of TAPB-BTCA-COF (203 ± 3 nm) at 77 K.
Figure S8. BETSI analysis of TAPB-BTCA-COF (203 ± 3 nm) (S_{BET} = 1131 m^2 g^{-1}).
Figure S9. BETSI regression diagnostics for TAPB-BTCA-COF (203 ± 3 nm) (S_{BET} = 1131 m^2 g^{-1}).
Figure S10. Nitrogen adsorption-desorption isotherm of TAPB-BTCA-COF (220 ± 4 nm) at 77 K.
Figure S11. BETSI analysis of TAPB-BTCA-COF (220 ± 4 nm) (S_{BET} = 964 m^2 g^{-1}).
Figure S12. BETSI regression diagnostics for TAPB-BTCA-COF (220 ± 4 nm) (S_{BET} = 964 m^2 g^{-1}).
Figure S13. Nitrogen adsorption-desorption isotherm of TAPB-BTCA-COF (277 ± 5 nm) at 77 K.
Figure S14. BETSI analysis of TAPB-BTCA-COF (277 ± 5 nm) (S_{BET} = 534 m^2 g^{-1}).
Figure S15. BETSI regression diagnostics for TAPB-BTCA-COF (277 ± 5 nm) (S_{BET} = 534 m^2 g^{-1}).

Figure S16. Nitrogen adsorption-desorption isotherm of TAPB-BTCA-COF (416 ± 7 nm) at 77 K.
Figure S17. BETSI analysis of TAPB-BTCA-COF (416 ± 7 nm) (S_{BET} = 399 m^2 g^{-1}).
Figure S18. BETSI regression diagnostics for TAPB-BTCA-COF (416 ± 7 nm) (S_{BET} = 399 m^2 g^{-1}).
Figure S19. Nitrogen adsorption-desorption isotherm of TAPB-BTCA-COF (785 ± 12 nm) at 77 K.
Figure S20. BETSI analysis of TAPB-BTCA-COF (785 ± 12 nm) (S_{BET} = 223 m^2 g^{-1}).
Figure S21. BETSI regression diagnostics for TAPB-BTCA-COF (785 ± 12 nm) (S_{BET} = 223 m^2 g^{-1}).
Figure S22. TGA analysis of PhCs made of TAPB-BTCA-COF particles. The weight loss around 500 °C is attributed to the decomposition of the COF particles.
Figure S23. FE-SEM images of a PhC made of TAPB-BTCA-COF particles (203 ± 3 nm) before and after isothermal treatment at 220 °C for 2 h. These images show that, after isothermal treatment, the PhC retained its initial arrangement. Inset: optical reflectance spectra at normal incidence (θ=0°) of the PhC made of TAPB-BTCA-COF particles (203 ± 3 nm) before and after isothermal treatment at 220 °C for 2 h. The optical spectra further confirm that the superstructure remains highly ordered after isothermal treatment.
Figure S24. FE-SEM images of superstructures formed by evaporation-induced self-assembly of TAPB-BTCA-COF particles of different diameters, (a) 179 ± 6 nm, (b) 203 ± 3 nm, (c) 220 ± 4 nm, (d) 277 ± 5 nm, (e) 416 ± 7 nm, and (f) 785 ± 12 nm, at 140 °C.
Figure S25. FE-SEM images of superstructures formed by evaporation-induced self-assembly of TAPB-BTCA-COF particles of different diameters, (a) 179 ± 6 nm, (b) 203 ± 3 nm, (c) 220 ± 4 nm, (d) 277 ± 5 nm, (e) 416 ± 7 nm, and (f) 785 ± 12 nm, at 100 °C.
Figure S26. FE-SEM images of superstructures formed by evaporation-induced self-assembly of TAPB-BTCA-COF particles of different diameters, (a) 179 ± 6 nm, (b) 203 ± 3 nm, (c) 220 ± 4 nm, (d) 277 ± 5 nm, (e) 416 ± 7 nm, and (f) 785 ± 12 nm, at 65 °C.
Figure S27. FE-SEM images of superstructures formed by evaporation-induced self-assembly of TAPB-BTCA-COF particles of different diameters, (a) 179 ± 6 nm, (b) 203 ± 3 nm, (c) 220 ± 4 nm, (d) 277 ± 5 nm, (e) 416 ± 7 nm, and (f) 785 ± 12 nm, at RT.
Figure S28. FE-SEM images of superstructures formed by evaporation-induced self-assembly of TAPB-BTCA-COF particles of different diameters, (a) 179 ± 6 nm, (b) 203 ± 3 nm, (c) 220 ± 4 nm, (d) 277 ± 5 nm, (e) 416 ± 7 nm, and (f) 785 ± 12 nm, at 2 °C.
Figure S29. FE-SEM images of superstructures formed by heat-assisted vertical deposition self-assembly of TAPB-BTCA-COF particles of different diameters, (a) 179 ± 6 nm, (b) 203 ± 3 nm, (c) 220 ± 4 nm, (d) 277 ± 5 nm, (e) 416 ± 7 nm, and (f) 785 ± 12 nm, at 45 °C.
Figure S30. FE-SEM images of superstructures formed by centrifugation-based self-assembly of TAPB-BTCA-COF particles of different diameters, (a) 179 ± 6 nm, (b) 203 ± 3 nm, (c) 220 ± 4 nm, (d) 277 ± 5 nm, (e) 416 ± 7 nm, and (f) 785 ± 12 nm.
Figure S31. ^{1}H NMR spectra (300 MHz, CDCl3) of TAPB (black) and the supernatant resulting from the centrifugation of TAPB-BTCA-COF particles (277 ± 5 nm) (red).
Figure S32. (a-f) Photographs of the self-assembled PhCs made of TAPB-BTCA-COF particles of different sizes: (a) 179 ± 6 nm; (b) 203 ± 3 nm; (c) 220 ± 4 nm; (d) 277 ± 5 nm; (e) 416 ± 7 nm; and (f) 785 ± 12 nm. Although the color of the COF-based PhCs is influenced by the yellow-brown color of the COF particles, the typical angle-dependent opalescence of colloidal PhCs is appreciated.
Figure S33. (a) Optical image and (b) optical reflectance spectrum at normal incidence (θ = 0°) of the self-assembled photonic crystals made of TAPB-BTCA-COF particles of diameter: 785 ± 12 nm. The peak marked with an asterisk is assumed to correspond to a high-energy photon band. The Bragg reflection maximum is expected to appear around 1600-1700 nm, which is outside the reading range of the spectrophotometer.

Figure S34. Optical reflectance at θ = 0° for the PhCs made of TAPB-BTCA-COF particles of different sizes: 179 ± 6 nm (violet); 203 ± 3 nm (sky blue); 220 ± 4 nm (green); 277 ± 5 nm (red); 416 ± 7 nm (yellow); and 785 ± 12 nm (orange).
Figure S35. Bragg reflection maximum (λ_c) plotted against the interplanar distance (d_{111}) and fitted to the Bragg-Snell law. Inset: fitting function and correlation coefficient.
Figure S36. Nitrogen adsorption-desorption isotherm of photonic crystals made of TAPB-BTCA-COF particles (179 ± 6 nm) at 77 K.
Figure S37. BETSI analysis of photonic crystals made of TAPB-BTCA-COF particles (179 ± 6 nm) (S_{BET} = 990 m^2 g^{-1}).
Figure S38. BETSI regression diagnostics for photonic crystals made of TAPB-BTCA-COF particles (179 ± 6 nm) (S_{BET} = 990 m^2 g^{-1}).
Figure S39. Nitrogen adsorption-desorption isotherm of photonic crystals made of TAPB-BTCA-COF particles (203 ± 3 nm) at 77 K.
Figure S40. BETSI analysis of photonic crystals made of TAPB-BTCA-COF particles (203 ± 3 nm) (S_{BET} = 761 m^2 g^{-1}).
Figure S41. BETSI regression diagnostics for photonic crystals made of TAPB-BTCA-COF particles (203 ± 3 nm) (S_{BET} = 761 m^2 g^{-1}).
Figure S42. Nitrogen adsorption-desorption isotherm of photonic crystals made of TAPB-BTCA-COF particles (220 ± 4 nm) at 77 K.
Figure S43. BETSI analysis of photonic crystals made of TAPB-BTCA-COF particles (220 ± 4 nm) (S_{BET} = 584 m^2 g^{-1}).
Figure S44. BETSI regression diagnostics for photonic crystals made of TAPB-BTCA-COF particles (220 ± 4 nm) (S_{BET} = 584 m^2 g^{-1}).
Figure S45. Nitrogen adsorption-desorption isotherm of photonic crystals made of TAPB-BTCA-COF particles (277 ± 5 nm) at 77 K.
Figure S46. BETSI analysis of photonic crystals made of TAPB-BTCA-COF particles (277 ± 5 nm) (S_{BET} = 514 m^2 g^{-1}).
Figure S47. BETSI regression diagnostics for photonic crystals made of TAPB-BTCA-COF particles (277 ± 5 nm) (S_{BET} = 514 m^2 g^{-1}).
Figure S48. Nitrogen adsorption-desorption isotherm of photonic crystals made of TAPB-BTCA-COF particles (416 ± 7 nm) at 77 K.
Figure S49. BETSI analysis of photonic crystals made of TAPB-BTCA-COF particles (416 ± 7 nm) (S_{BET} = 272 m^2 g^{-1}).
Figure S50. BETSI regression diagnostics for photonic crystals made of TAPB-BTCA-COF particles (416 ± 7 nm) (S_{BET} = 272 m^2 g^{-1}).
Figure S51. Nitrogen adsorption-desorption isotherm of photonic crystals made of TAPB-BTCA-COF particles (785 ± 12 nm) at 77 K.
Figure S52. BETSI analysis of photonic crystals made of TAPB-BTCA-COF particles (785 ± 12 nm) (S_{BET} = 123 m^2 g^{-1}).
Figure S53. BETSI regression diagnostics for photonic crystals made of TAPB-BTCA-COF particles (785 ± 12 nm) (S_{BET} = 123 m^2 g^{-1}).
Figure S54. Bragg reflection maxima at normal incidence (θ=0°) for the PhCs comprising TAPB-BTCA-COF particles (203 ± 3 nm) before and after exposure to ethanol as a function of the number of adsorption-desorption cycles. The optical reflectance of the PhCs was measured after 30 min of exposure to the vapours of ethanol. At the end of each cycle and before the next one, the PhCs were activated by desorbing the ethanol at 120 °C for 30 min. No significant decrease of the Bragg reflection maxima was found throughout the ethanol adsorption-desorption cycles, indicating the high stability of theses PhCs.
Figure S55. PXRD pattern of the urchin-like shaped TAPB-TP-COF particles. Black, reference; and red, urchin-like shaped TAPB-TP-COF particles.
Figure S56. FT-IR spectra of urchin-like shaped TAPB-TP-COF particles (red), and TP (blue) and TAPB (black) linkers.

Figure S57. Photograph of a colloidal solution of TAPB-TP-COF particles.
Figure S58. (a) Representative FE-SEM image of a self-assembled superstructure made of TAPB-TP-COF particles. (b) Size-distribution histogram of urchin-like shaped TAPB-TP-COF particles. Mean size = 282 nm. Standard deviation = 14.
Figure S59. Dynamic light scattering histogram of the size distribution of urchin-like shaped TAPB-TP-COF particles. Mean average size: 327.5 nm (PdI: 0.104) (measured from FE-SEM images: 282 ± 14 nm).
Figure S60. Photograph of the self-assembled PhC made of TAPB-TP-COF particles. The color of the COF-based PhC is strongly influenced by the yellow color of the COF particles.
Figure S61. Nitrogen adsorption-desorption isotherm of TAPB-TP-COF particles (282 ± 14 nm) at 77 K.
Figure S62. BETSI analysis of TAPB-TP-COF (282 ± 14 nm) (S_{BET} = 112 m^2 g^{-1}).
Figure S63. BETSI regression diagnostics for TAPB-TP-COF (282 ± 14 nm) (S_{BET} = 112 m^2 g^{-1}).
Figure S64. TGA analysis of PhCs made of TAPB-TP-COF particles. The weight loss around 500 °C is attributed to the decomposition of the COF particles.
Figure S65. Nitrogen adsorption-desorption isotherm of photonic crystals made of TAPB-TP-COF particles (282 ± 14 nm) at 77 K.
Figure S66. BETSI analysis of photonic crystals made of TAPB-TP-COF particles (282 ± 14 nm) (S_{BET} = 51 m^2 g^{-1}).
Figure S67. BETSI regression diagnostics for photonic crystals made of TAPB-TP-COF particles (282 ± 14 nm) (S_{BET} = 51 m^2 g^{-1}).

4. Supplementary tables

Supplementary Table 1. Diameter of spherical TAPB-BTCA-COF particles. BET surface area of TAPB-BTCA-COF particles and TAPB-BTCA-COF-based photonic crystals.
Supplementary Table 2. Diameter of spherical TAPB-BTCA-COF particles. Interplanar distance and optical bandgap of TAPB-BTCA-COF-based photonic crystals.
Supplementary Table 3. Bandgaps and calculated refractive indices of the self-assembled photonic crystals (particle size: 277 ± 5 nm) exposure to different alcohols.
Supplementary Table 4. Diameter of spherical TAPB-TP-COF particles. BET surface area of TAPB-TP-COF particles and TAPB-TP-COF-based photonic crystals.

5. References

1. Materials and methods

1.1. Chemicals

1,3,5-tris(4-aminophenyl)benzene (TAPB) was acquired from BLD pharma. Benzene-1,3,5-tricarboxaldehyde (BTCA) was purchased from TCI. Terephthalaldehyde (TP), 1-butanol and sylgard® 184 were obtained from Sigma-Aldrich Co. Acetonitrile, glacial acetic acid (AcOH), methanol (MeOH) and isopropanol were purchased from Fisher Chemical. Ethanol (EtOH) was acquired from LabKem. All reagents and solvents were used without further purification. Deionized water was obtained with a Milli-Q® system (18.2 MΩ·cm).

1.2. Characterization methods

Powder X-ray diffraction (PXRD) measurements were performed on a Panalytical X'pert diffractometer with monochromatic Cu-Ka radiation (λ_{Cu} = 1.5406 Å). *Field-emission scanning electron microscopy (FE-SEM)* images were collected on a scanning electron microscope (FEI Magellan 400L XHR). *The size distributions* of COF particles were estimated statistically from FE-SEM images by measuring the diameter of 200 particles in different areas of a sample. *Particle size distributions* were also assessed by dynamic light scattering using a Malvern Zetasizer, (Malvern Instruments, UK). *Optical microscopy* images were obtained on a Nikon Eclipse LV100 microscope. *Optical reflectance* was measured using a Fourier-transform infrared spectrometer, IFS 66S from Bruker, coupled to an IR microscope. *Fourier transform infrared (FT-IR)* spectra were recorded on a Bruker Tensor 27 FTIR spectrometer equipped with a Golden Gate diamond attenuated total reflection (ATR) cell, in transmittance mode at room temperature. *Volumetric N_2 sorption* measurements were collected at 77 K using an ASAP 2020 HD gas adsorption system (Micromeritics). BET specific surface area was determined by using the software BETSI.[1] *Proton Nuclear Magnetic Resonance (^{1}H NMR)* spectra were recorded on Bruker Avance 300 MHz spectrometer. Thermogravimetric analyses (TGA) were performed in a Pyris TGA8000 from 30 °C to 700 °C with a heating rate of 5 °C min^{-1} under N_2 atmosphere.

2. Material synthesis

2.1. Synthesis of colloidal spherical TAPB-BTCA-COF particles with different sizes

Colloidal spherical TAPB-BTCA-COF particles with different sizes were synthesized according to the previously reported method with slight modifications.[2] TAPB (14.058 mg, 0.04 mmol) and BTCA (6.486 mg, 0.04 mmol) were added into 13.5 mL vials. These reagents were completely dissolved in acetonitrile (5 mL) by sonication for 1 min. Subsequently, 0.2, 0.4, 0.6, 0.8, 1.0 or 1.2 mL (12 M) of AcOH were poured into the solution. Finally, the mixture was stirred vigorously for 10 s and left undisturbed at room temperature for 72 h. In doing so, final colloidal solutions with concentrations up to 1 mg mL^{-1} were obtained. It should be mentioned that, by decreasing the amount of acetic acid from 1.2, 1.0, 0.8, 0.6 and 0.4 to 0.2 mL, the diameter of the spherical TAPB-BTCA-COF particles in the colloidal solutions was found to increase from 179 ± 6 nm, 203 ± 3 nm, 220 ± 4 nm, 277 ± 5 nm and 416 ± 7 nm to 785 ± 12 nm, respectively. Therefore, the key point to control the size of the COFs is to adjust the amount of catalyst, acetic acid. Specifically, acetic acid favours nucleation in the synthesis of these COFs, so that, as nucleation points increase, the amount of reagent available to grow the COF particles decreases, resulting in small-sized COF particles.

2.2. Synthesis of colloidal urchin-like shaped TAPB-TP-COF particles

Colloidal urchin-like shaped TAPB-TP-COF particles were synthesized according to the previously reported method with slight modifications.[2] TAPB (14.058 mg, 0.04 mmol) and TP (8.048 mg, 0.06 mmol) were added into 13.5 mL vials. These reagents were completely dissolved in acetonitrile (5 mL) by sonication for 1 min. Subsequently, 1 mL (12 M) of AcOH was poured into the solution. Finally, the mixture was stirred vigorously for 10 s and left undisturbed at room temperature for 72 h. In doing so, a final colloidal solution with a concentration up to 1.5 mg mL^{-1} was obtained.

2.3. Treatment of colloidal COF particles for their subsequent characterization as individual particles (not as self-assembled superstructures)

COF particles were precipitated by centrifugation at 9000 rpm and washed with dry THF and EtOH for three times. Then, the powders were dried under vacuum for 24 h.

2.4. Self-assembly of colloidal covalent organic framework particles into photonic crystals

Sylgard® 184 was initially spread on the surface of clean glass microscope slides and subsequently cured at 120 °C for 30 min. Then, 100 µL of each as-prepared colloidal solution of COF particles were dropped onto this solvophobic substrate and dried in an oven at 120 °C for 6 min.

2.5. Guest sorption measurements

We designed a custom vapour chamber to *in-situ* measure the optical reflectance of the photonic crystals made of TAPB-BTCA-COF particles after exposure to MeOH, EtOH, isopropanol and 1-butanol. The photonic crystals were placed inside two concentric Petri dishes (one closing the other and, therefore, simulating a chamber), slightly elevated with respect to the "floor" Petri dish thanks to a support. The alcohol of interest was poured into the chamber up to the height of the photonic crystals but without touching them. The optical reflectance of the photonic crystals was measured with the spectrophotometer after

30 min of exposure to the vapours of each respective alcohol. At the end of each optical reflectance measurement and before the next one, the photonic crystals were activated by desorbing the previously adsorbed vapour. Desorption was performed by heating the photonic crystals at 120 °C for 30 min until the bandgap shifted back to its initial position of 634 nm.

3. Supplementary figures

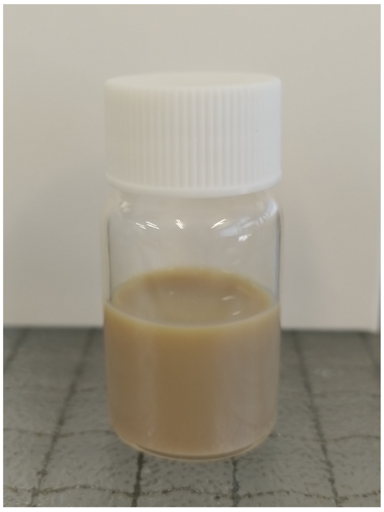

Figure S1. Photograph of a colloidal solution of TAPB-BTCA-COF particles.

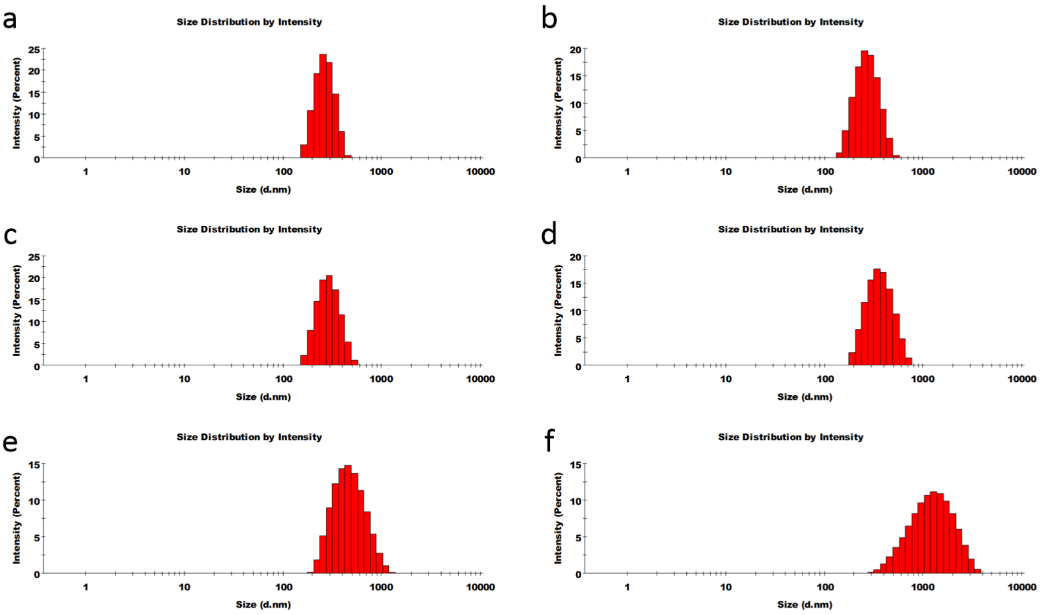

Figure S2. Dynamic light scattering histogram of the size distribution of spherical TAPB-BTCA-COF particles with different diameters: (a) mean average size: 258. 7 nm (PdI: 0.009) (measured from FE-SEM images: 179 ± 6 nm); (b) mean average size: 259. 5 nm (PdI: 0.057) (measured from FE-SEM images: 203 ± 3 nm); (c) mean average size: 279. 4 nm (PdI: 0.027) (measured from FE-SEM images: 220 ± 4 nm); (d) mean average size: 343 nm (PdI: 0.073) (measured from FE-SEM images: 277 ± 5 nm); (e) mean average size: 443.4 nm (PdI: 0.124) (measured from FE-SEM images: 416 ± 7 nm); and (f) mean average size: 1141.0 nm (PdI: 0.197) (measured from FE-SEM images: 785 ± 12 nm).

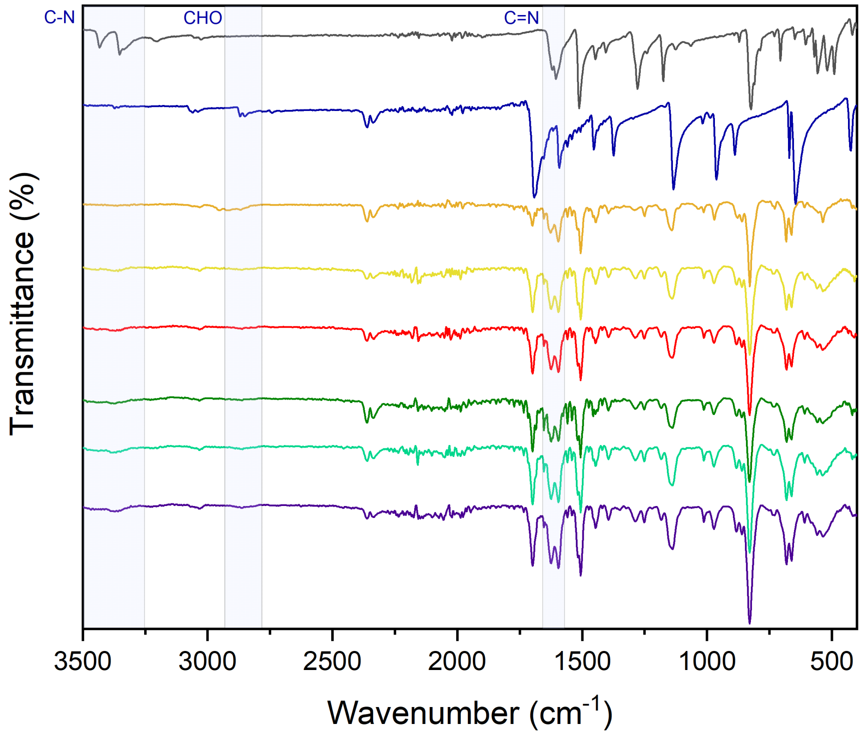

Figure S3. FT-IR spectra of spherical of TAPB (black) and BTCA (dark blue) organic linkers, and TAPB-BTCA-COF particles (from up to bottom: orange, 785 ± 12 nm; yellow, 416 ± 7 nm; red, 277 ± 5 nm; green, 220 ± 4; sky blue, 203 ± 3 nm; and violet, 179 ± 6 nm). Note that these spectra show the presence of the typical imine (C=N) stretching band of at ~ 1624 cm^{-1}, indicative of the successful condensation reaction between the amino groups of TAPB and the aldehyde groups of BTCA.

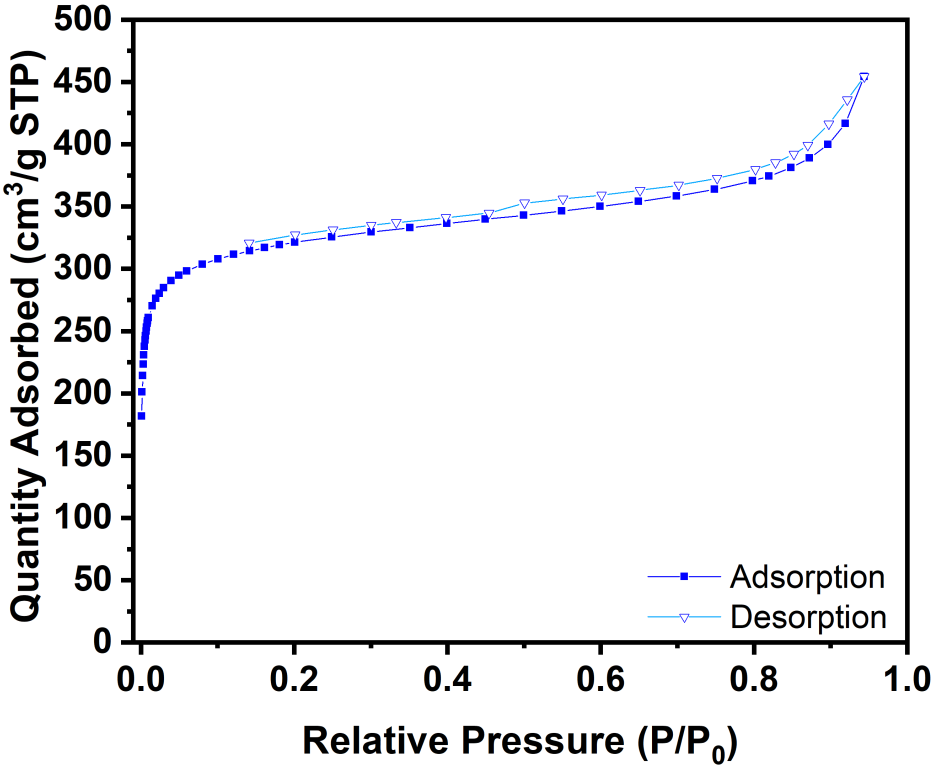

Figure S4. Nitrogen adsorption-desorption isotherm of TAPB-BTCA-COF (179 ± 6 nm) at 77 K.

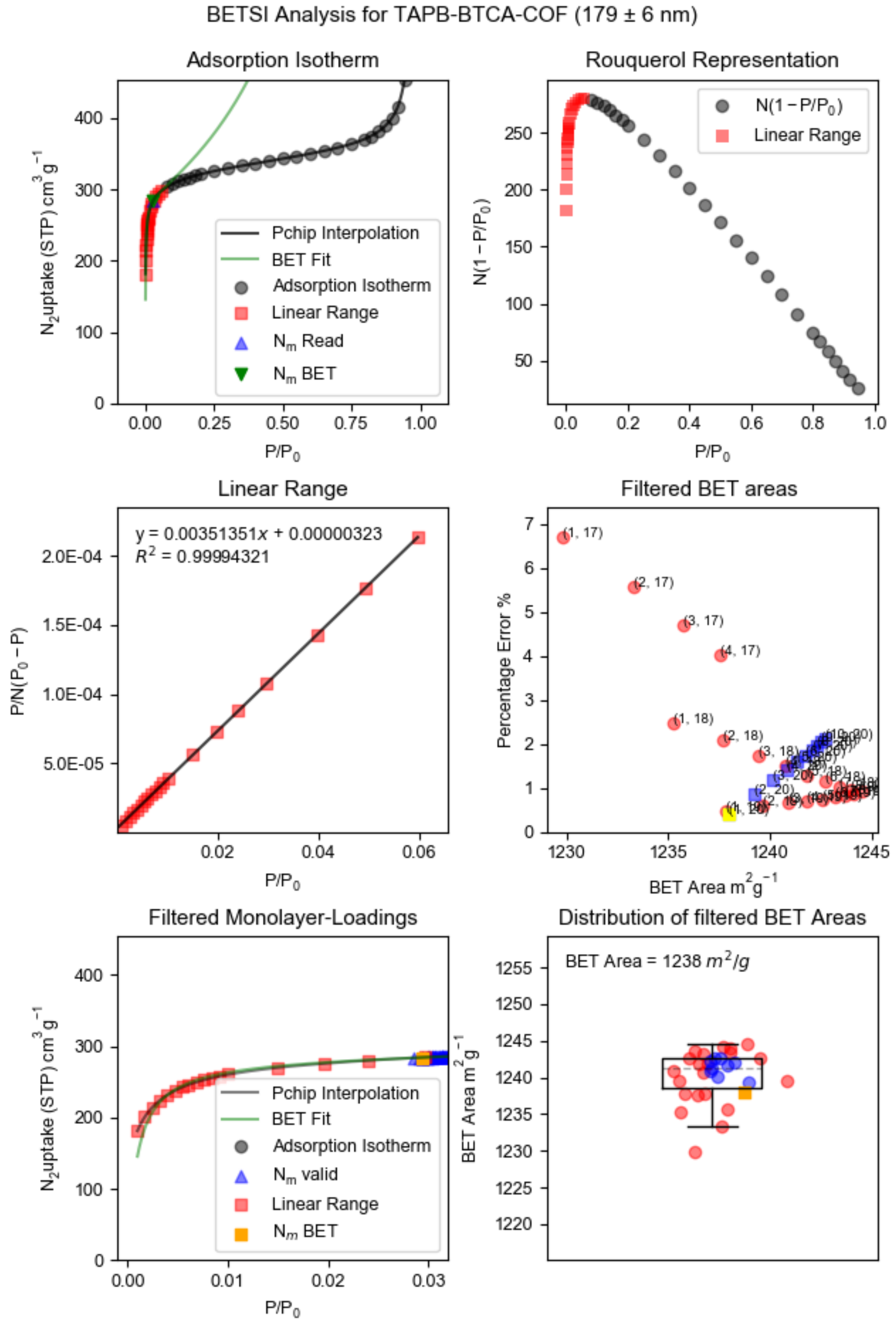

Figure S5. BETSI analysis of TAPB-BTCA-COF (179 ± 6 nm) (S_{BET} = 1238 m² g⁻¹).

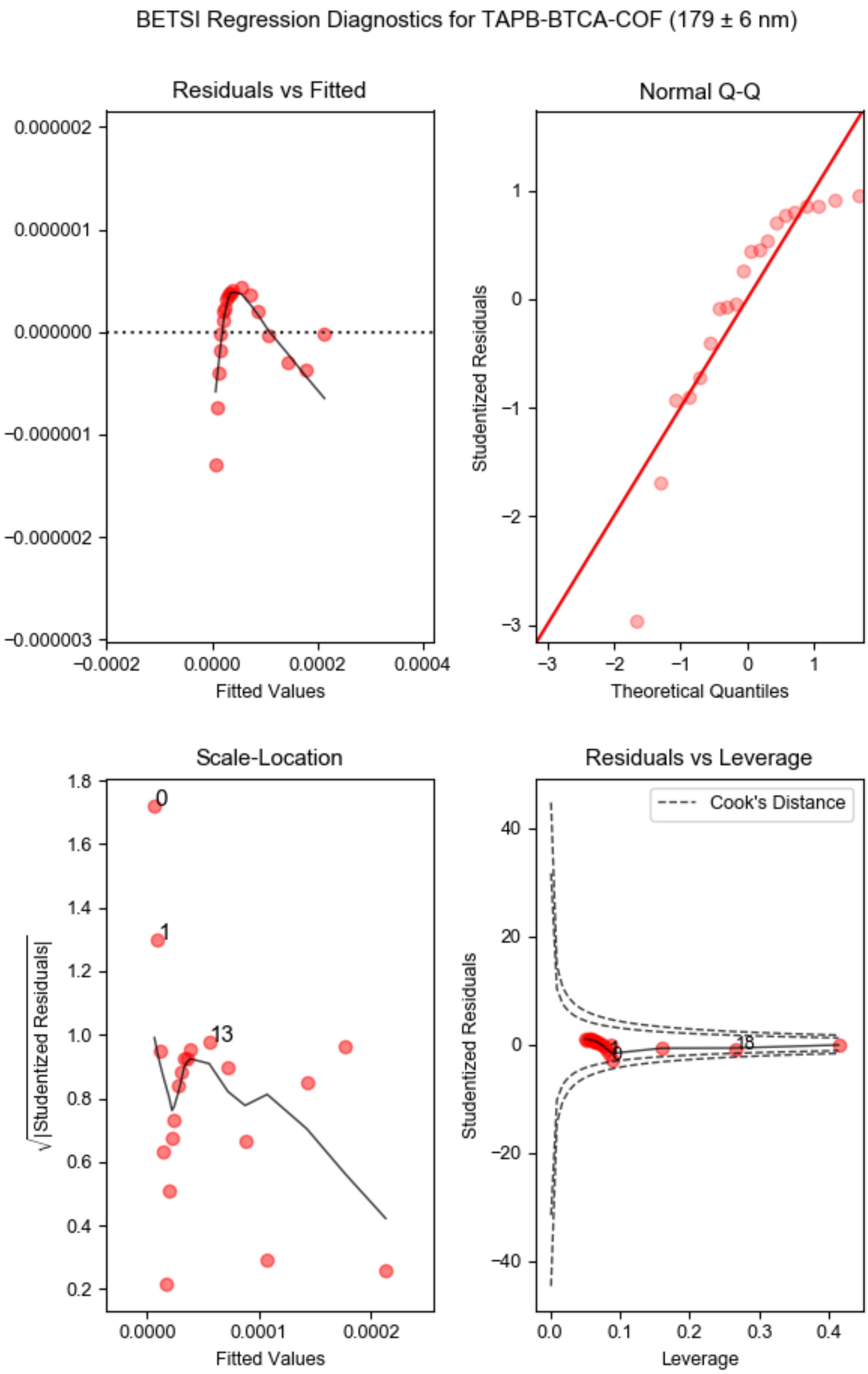

Figure S6. BETSI regression diagnostics for TAPB-BTCA-COF (179 ± 6 nm) (S_{BET} = 1238 m^2 g^{-1}).

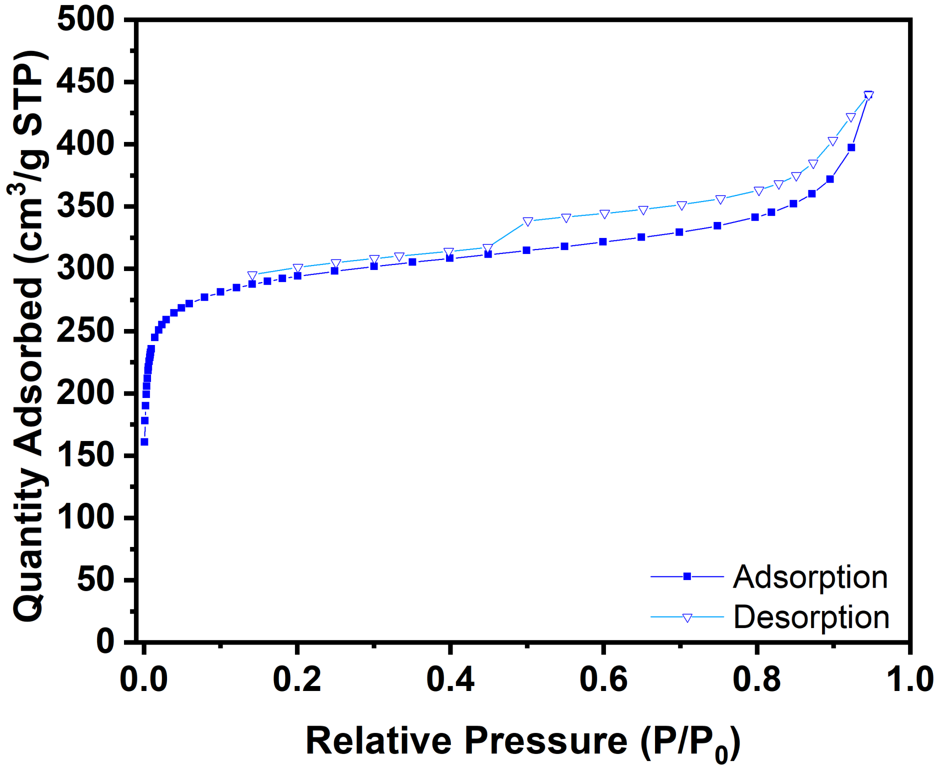

Figure S7. Nitrogen adsorption-desorption isotherm of TAPB-BTCA-COF (203 ± 3 nm) at 77 K.

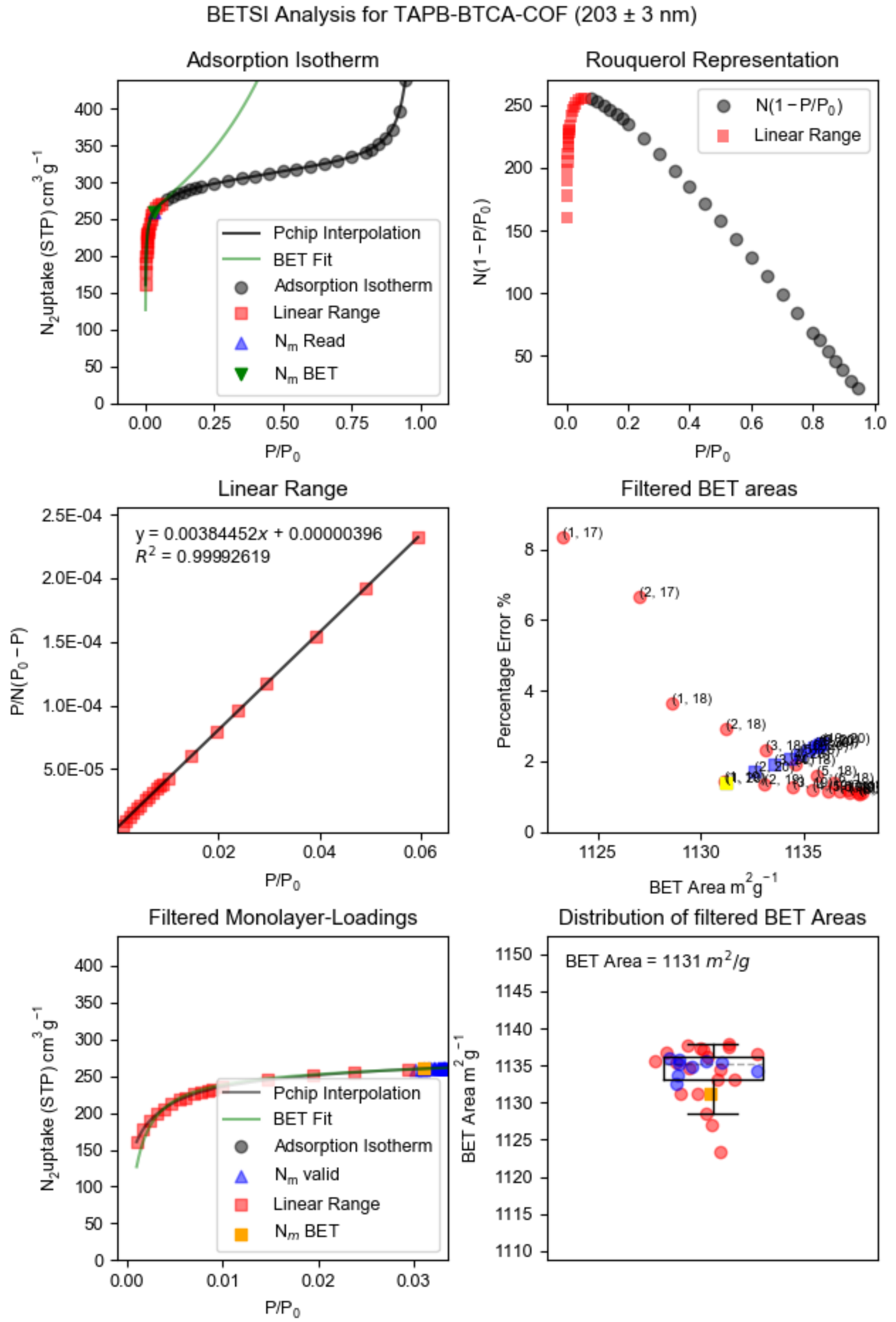

Figure S8. BETSI analysis of TAPB-BTCA-COF (203 ± 3 nm) (S_{BET} = 1131 m^2 g^{-1}).

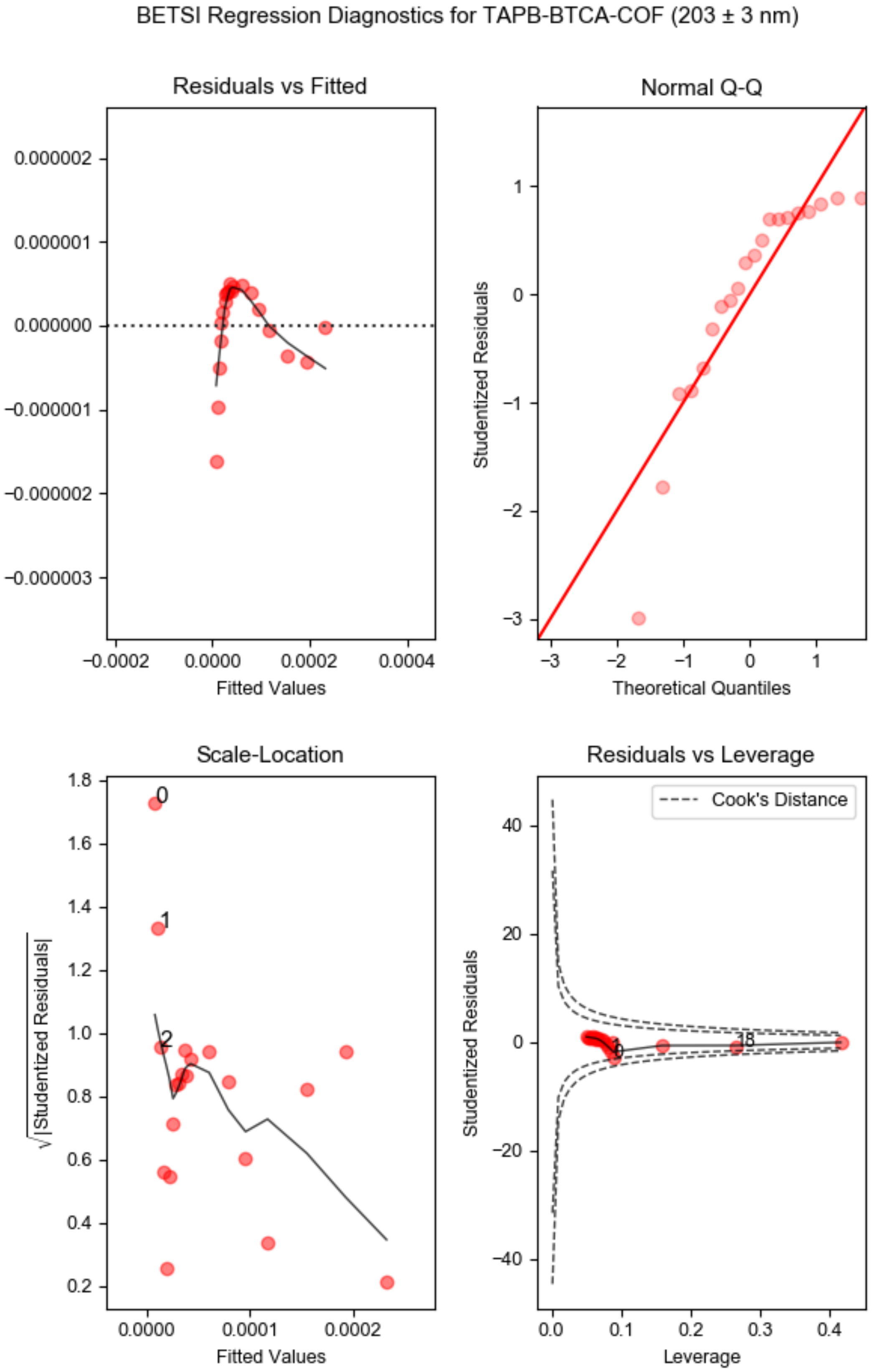

Figure S9. BETSI regression diagnostics for TAPB-BTCA-COF (203 ± 3 nm) (S_{BET} = 1131 m^2 g^{-1}).

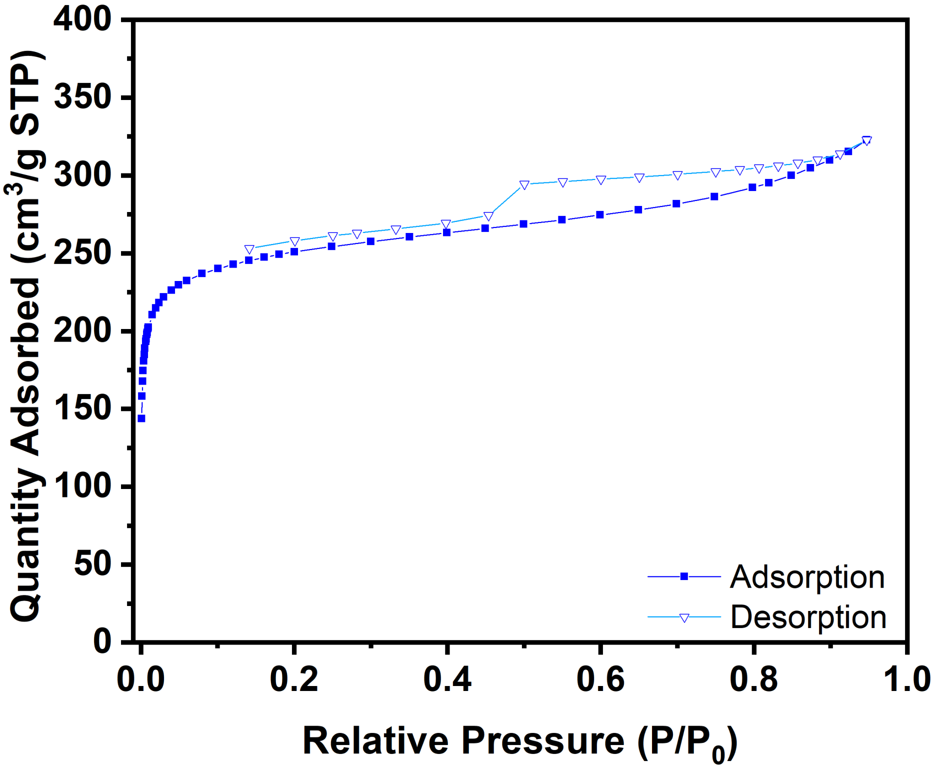

Figure S10. Nitrogen adsorption-desorption isotherm of TAPB-BTCA-COF (220 ± 4 nm) at 77 K.

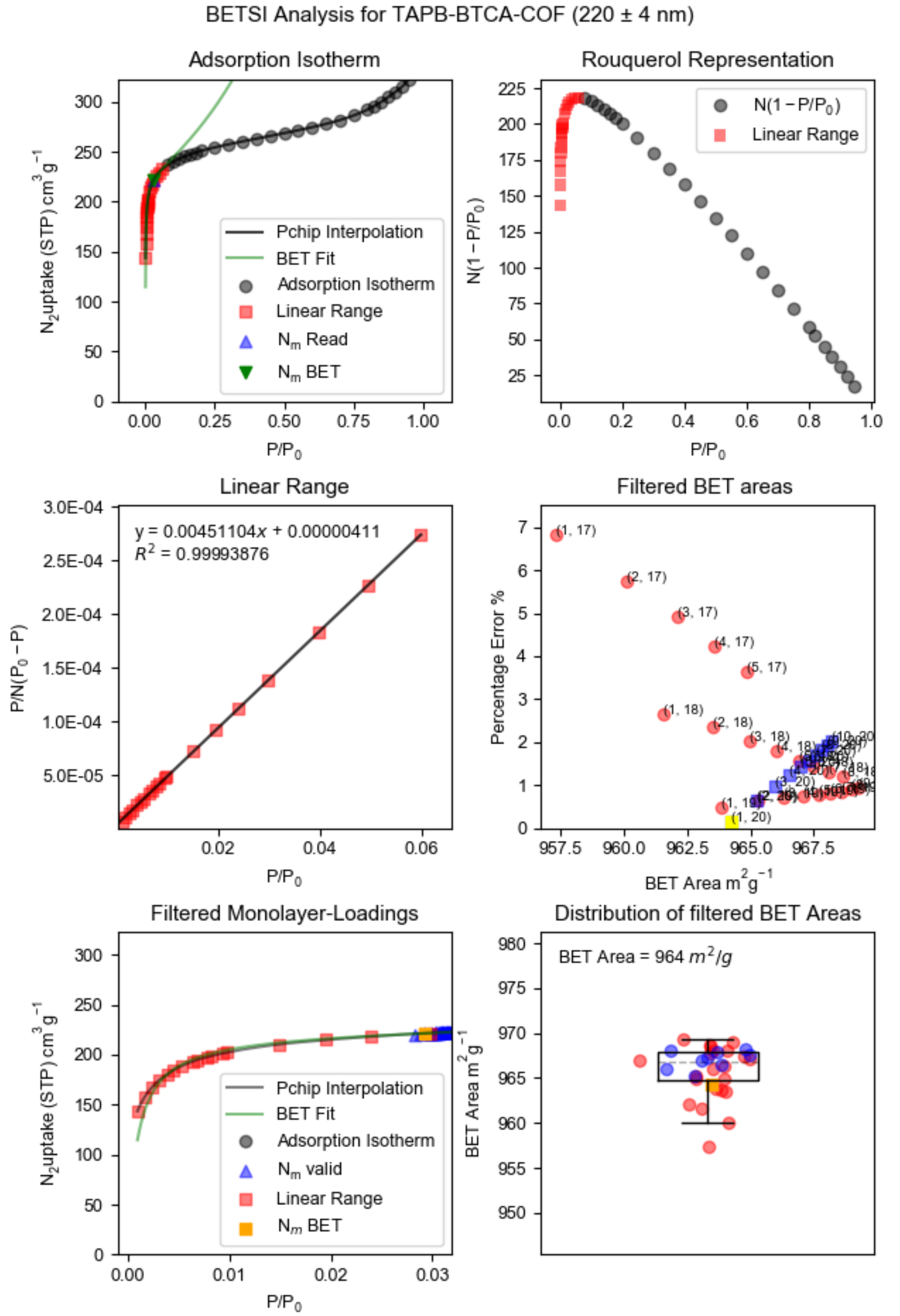

Figure S11. BETSI analysis of TAPB-BTCA-COF (220 ± 4 nm) (S_{BET} = 964 m^2 g^{-1}).

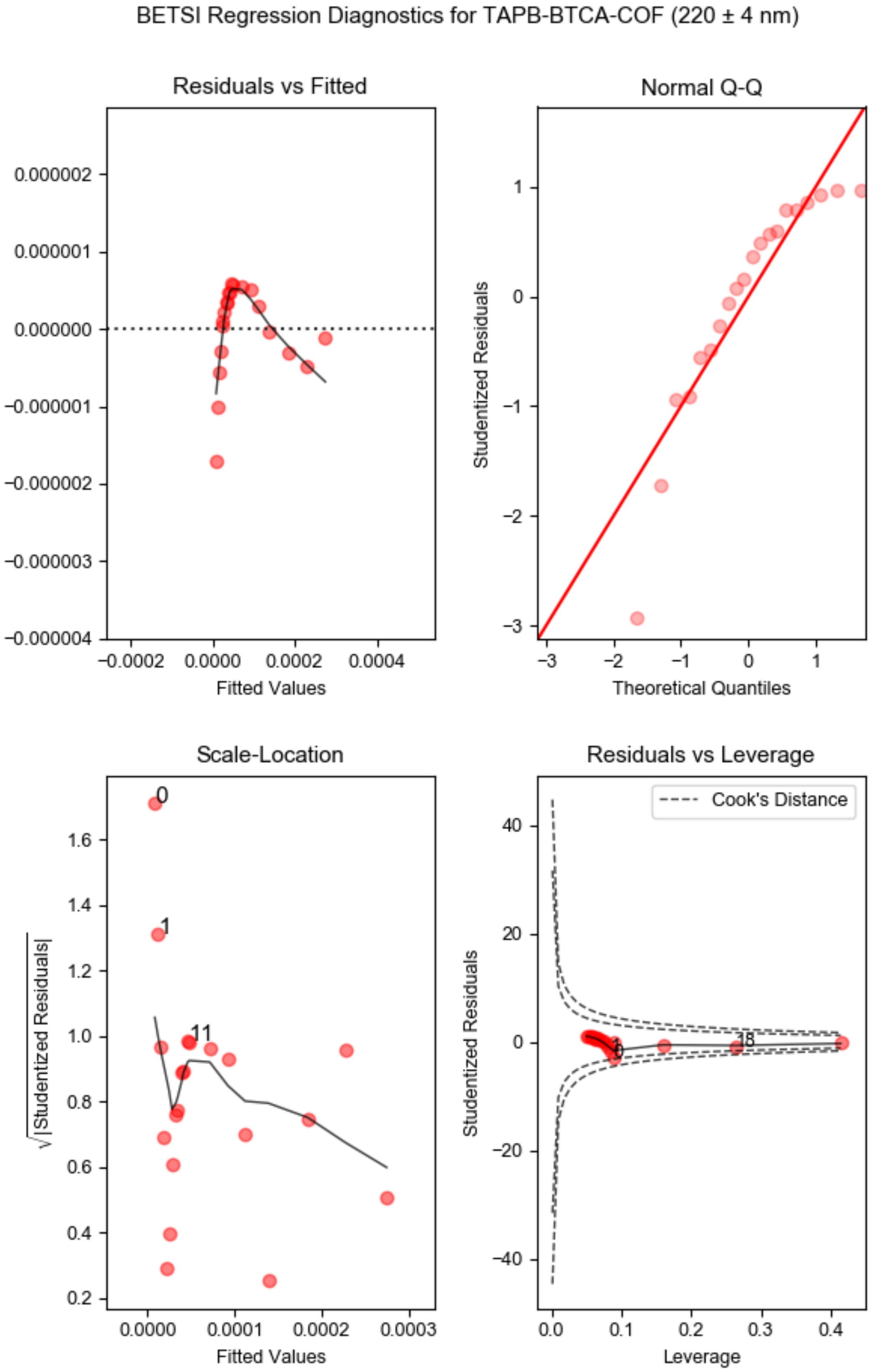

Figure S12. BETSI regression diagnostics for TAPB-BTCA-COF (220 ± 4 nm) (S_{BET} = 964 m^2 g^{-1}).

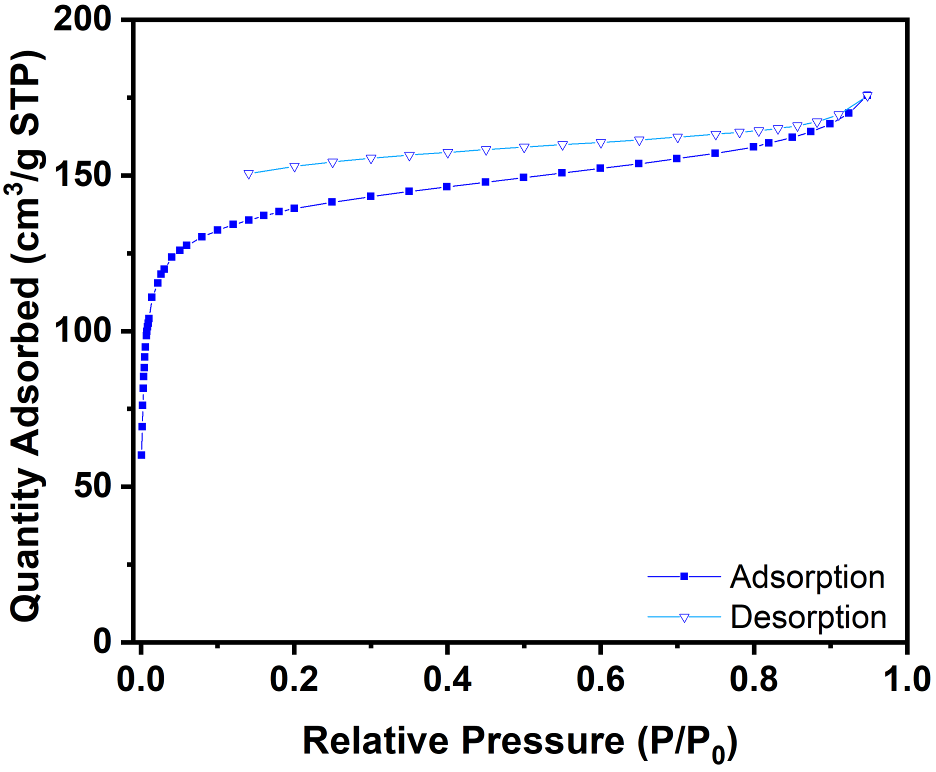

Figure S13. Nitrogen adsorption-desorption isotherm of TAPB-BTCA-COF (277 ± 5 nm) at 77 K.

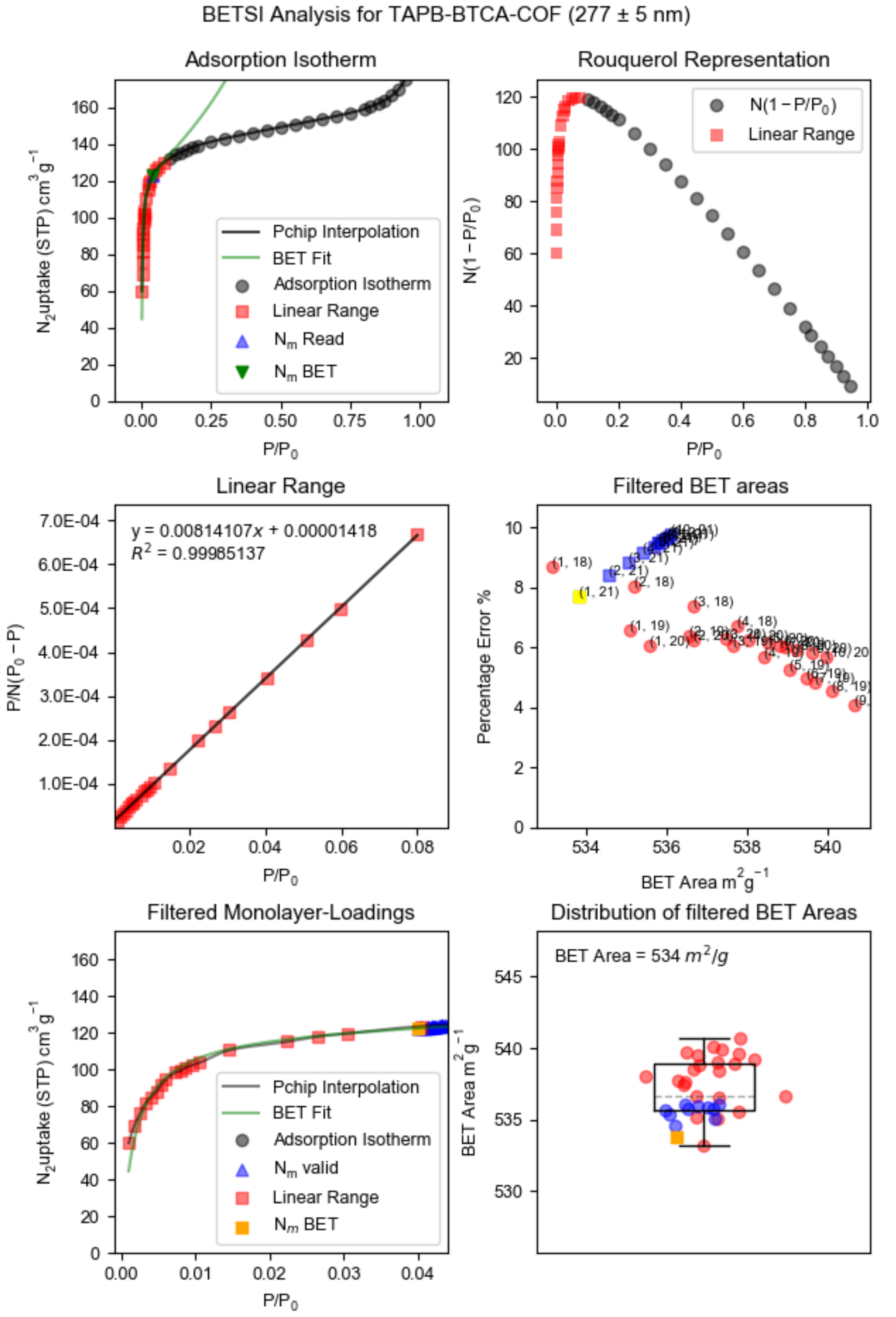

Figure S14. BETSI analysis of TAPB-BTCA-COF (277 ± 5 nm) (S_{BET} = 534 m^2 g^{-1}).

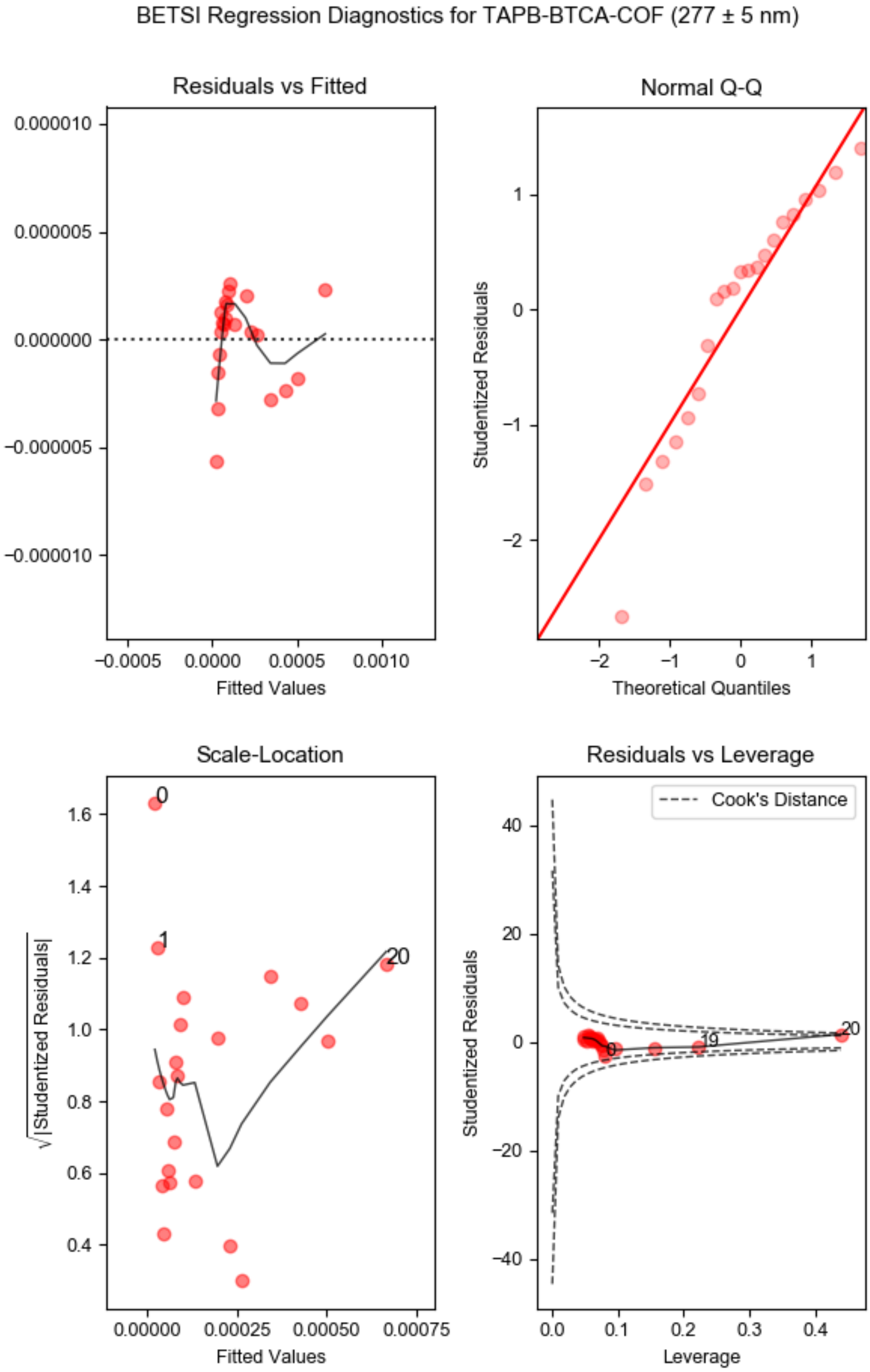

Figure S15. BETSI regression diagnostics for TAPB-BTCA-COF (277 ± 5 nm) (S_{BET} = 534 m^2 g^{-1}).

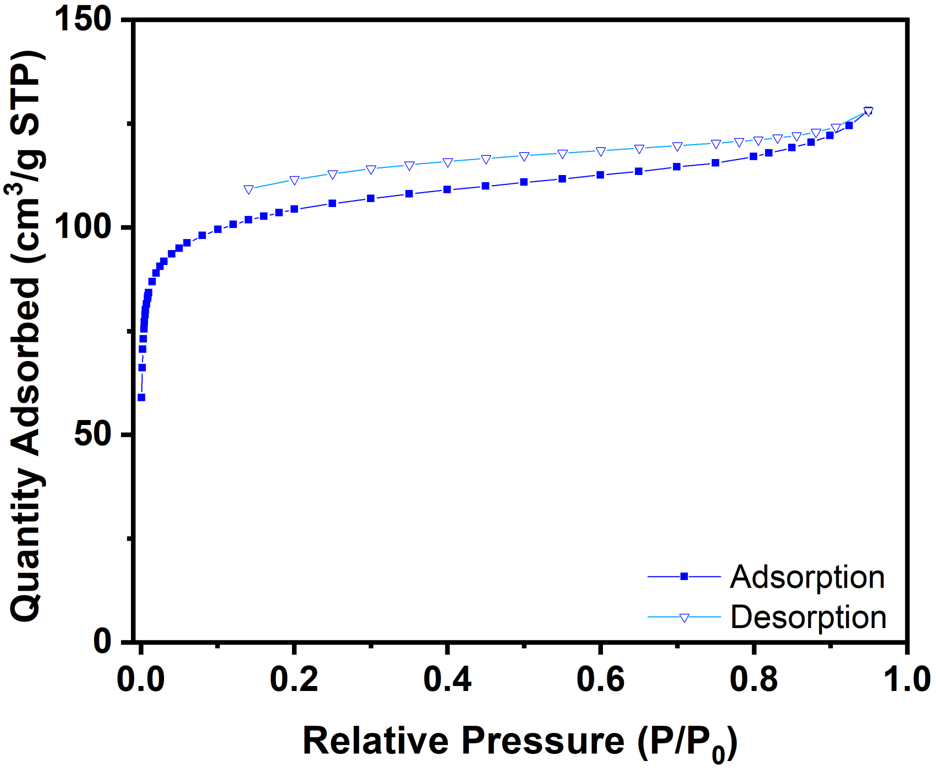

Figure S16. Nitrogen adsorption-desorption isotherm of TAPB-BTCA-COF (416 ± 7 nm) at 77 K.

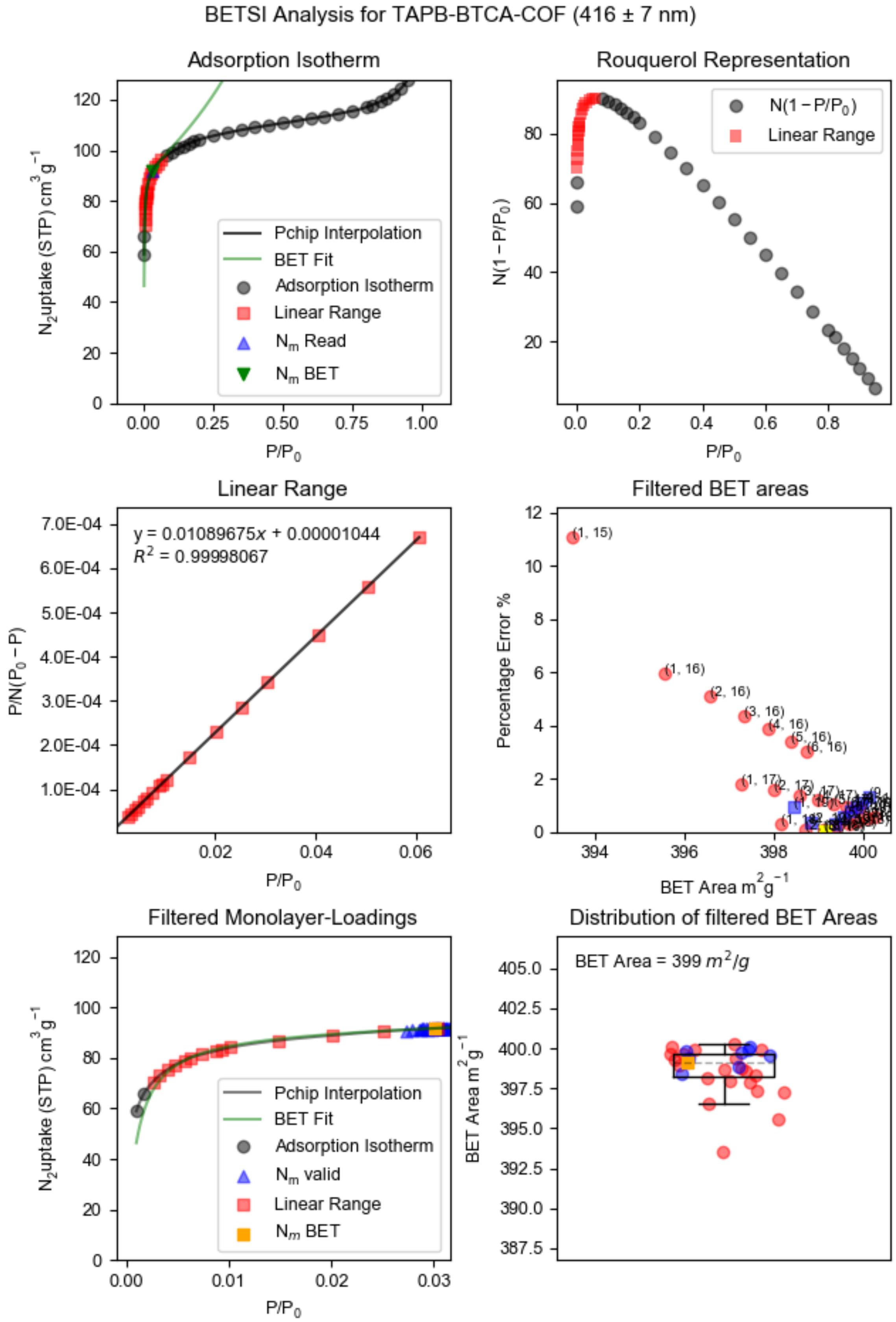

Figure S17. BETSI analysis of TAPB-BTCA-COF (416 ± 7 nm) (S_{BET} = 399 m² g⁻¹).

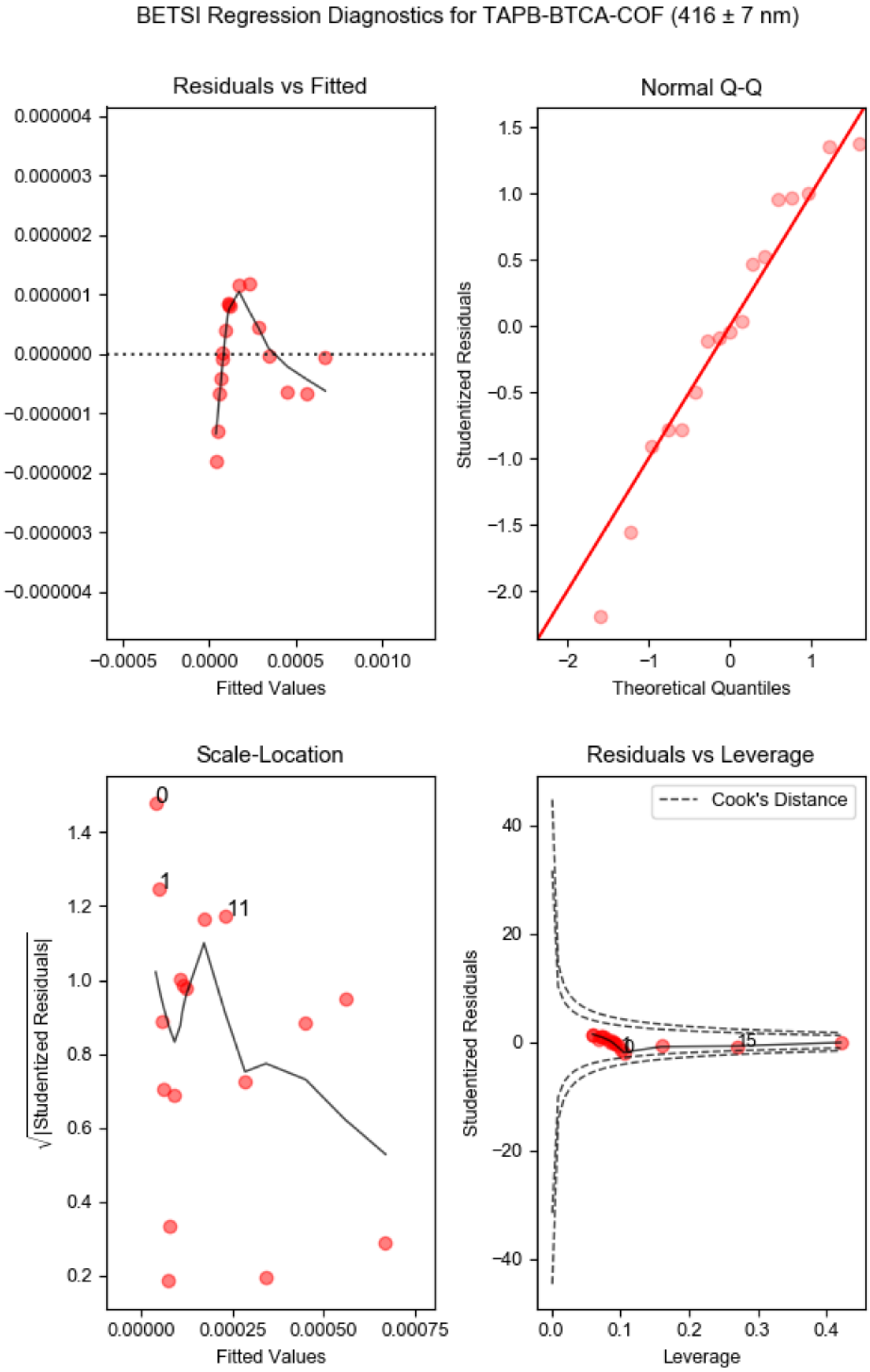

Figure S18. BETSI regression diagnostics for TAPB-BTCA-COF (416 ± 7 nm) (S_{BET} = 399 m^2 g^{-1}).

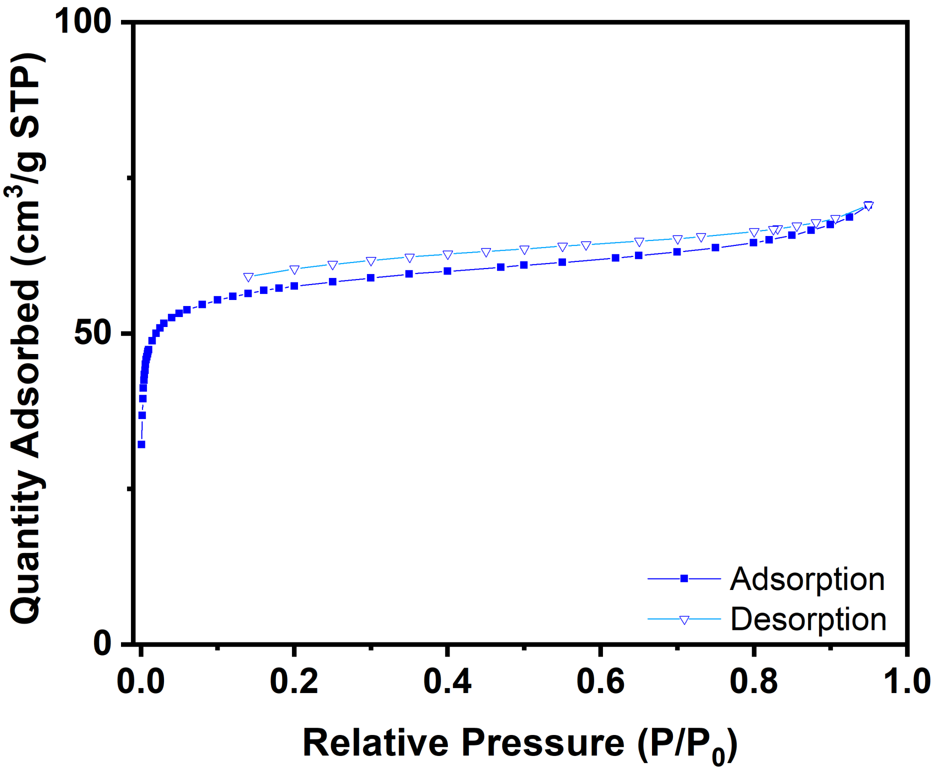

Figure S19. Nitrogen adsorption-desorption isotherm of TAPB-BTCA-COF (785 ± 12 nm) at 77 K.

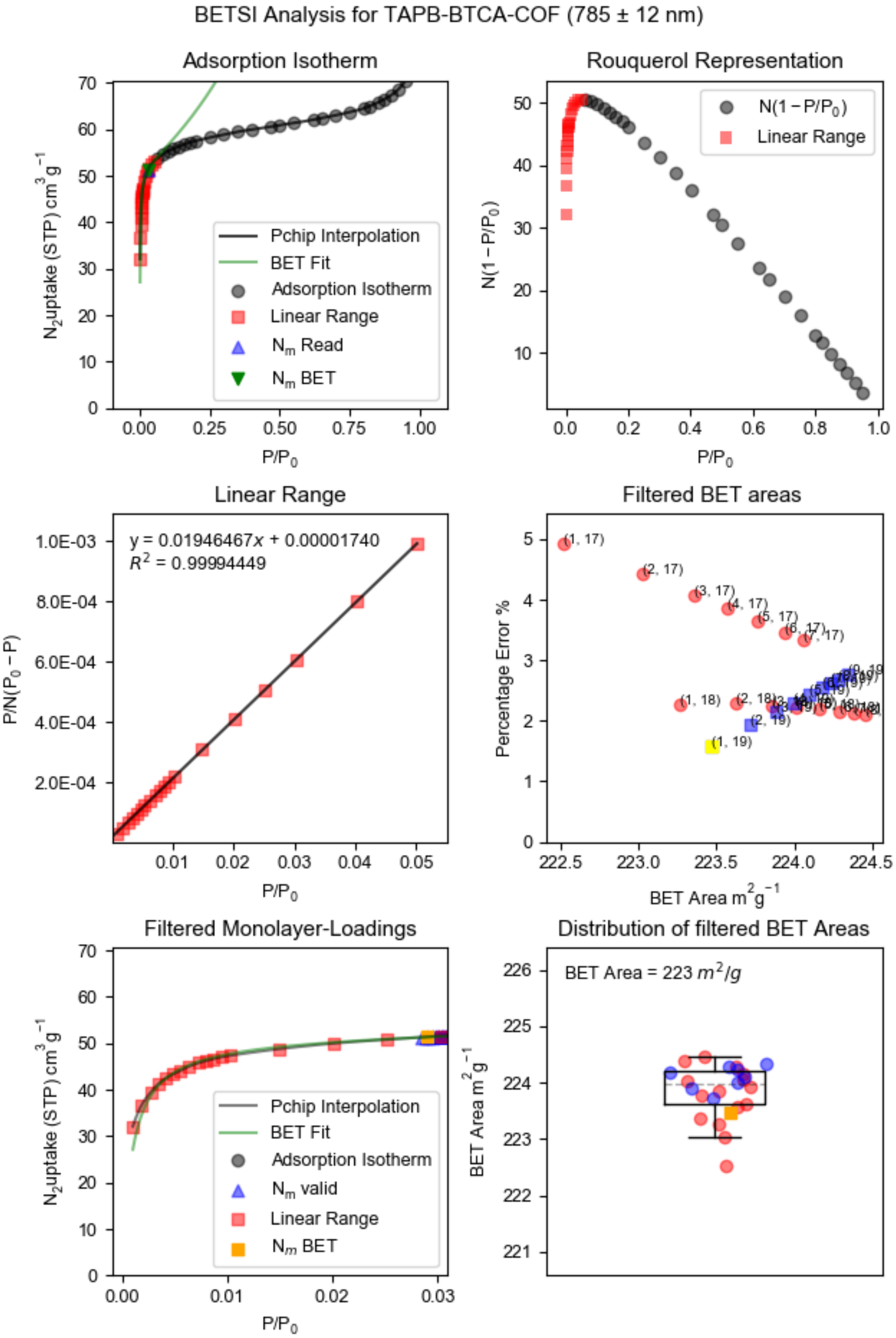

Figure S20. BETSI analysis of TAPB-BTCA-COF (785 ± 12 nm) (S_{BET} = 223 m² g⁻¹).

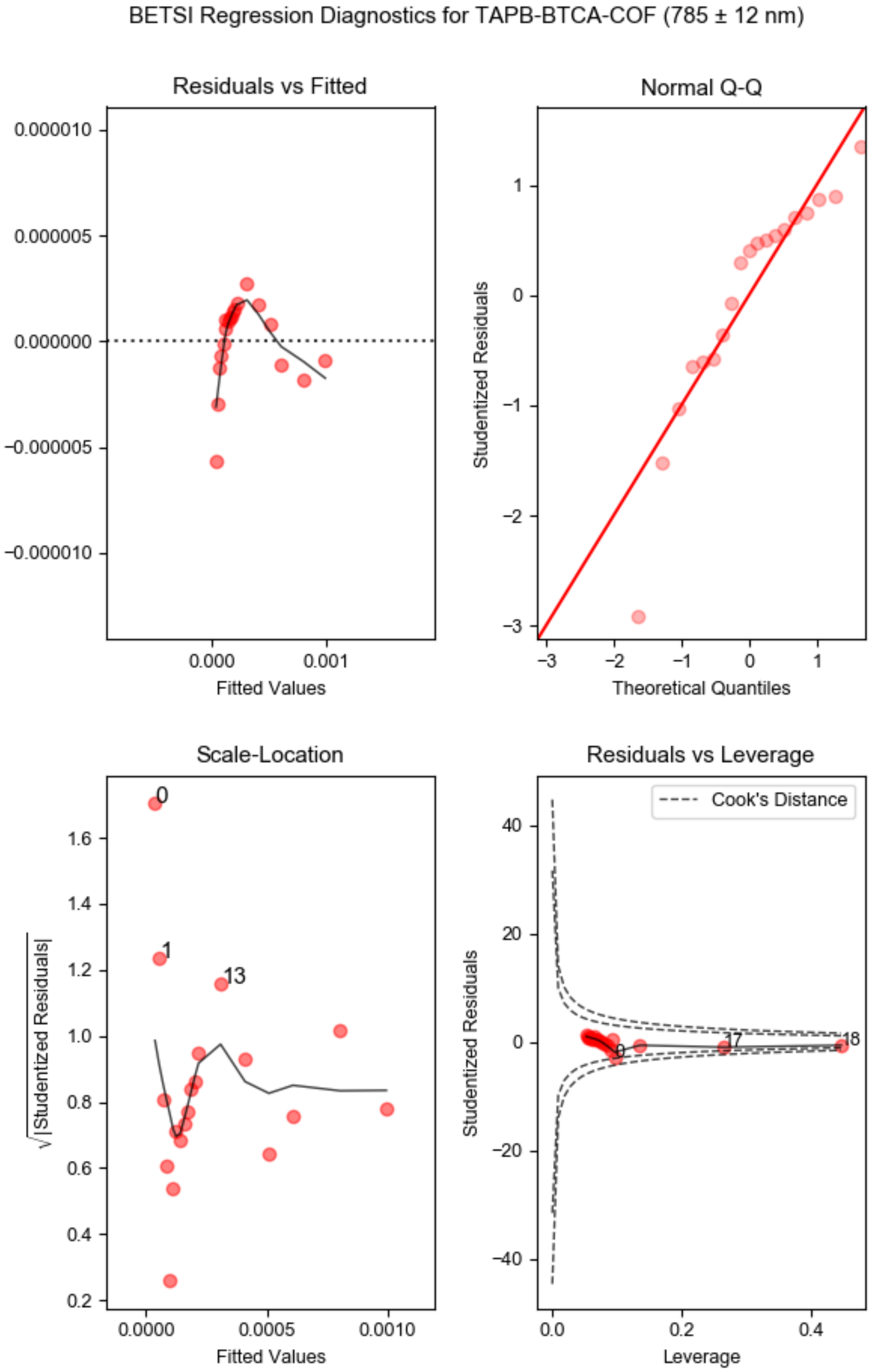

Figure S21. BETSI regression diagnostics for TAPB-BTCA-COF (785 ± 12 nm) (S_{BET} = 223 m^2 g^{-1}).

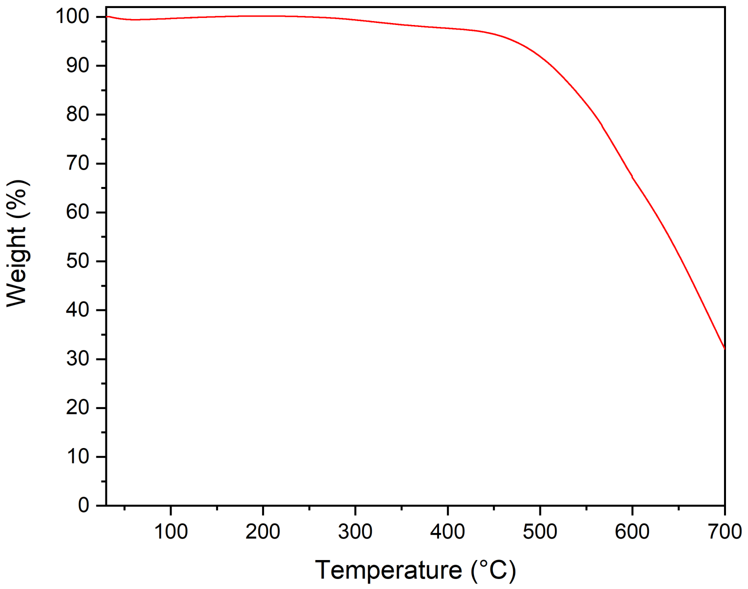

Figure S22. TGA analysis of PhCs made of TAPB-BTCA-COF particles. The weight loss around 500 °C is attributed to the decomposition of the COF particles.

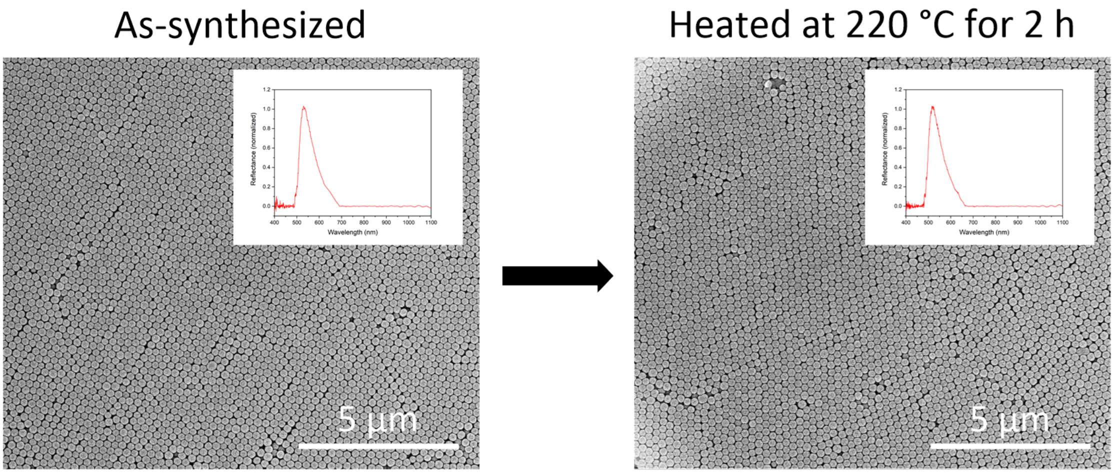

Figure S23. FE-SEM images of a PhC made of TAPB-BTCA-COF particles (203 ± 3 nm) before and after isothermal treatment at 220 °C for 2 h. These images show that, after isothermal treatment, the PhC retained its initial arrangement. Inset: optical reflectance spectra at normal incidence (θ=0°) of the PhC made of TAPB-BTCA-COF particles (203 ± 3 nm) before and after isothermal treatment at 220 °C for 2 h. The optical spectra further confirm that the superstructure remains highly ordered after isothermal treatment.

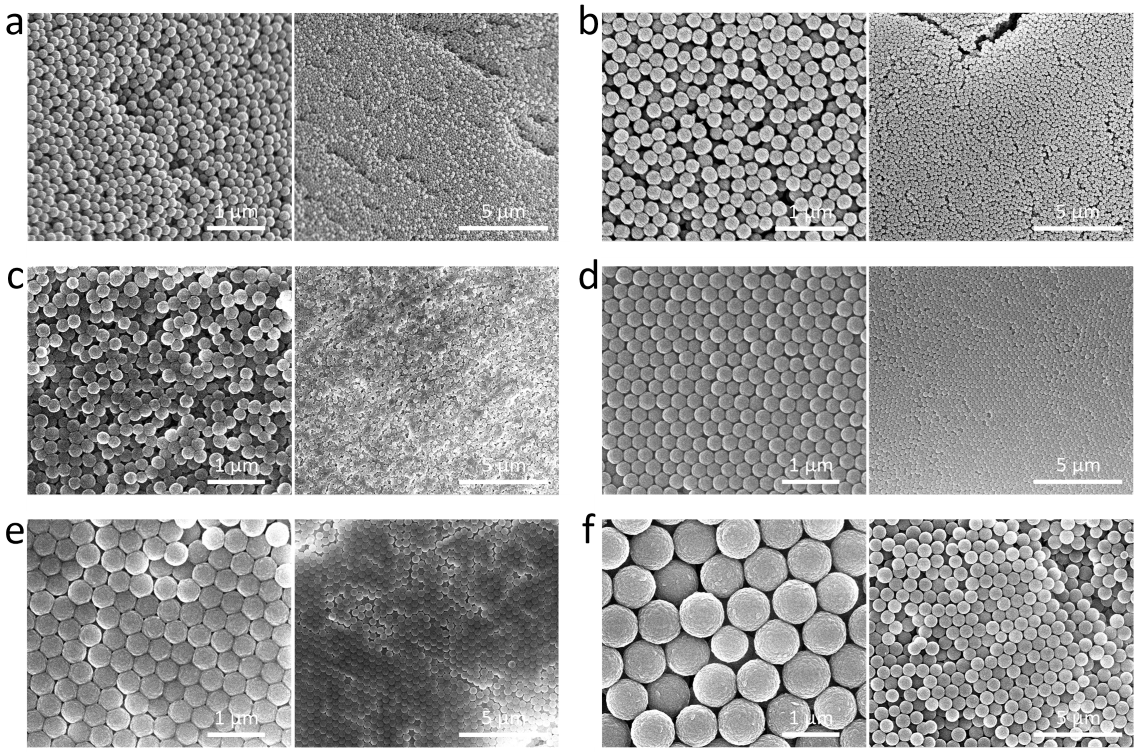

Figure S24. FE-SEM images of superstructures formed by evaporation-induced self-assembly of TAPB-BTCA-COF particles of different diameters, (a) 179 ± 6 nm, (b) 203 ± 3 nm, (c) 220 ± 4 nm, (d) 277 ± 5 nm, (e) 416 ± 7 nm, and (f) 785 ± 12 nm, at 140 °C.

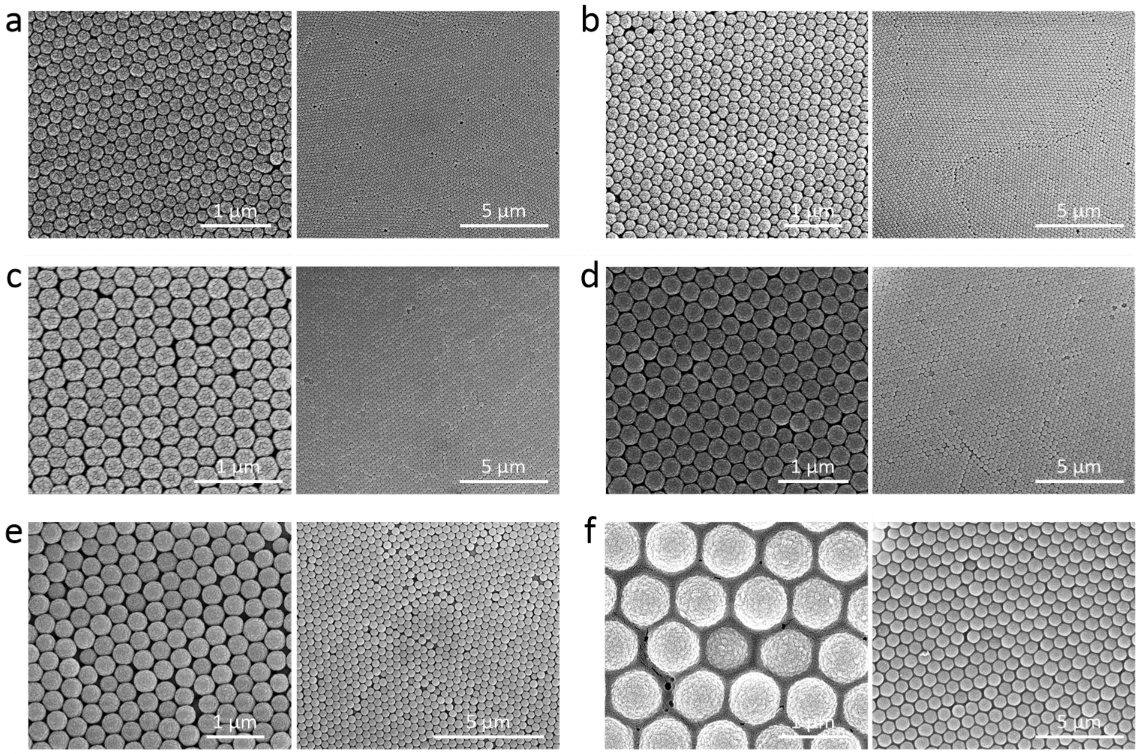

Figure S25. FE-SEM images of superstructures formed by evaporation-induced self-assembly of TAPB-BTCA-COF particles of different diameters, (a) 179 ± 6 nm, (b) 203 ± 3 nm, (c) 220 ± 4 nm, (d) 277 ± 5 nm, (e) 416 ± 7 nm, and (f) 785 ± 12 nm, at 100 °C.

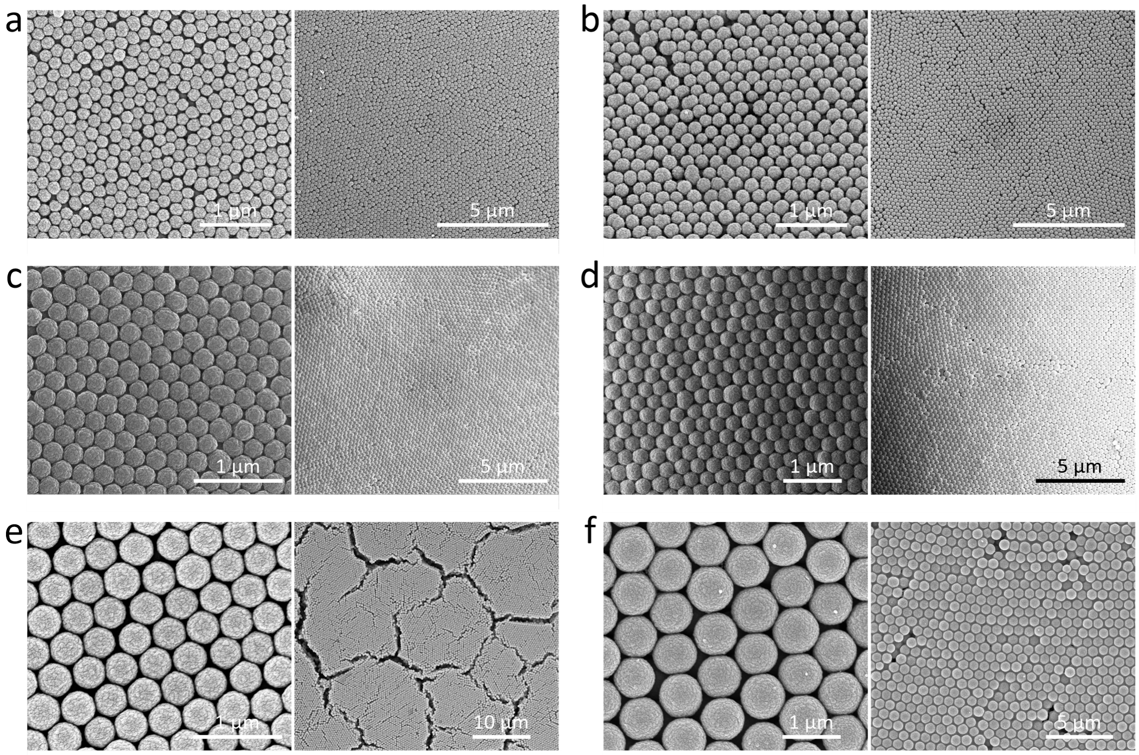

Figure S26. FE-SEM images of superstructures formed by evaporation-induced self-assembly of TAPB-BTCA-COF particles of different diameters, (a) 179 ± 6 nm, (b) 203 ± 3 nm, (c) 220 ± 4 nm, (d) 277 ± 5 nm, (e) 416 ± 7 nm, and (f) 785 ± 12 nm, at 65 °C.

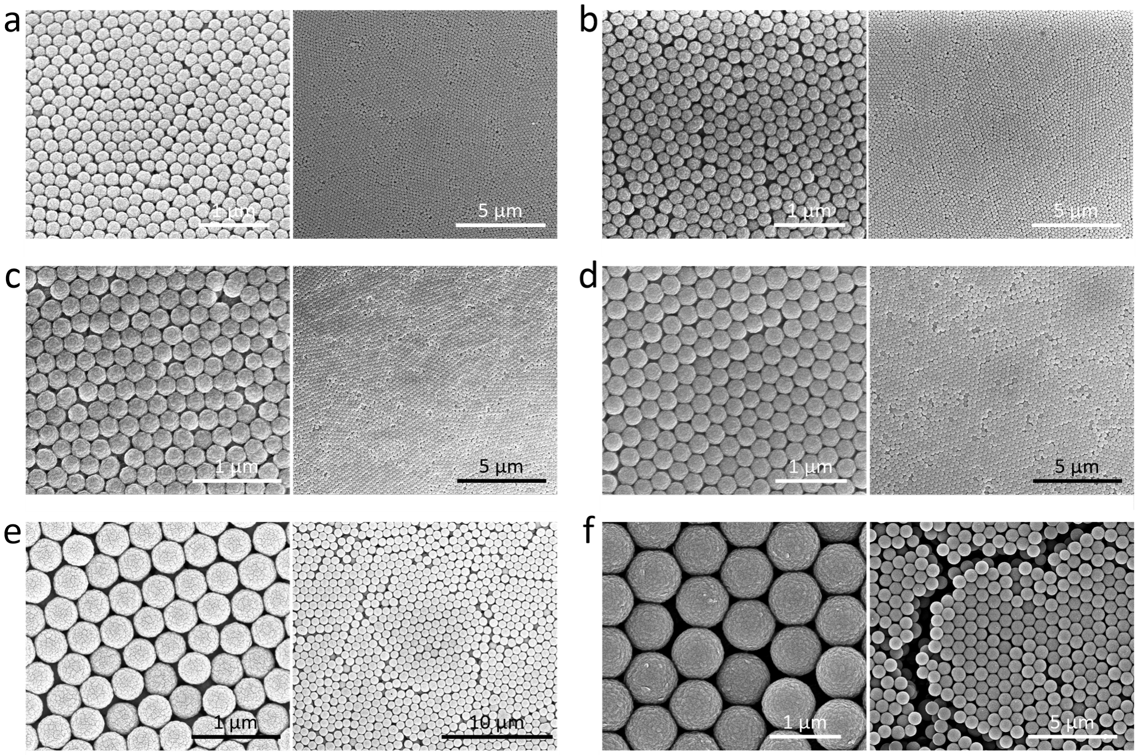

Figure S27. FE-SEM images of superstructures formed by evaporation-induced self-assembly of TAPB-BTCA-COF particles of different diameters, (a) 179 ± 6 nm, (b) 203 ± 3 nm, (c) 220 ± 4 nm, (d) 277 ± 5 nm, (e) 416 ± 7 nm, and (f) 785 ± 12 nm, at RT.

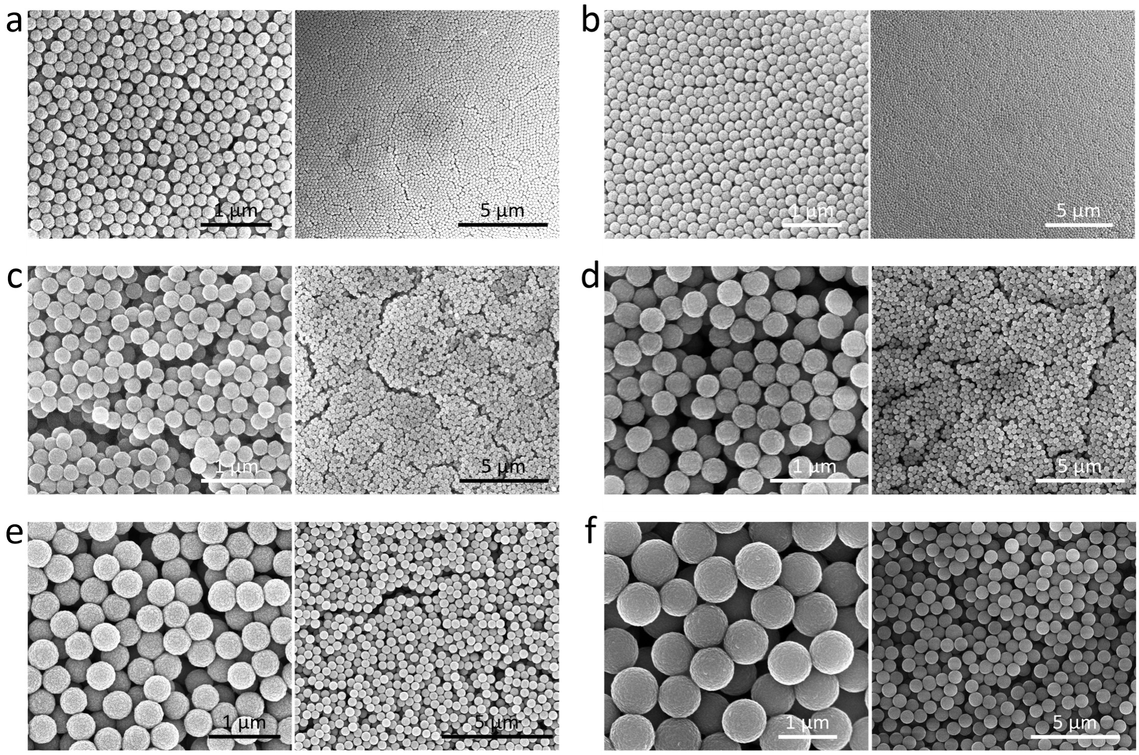

Figure S28. FE-SEM images of superstructures formed by evaporation-induced self-assembly of TAPB-BTCA-COF particles of different diameters, (a) 179 ± 6 nm, (b) 203 ± 3 nm, (c) 220 ± 4 nm, (d) 277 ± 5 nm, (e) 416 ± 7 nm, and (f) 785 ± 12 nm, at 2 °C.

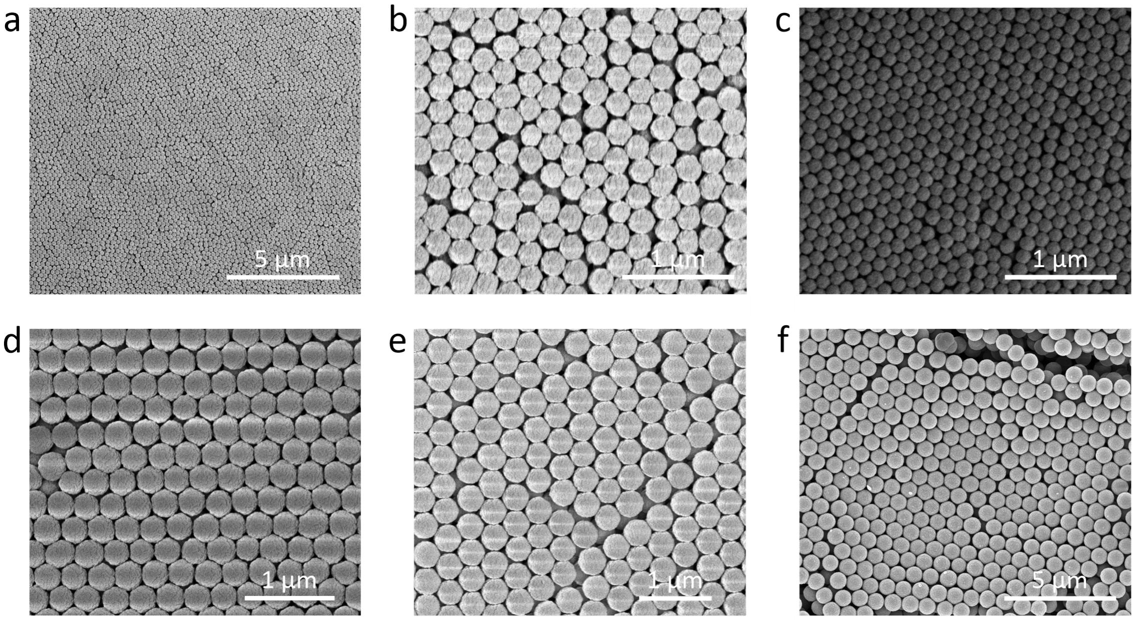

Figure S29. FE-SEM images of superstructures formed by heat-assisted vertical deposition self-assembly of TAPB-BTCA-COF particles of different diameters, (a) 179 ± 6 nm, (b) 203 ± 3 nm, (c) 220 ± 4 nm, (d) 277 ± 5 nm, (e) 416 ± 7 nm, and (f) 785 ± 12 nm, at 45 °C.

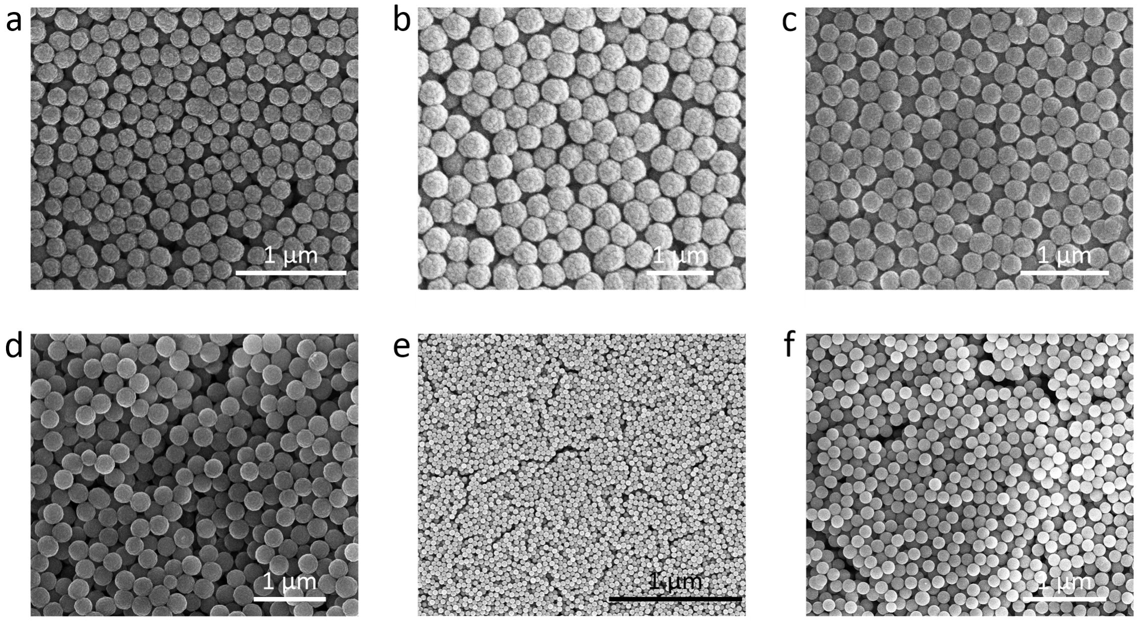

Figure S30. FE-SEM images of superstructures formed by centrifugation-based self-assembly of TAPB-BTCA-COF particles of different diameters, (a) 179 ± 6 nm, (b) 203 ± 3 nm, (c) 220 ± 4 nm, (d) 277 ± 5 nm, (e) 416 ± 7 nm, and (f) 785 ± 12 nm.

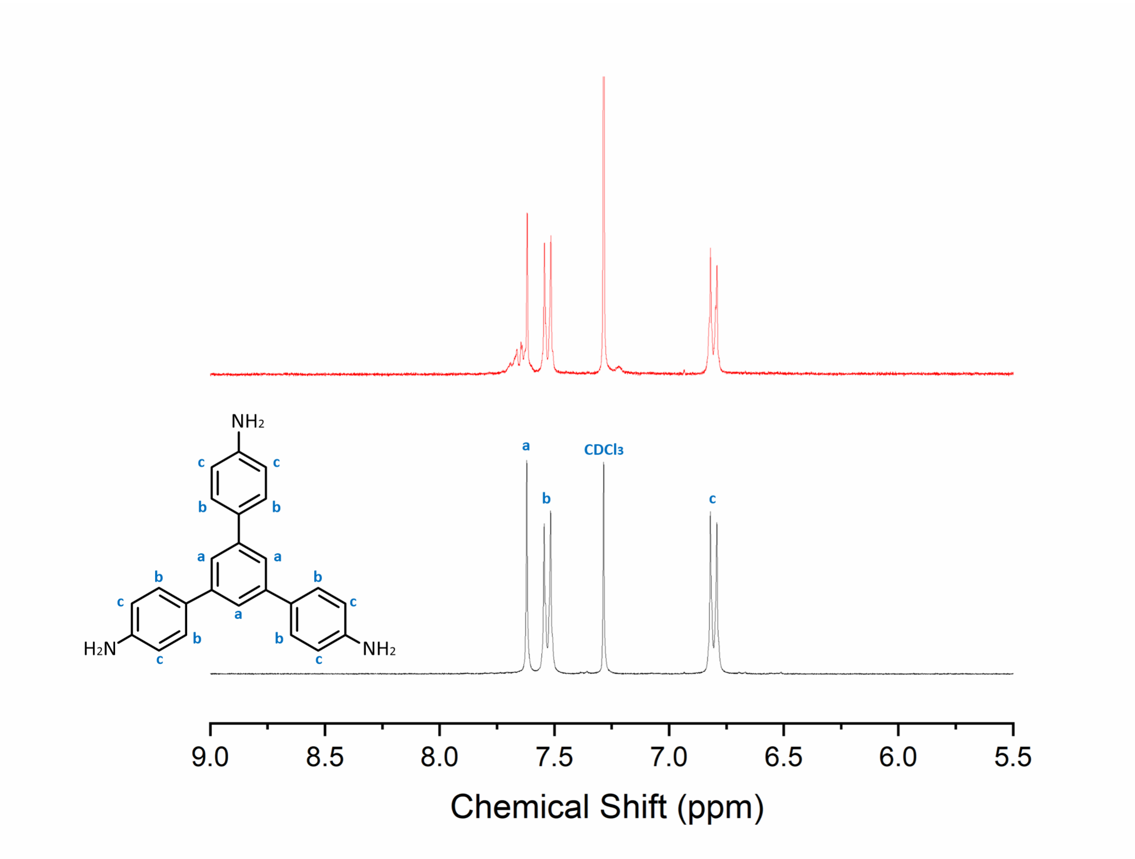

Figure S31. ^{1}H NMR spectra (300 MHz, $CDCl_3$) of TAPB (black) and the supernatant resulting from the centrifugation of TAPB-BTCA-COF particles (277 ± 5 nm) (red).

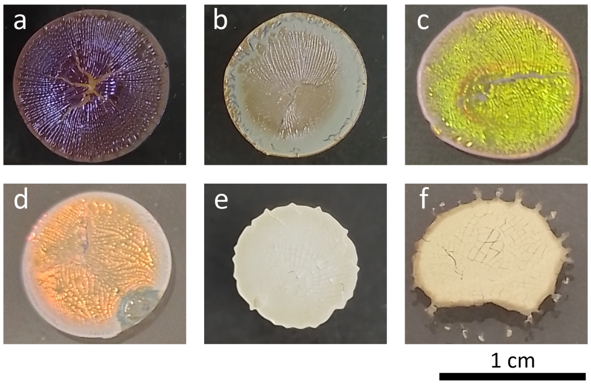

Figure S32. (a-f) Photographs of the self-assembled PhCs made of TAPB-BTCA-COF particles of different sizes: (a) 179 ± 6 nm; (b) 203 ± 3 nm; (c) 220 ± 4 nm; (d) 277 ± 5 nm; (e) 416 ± 7 nm; and (f) 785 ± 12 nm. Although the color of the COF-based PhCs is influenced by the yellow-brown color of the COF particles, the typical angle-dependent opalescence of colloidal PhCs is appreciated.

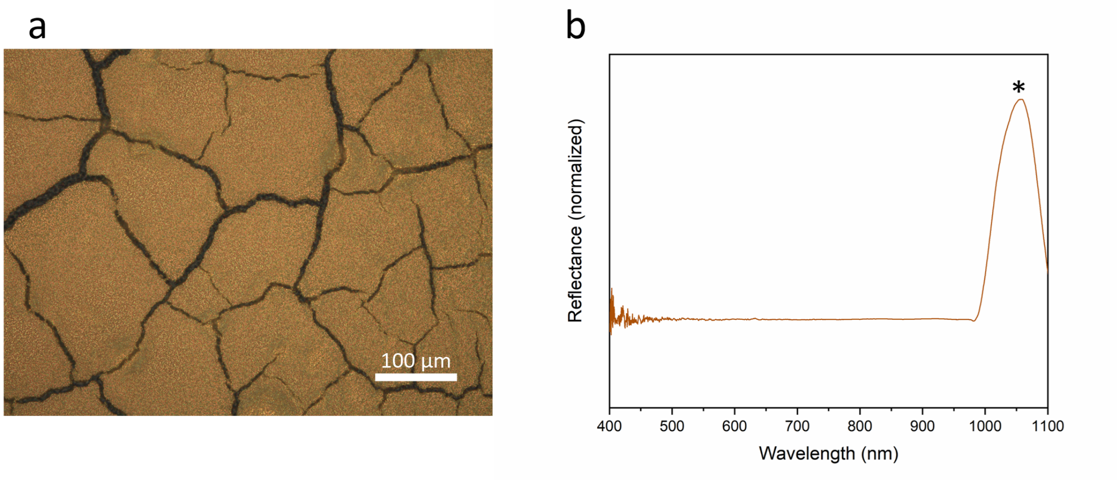

Figure S33. (a) Optical image and (b) optical reflectance spectrum at normal incidence (θ = 0°) of the self-assembled photonic crystals made of TAPB-BTCA-COF particles of diameter: 785 ± 12 nm. The peak marked with an asterisk is assumed to correspond to a high-energy photon band. The Bragg reflection maximum is expected to appear around 1600-1700 nm, which is outside the reading range of the spectrophotometer.

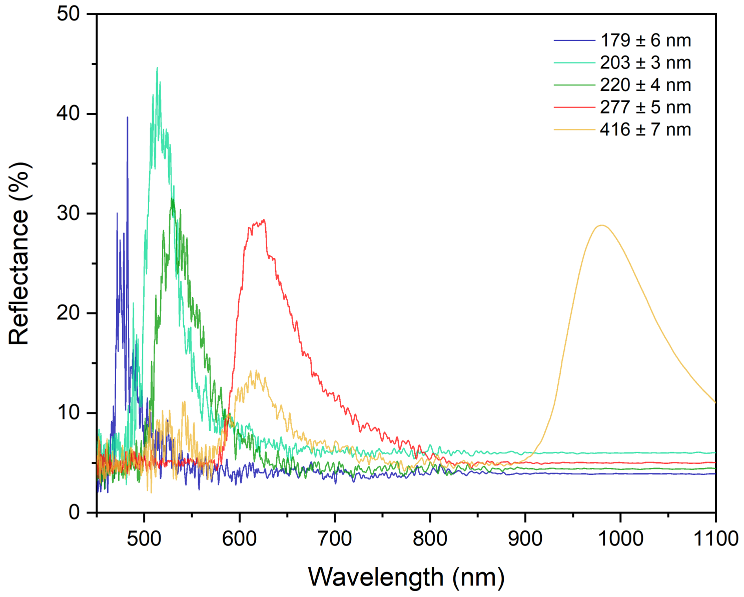

Figure S34. Optical reflectance at θ = 0° for the PhCs made of TAPB-BTCA-COF particles of different sizes: 179 ± 6 nm (violet); 203 ± 3 nm (sky blue); 220 ± 4 nm (green); 277 ± 5 nm (red); 416 ± 7 nm (yellow); and 785 ± 12 nm (orange).

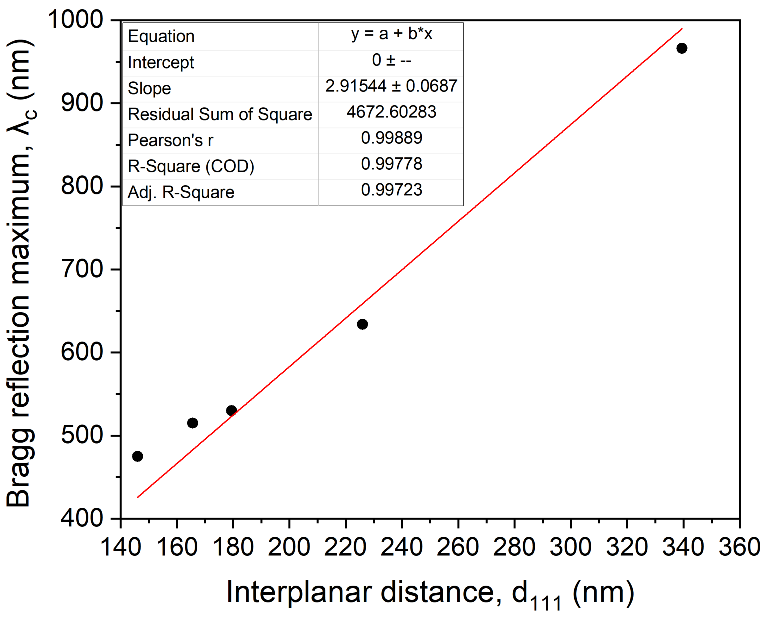

Figure S35. Bragg reflection maximum (λ_c) plotted against the interplanar distance (d_{111}) and fitted to the Bragg-Snell law. Inset: fitting function and correlation coefficient.

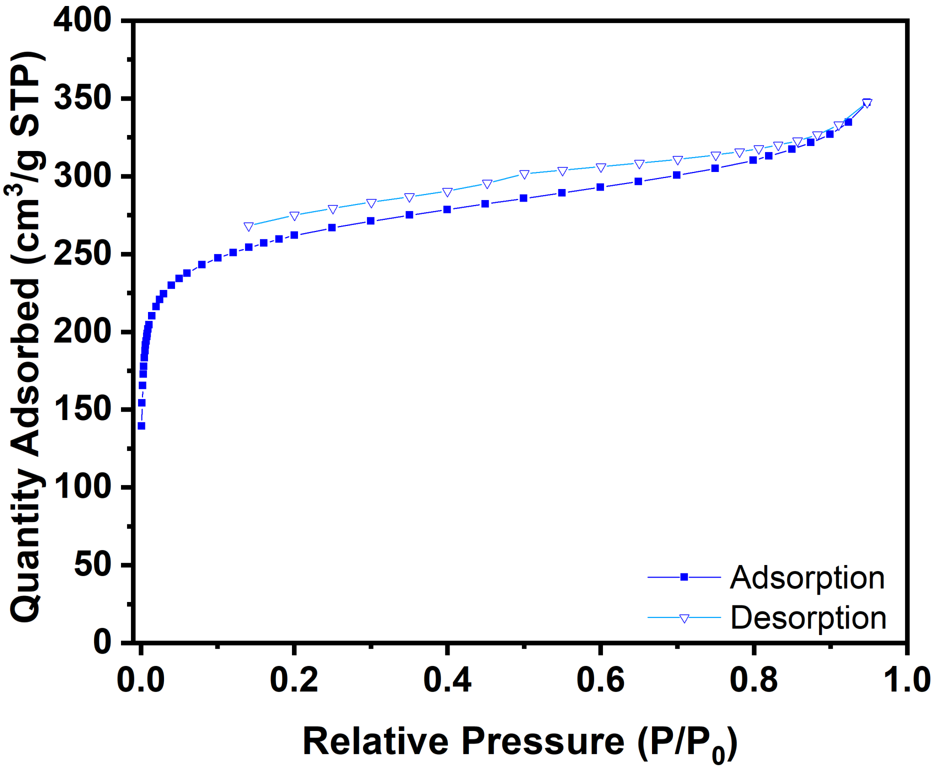

Figure S36. Nitrogen adsorption-desorption isotherm of photonic crystals made of TAPB-BTCA-COF particles (179 ± 6 nm) at 77 K.

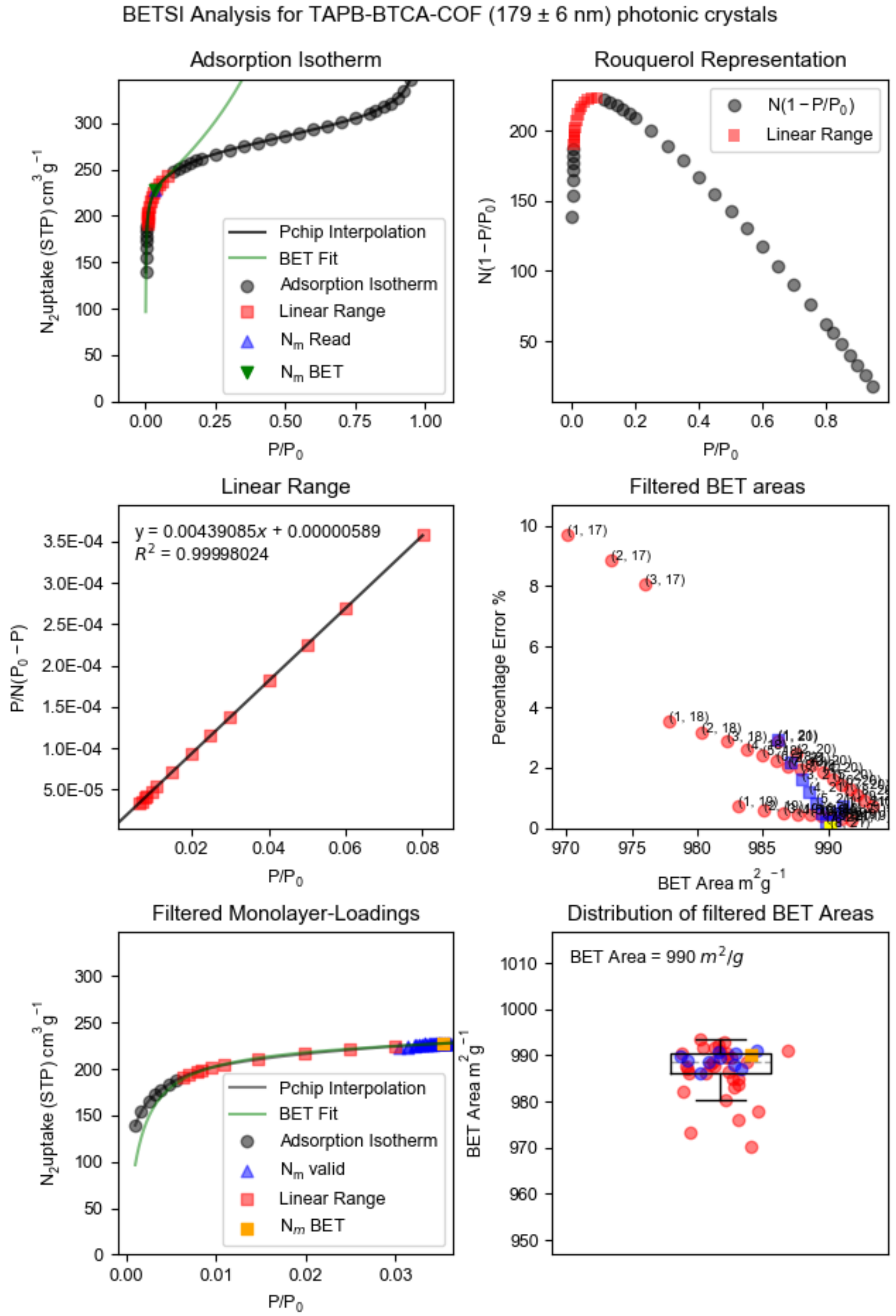

Figure S37. BETSI analysis of photonic crystals made of TAPB-BTCA-COF particles (179 ± 6 nm) (S_{BET} = 990 m² g⁻¹).

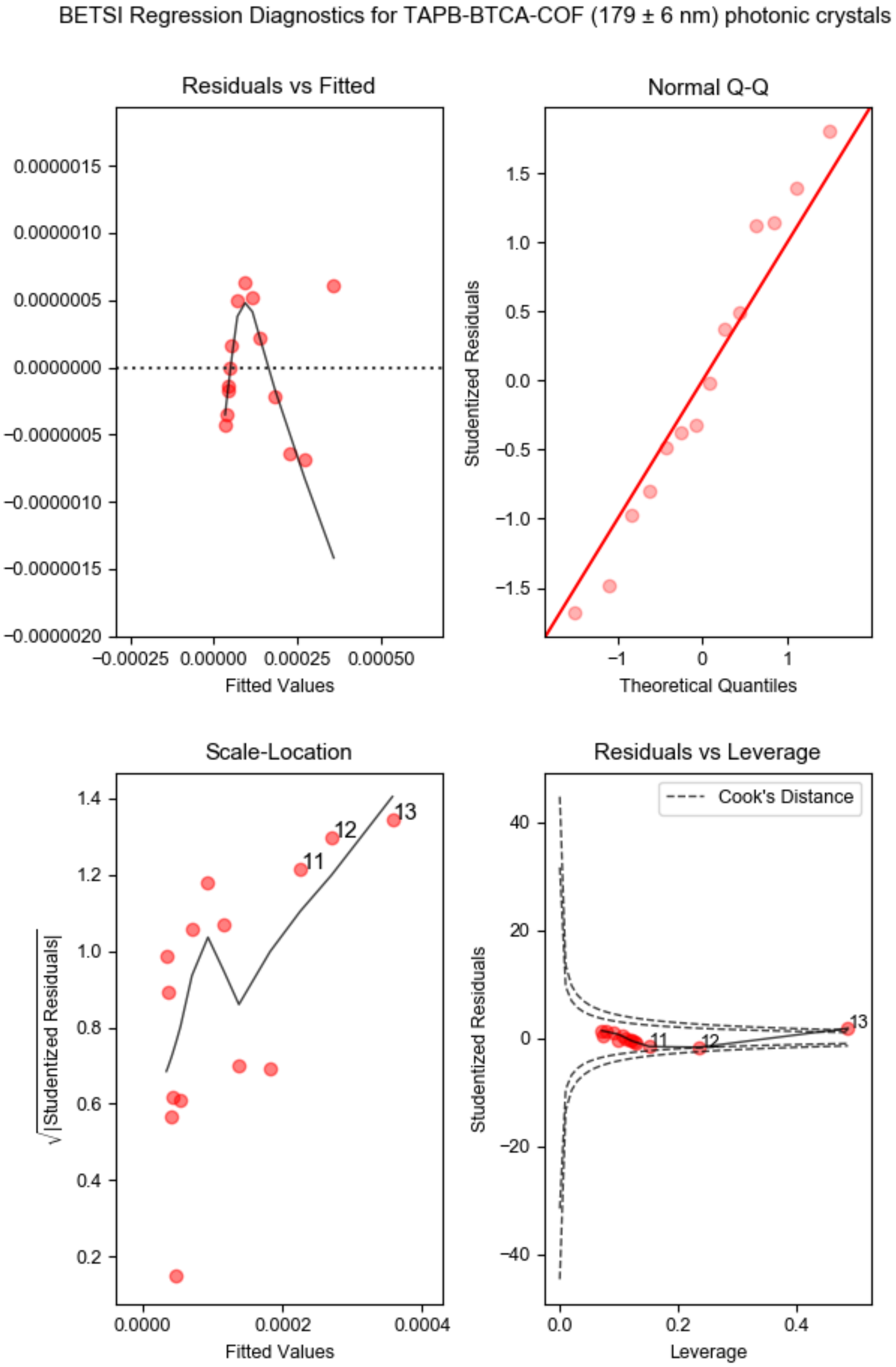

Figure S38. BETSI regression diagnostics for photonic crystals made of TAPB-BTCA-COF particles (179 ± 6 nm) (S_{BET} = 990 m^2 g^{-1}).

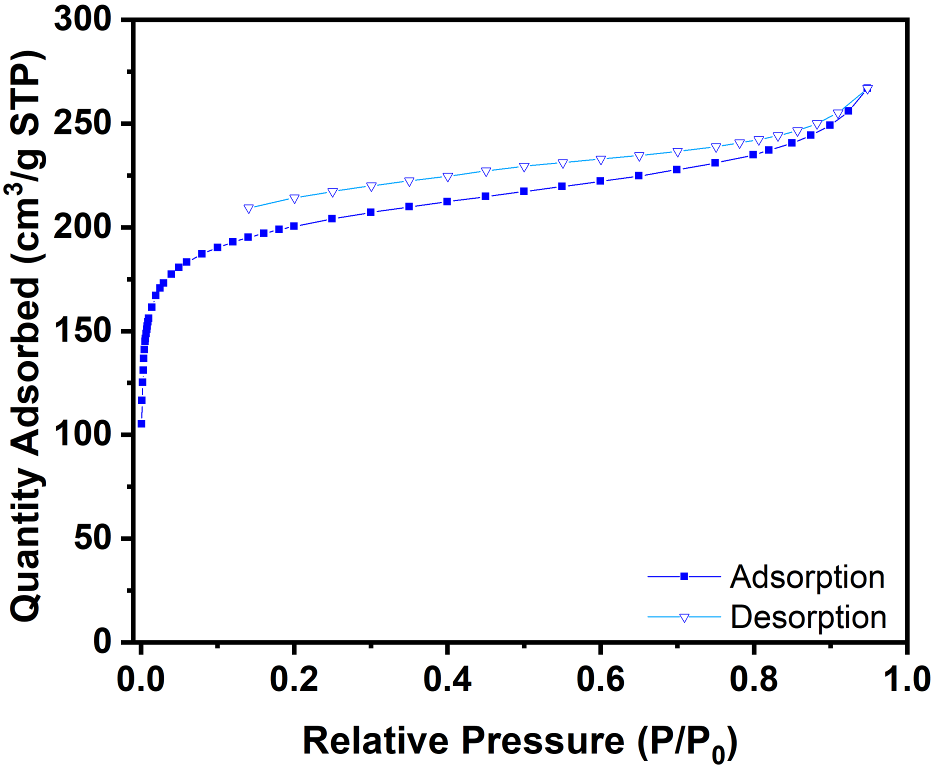

Figure S39. Nitrogen adsorption-desorption isotherm of photonic crystals made of TAPB-BTCA-COF particles (203 ± 3 nm) at 77 K.

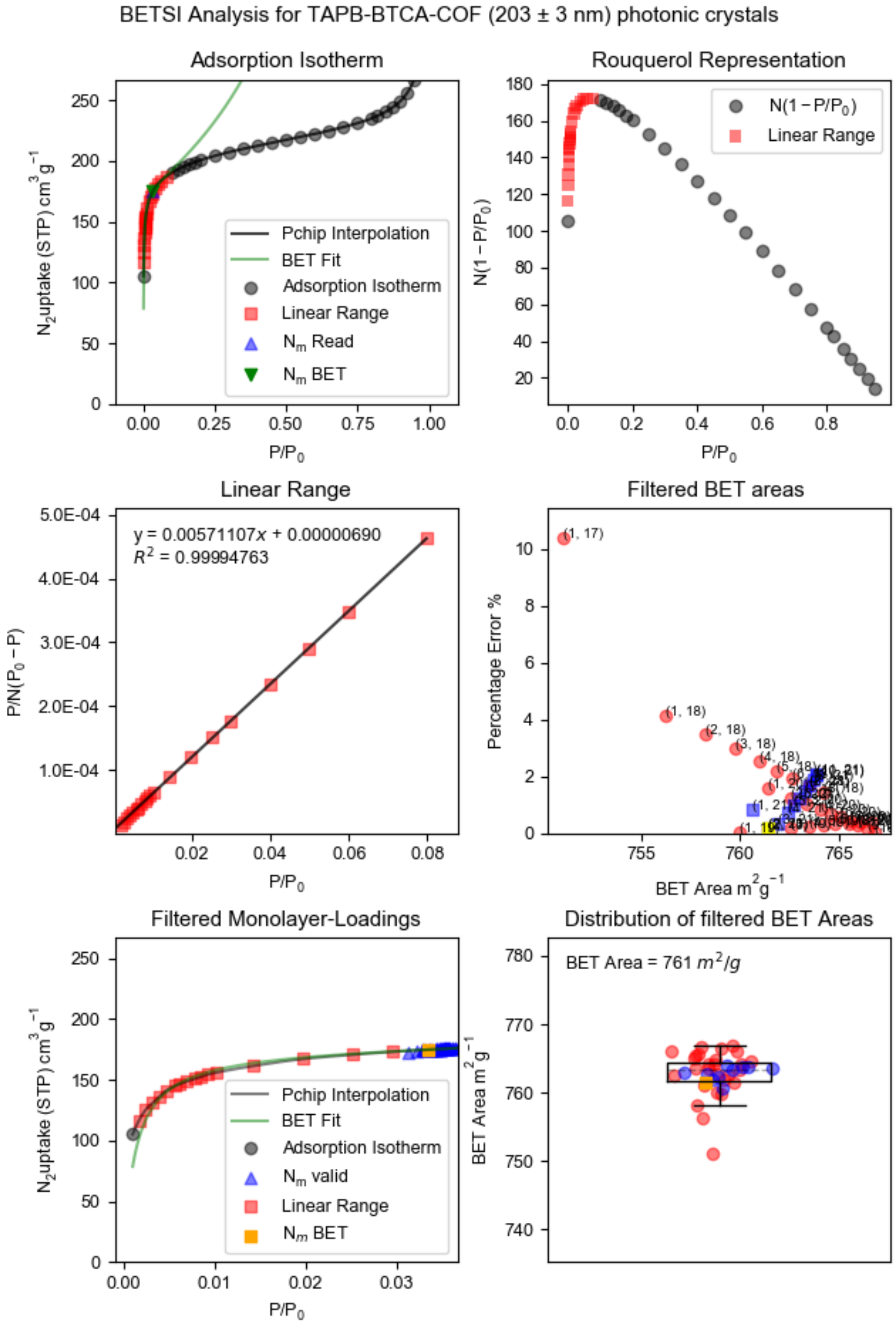

Figure S40. BETSI analysis of photonic crystals made of TAPB-BTCA-COF particles (203 ± 3 nm) (S_{BET} = 761 m^2 g^{-1}).

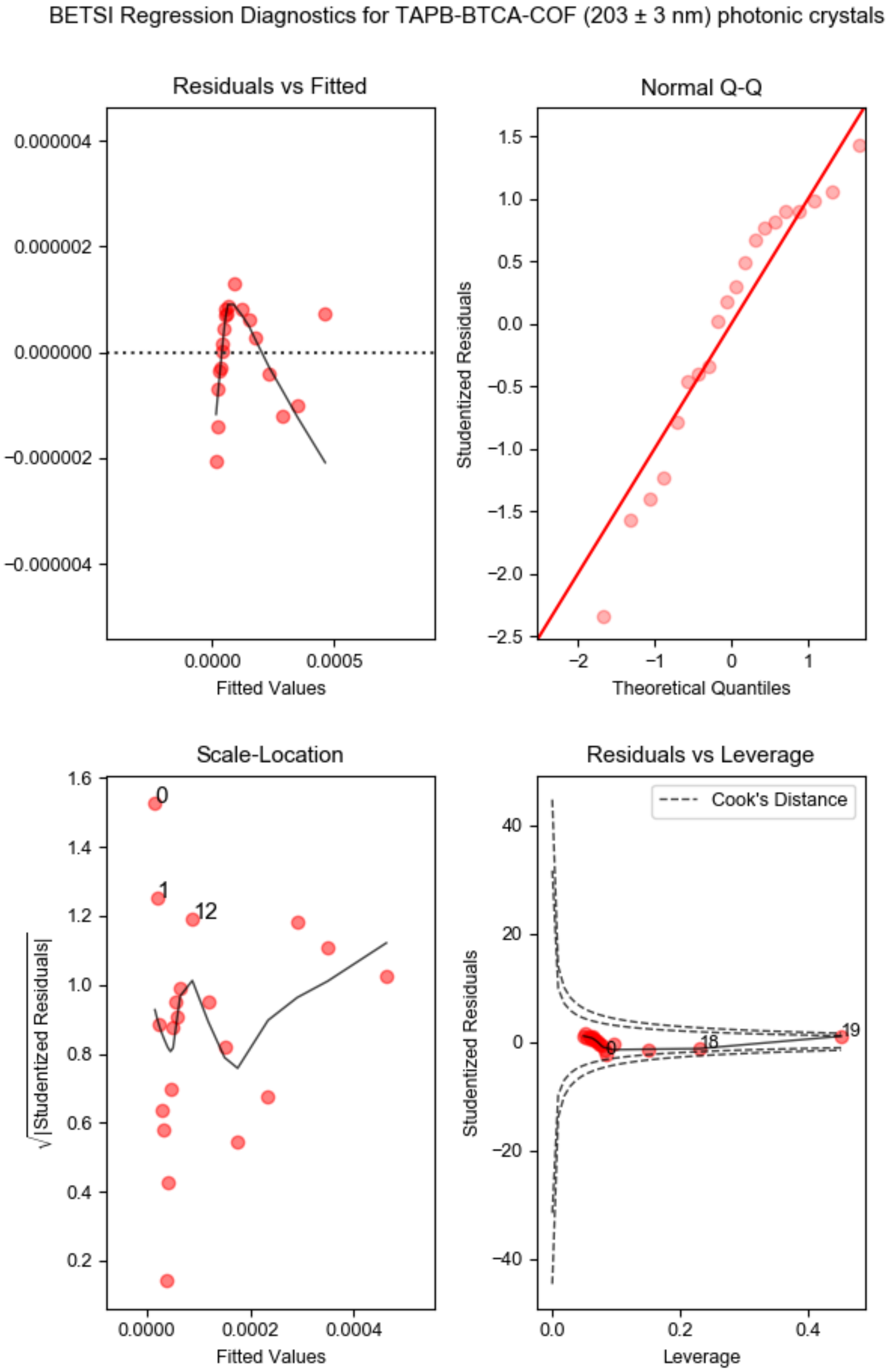

Figure S41. BETSI regression diagnostics for photonic crystals made of TAPB-BTCA-COF particles (203 ± 3 nm) (S_{BET} = 761 m^2 g^{-1}).

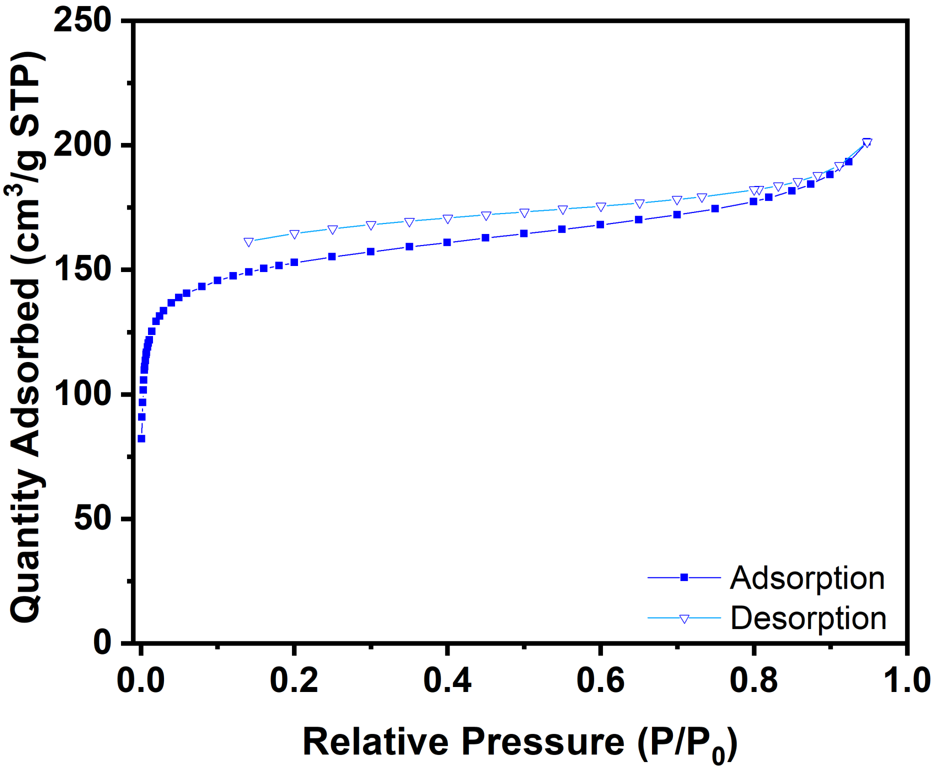

Figure S42. Nitrogen adsorption-desorption isotherm of photonic crystals made of TAPB-BTCA-COF particles (220 ± 4 nm) at 77 K.

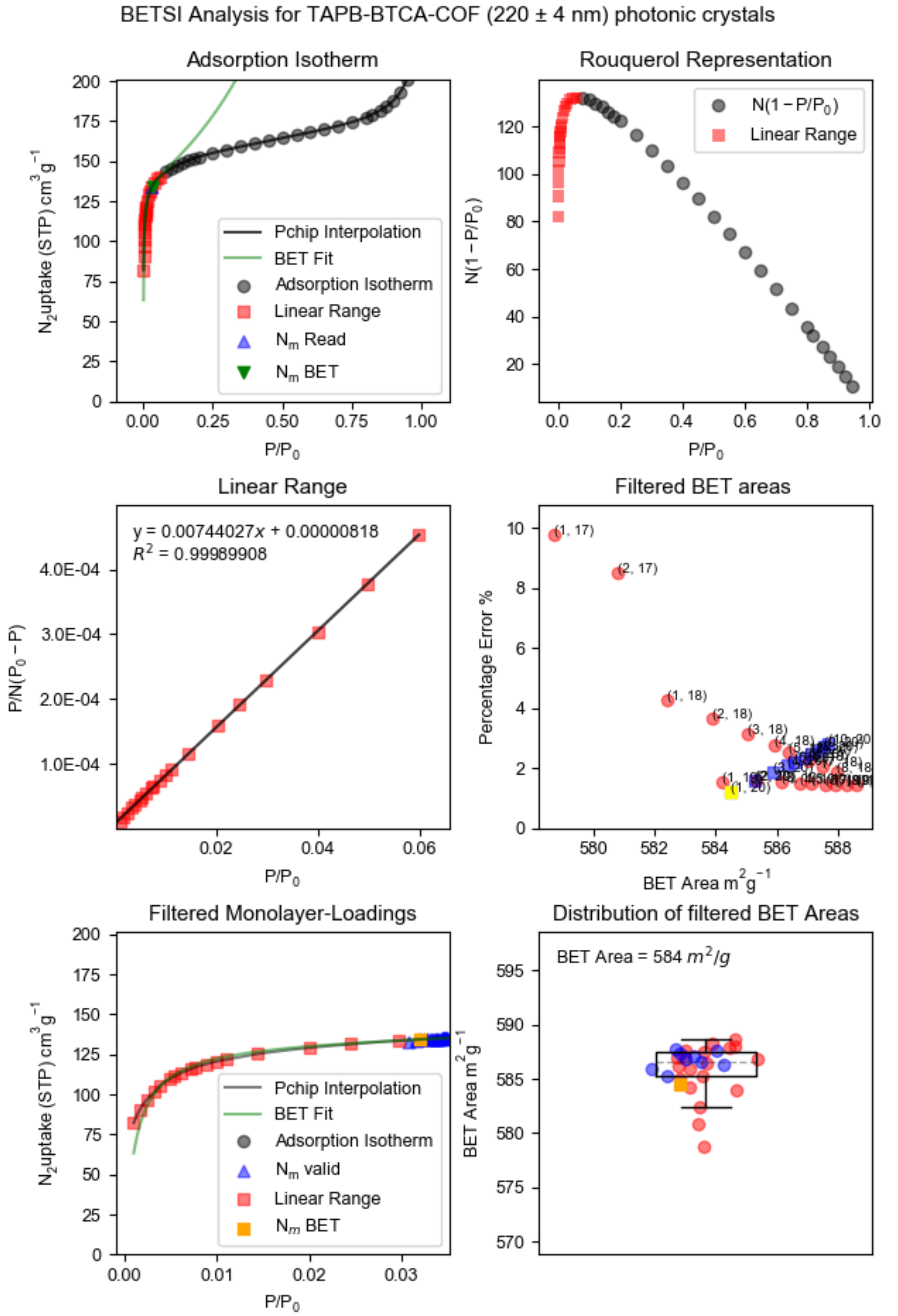

Figure S43. BETSI analysis of photonic crystals made of TAPB-BTCA-COF particles (220 ± 4 nm) (S_{BET} = 584 m^2 g^{-1}).

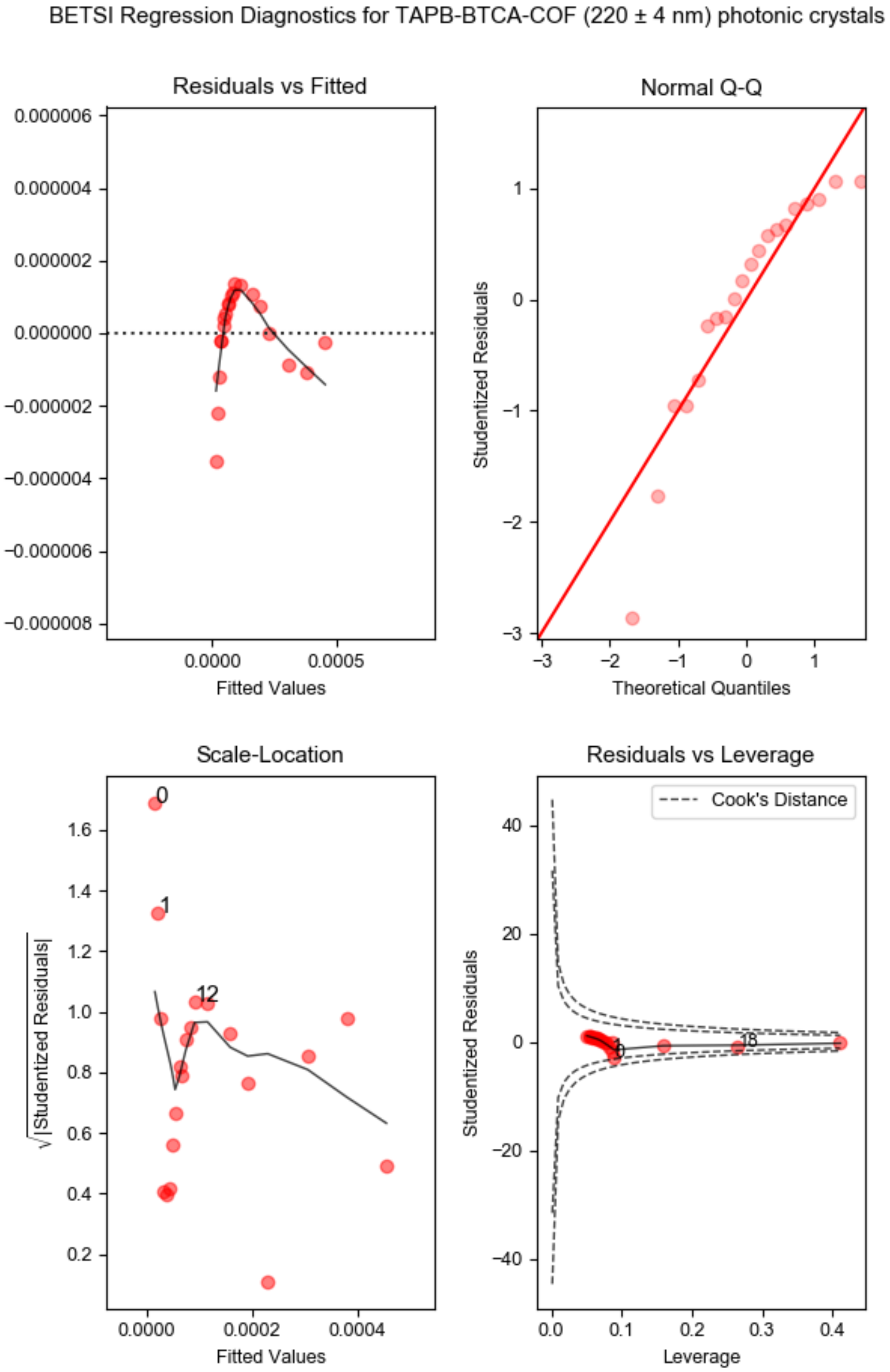

Figure S44. BETSI regression diagnostics for photonic crystals made of TAPB-BTCA-COF particles (220 ± 4 nm) (S_{BET} = 584 m^2 g^{-1}).

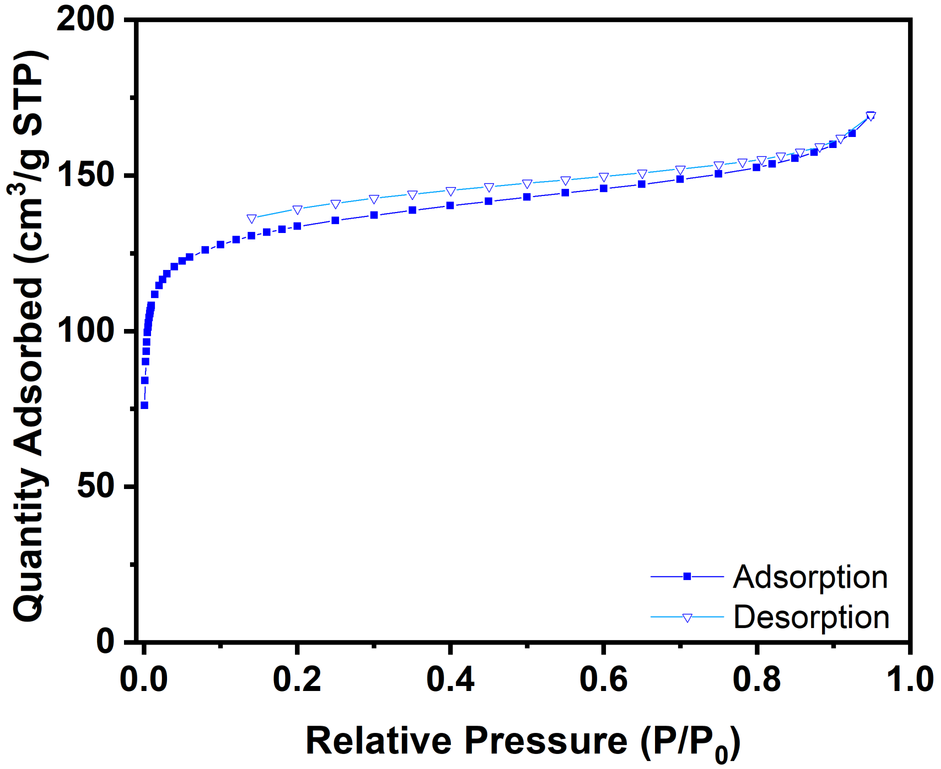

Figure S45. Nitrogen adsorption-desorption isotherm of photonic crystals made of TAPB-BTCA-COF particles (277 ± 5 nm) at 77 K.

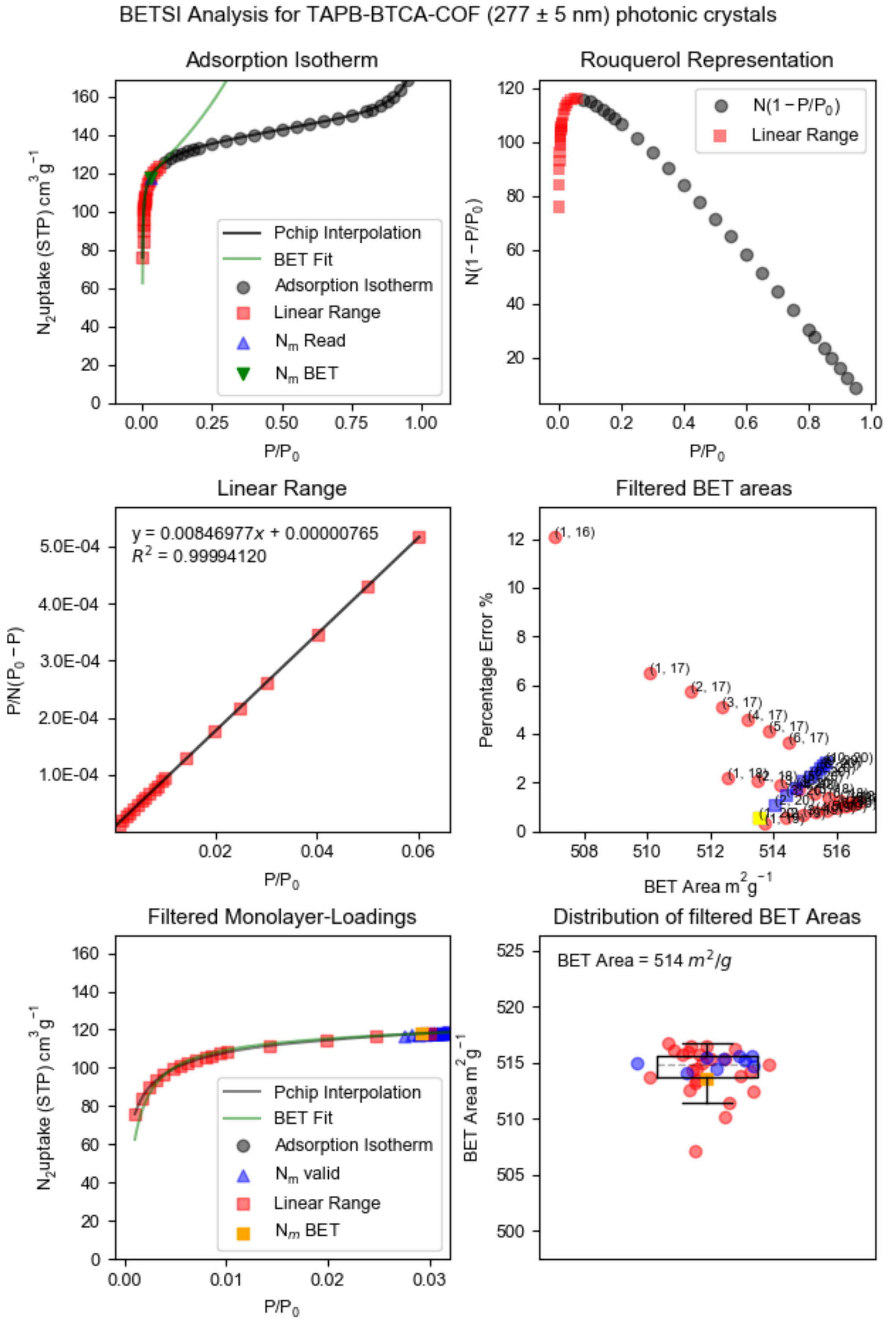

Figure S46. BETSI analysis of photonic crystals made of TAPB-BTCA-COF particles (277 ± 5 nm) (S_{BET} = 514 m^2 g^{-1}).

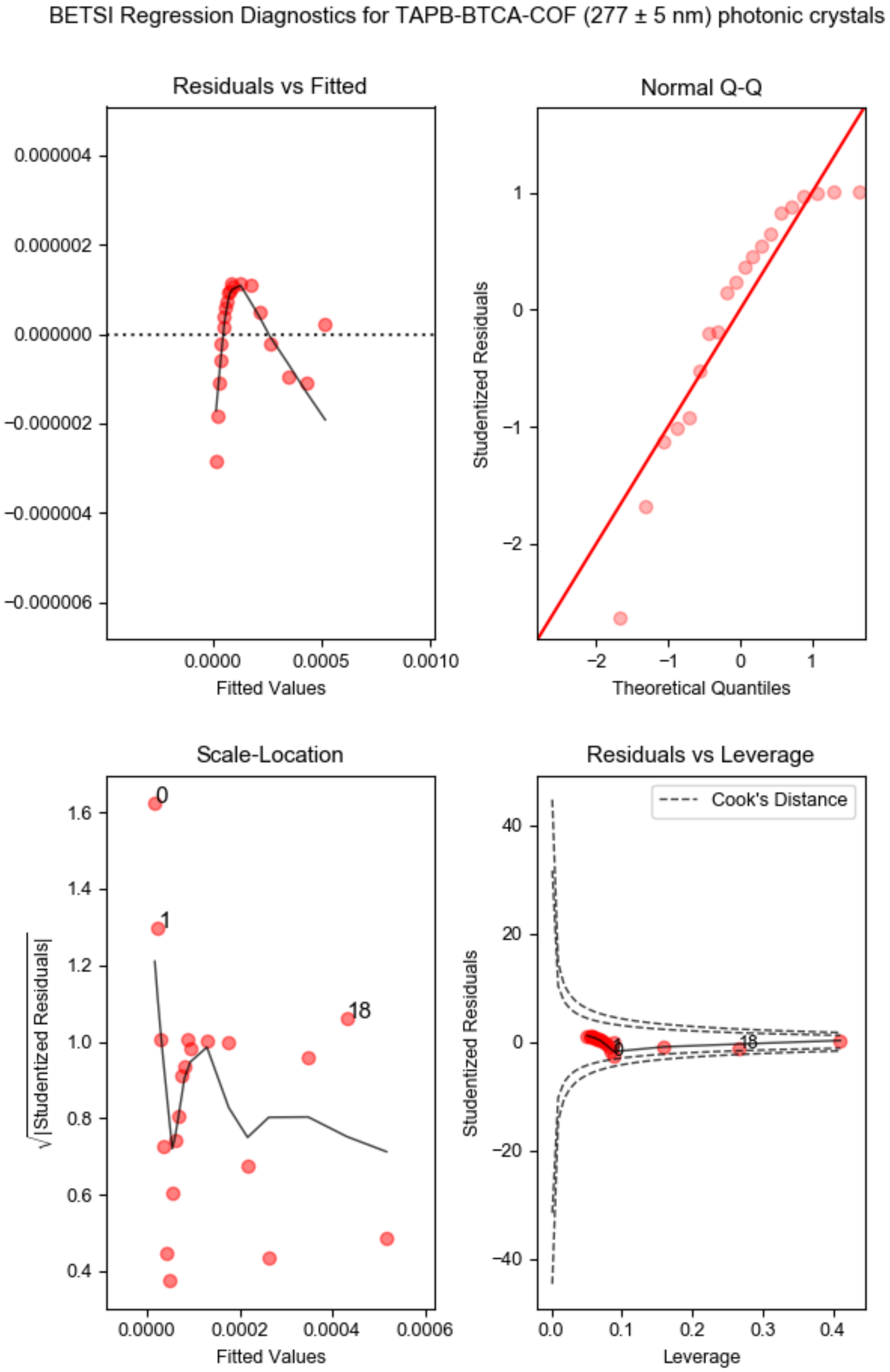

Figure S47. BETSI regression diagnostics for photonic crystals made of TAPB-BTCA-COF particles (277 ± 5 nm) (S_{BET} = 514 m^2 g^{-1}).

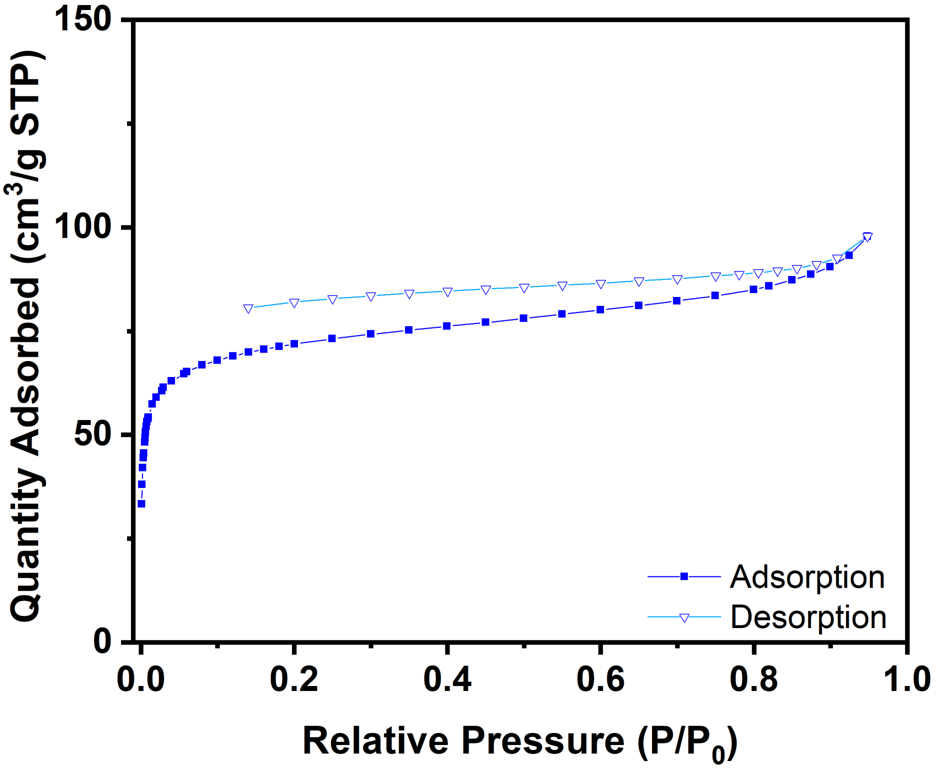

Figure S48. Nitrogen adsorption-desorption isotherm of photonic crystals made of TAPB-BTCA-COF particles (416 ± 7 nm) at 77 K.

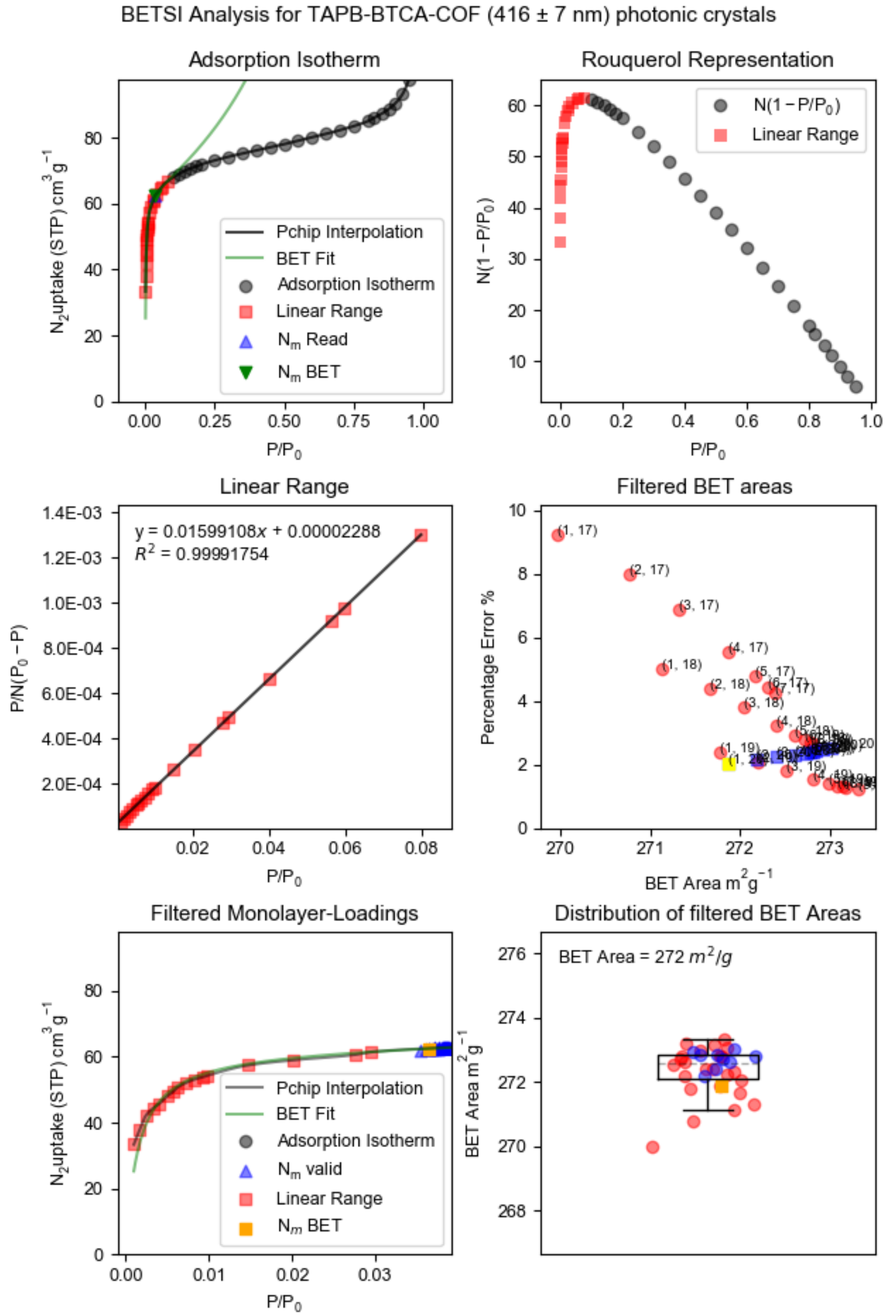

Figure S49. BETSI analysis of photonic crystals made of TAPB-BTCA-COF particles (416 ± 7 nm) (S_{BET} = 272 m^2 g^{-1}).

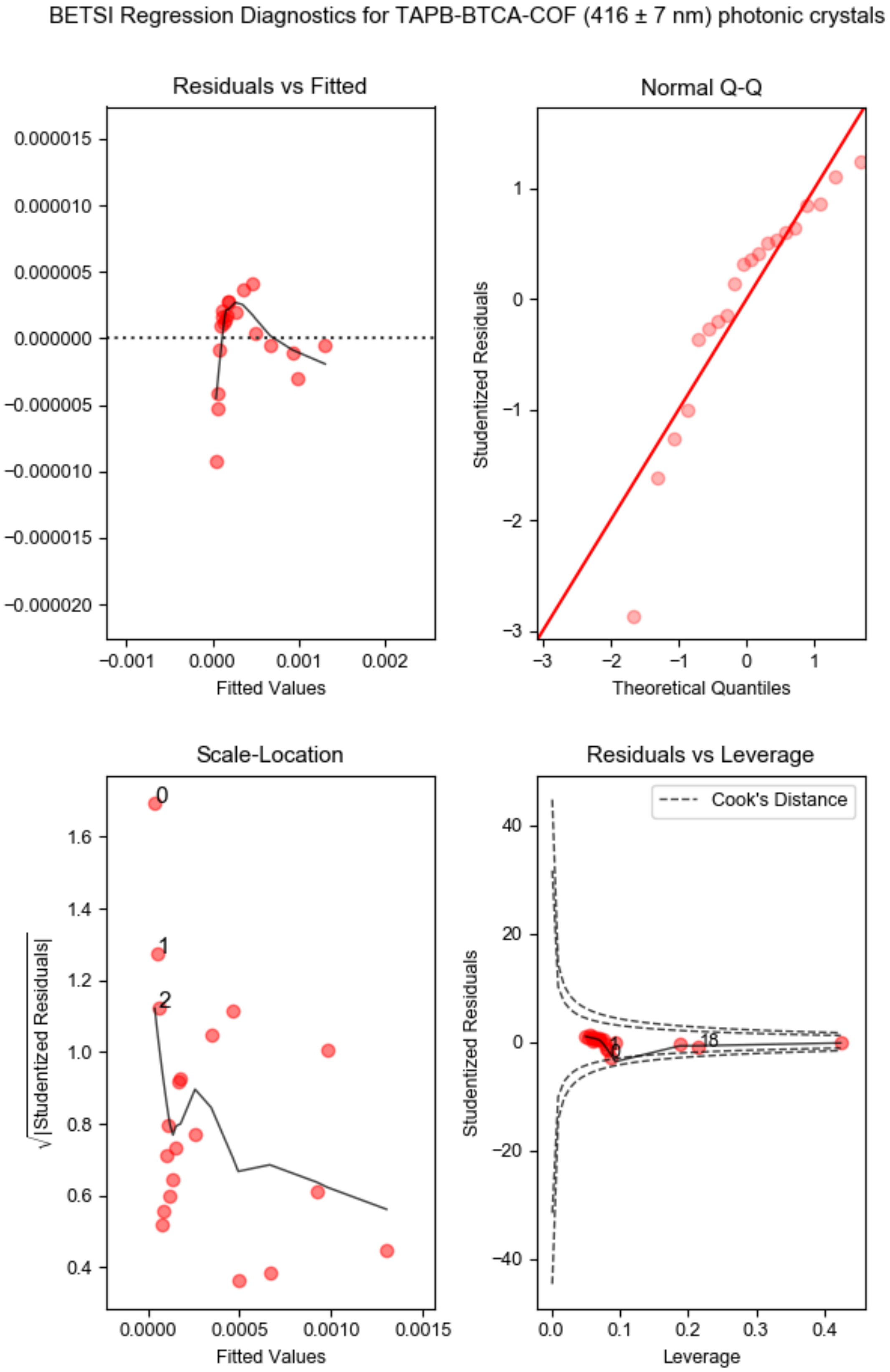

Figure S50. BETSI regression diagnostics for photonic crystals made of TAPB-BTCA-COF particles (416 ± 7 nm) (S_{BET} = 272 m^2 g^{-1}).

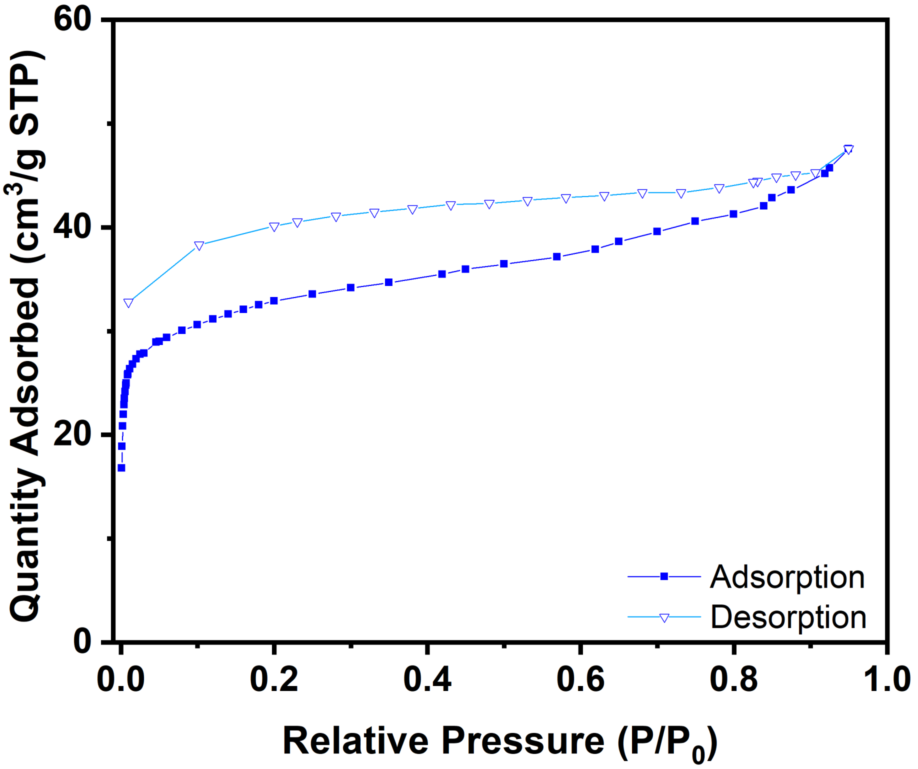

Figure S51. Nitrogen adsorption-desorption isotherm of photonic crystals made of TAPB-BTCA-COF particles (785 ± 12 nm) at 77 K.

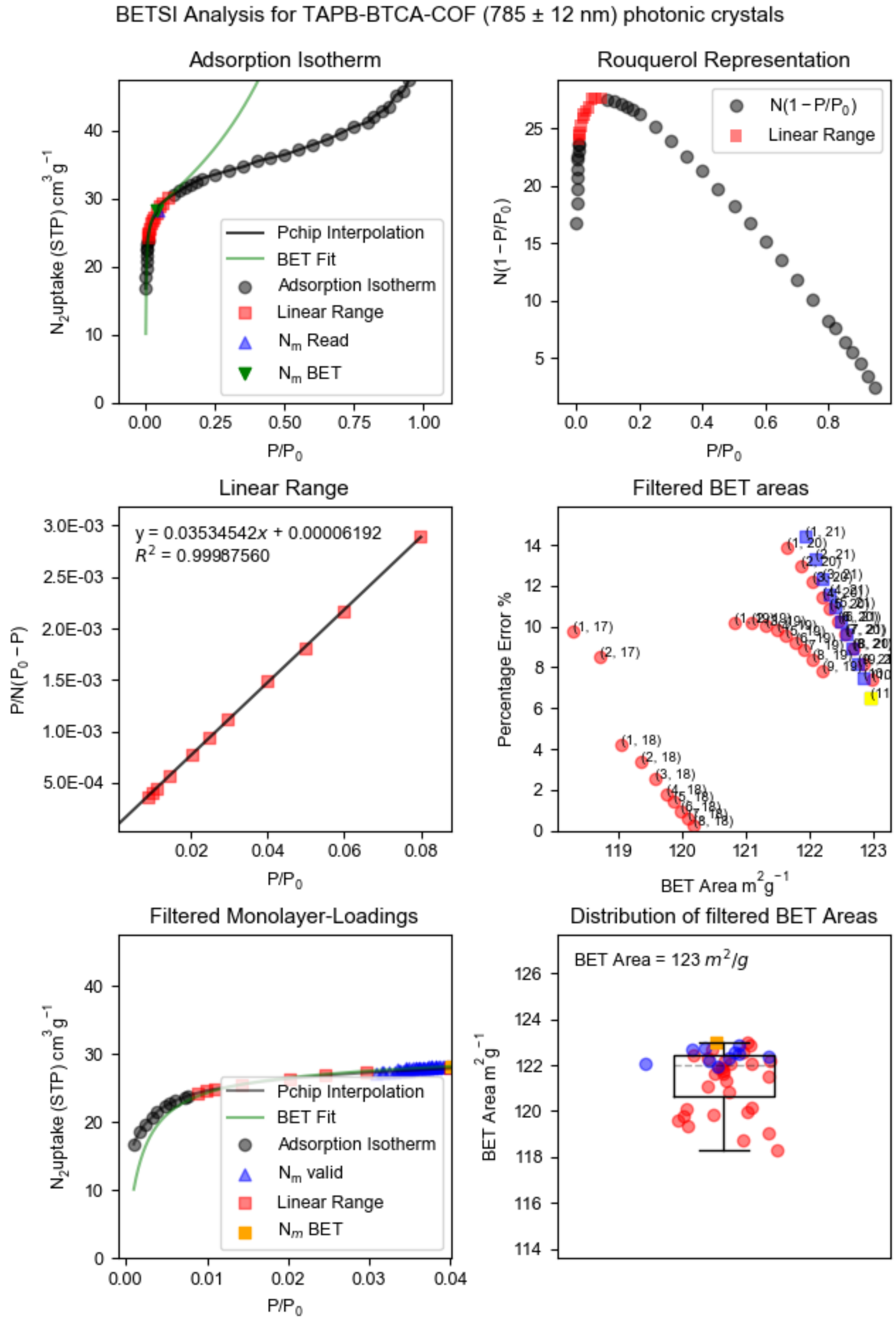

Figure S52. BETSI analysis of photonic crystals made of TAPB-BTCA-COF particles (785 ± 12 nm) (S_{BET} = 123 m^2 g^{-1}).

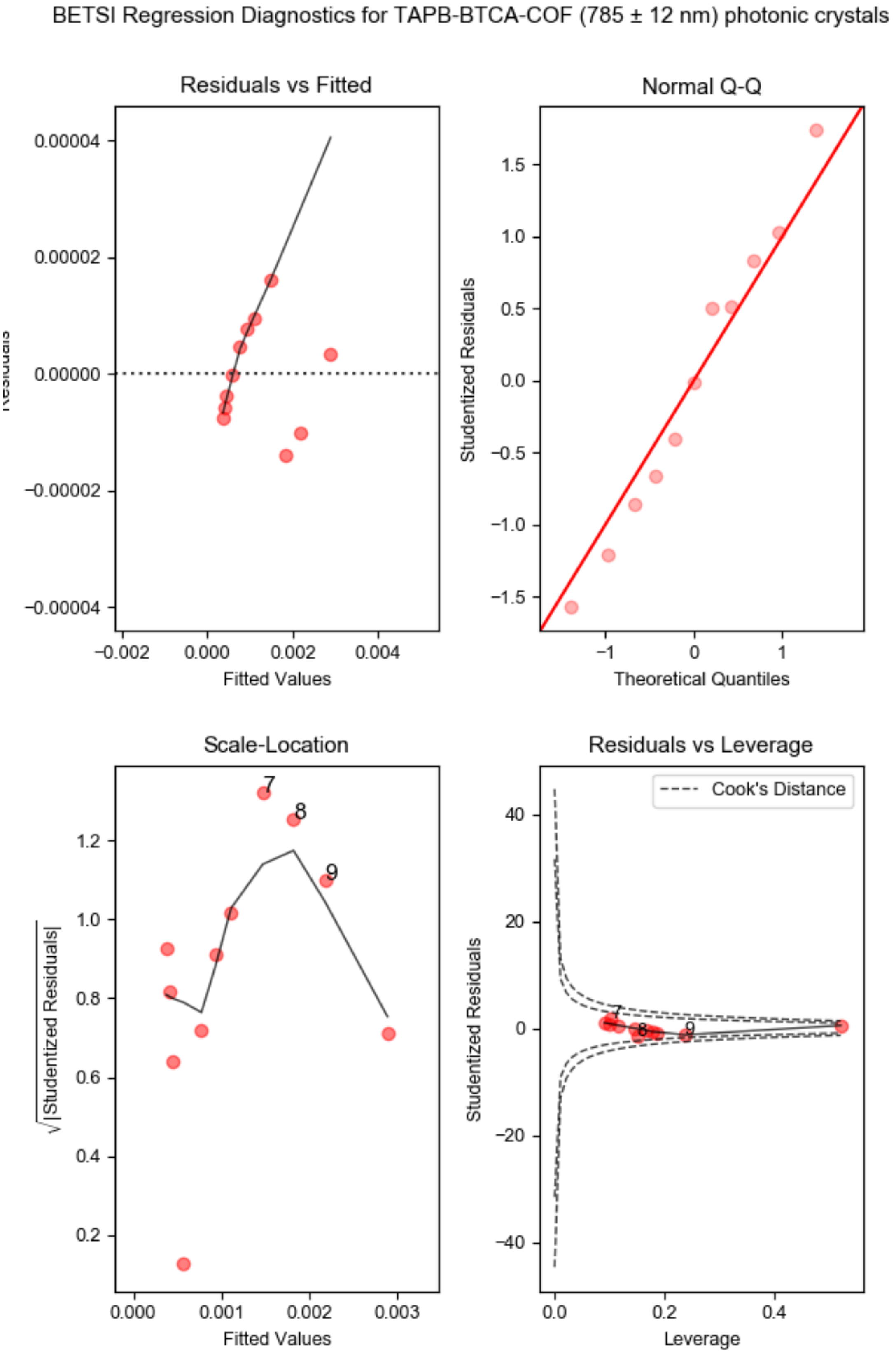

Figure S53. BETSI regression diagnostics for photonic crystals made of TAPB-BTCA-COF particles (785 ± 12 nm) (S_{BET} = 123 m^2 g^{-1}).

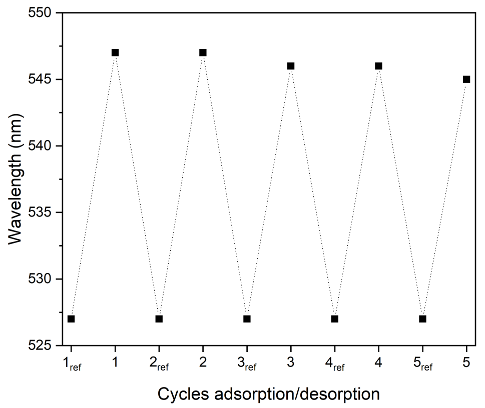

Figure S54. Bragg reflection maxima at normal incidence (θ=0°) for the PhCs comprising TAPB-BTCA-COF particles (203 ± 3 nm) before and after exposure to ethanol as a function of the number of adsorption-desorption cycles. The optical reflectance of the PhCs was measured after 30 min of exposure to the vapours of ethanol. At the end of each cycle and before the next one, the PhCs were activated by desorbing the ethanol at 120 °C for 30 min. No significant decrease of the Bragg reflection maxima was found throughout the ethanol adsorption-desorption cycles, indicating the high stability of theses PhCs.

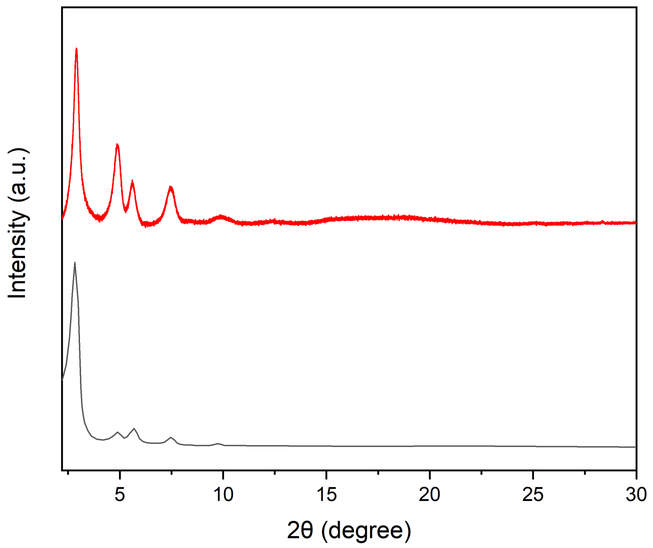

Figure S55. PXRD pattern of the urchin-like shaped TAPB-TP-COF particles. Black, reference; and red, urchin-like shaped TAPB-TP-COF particles.

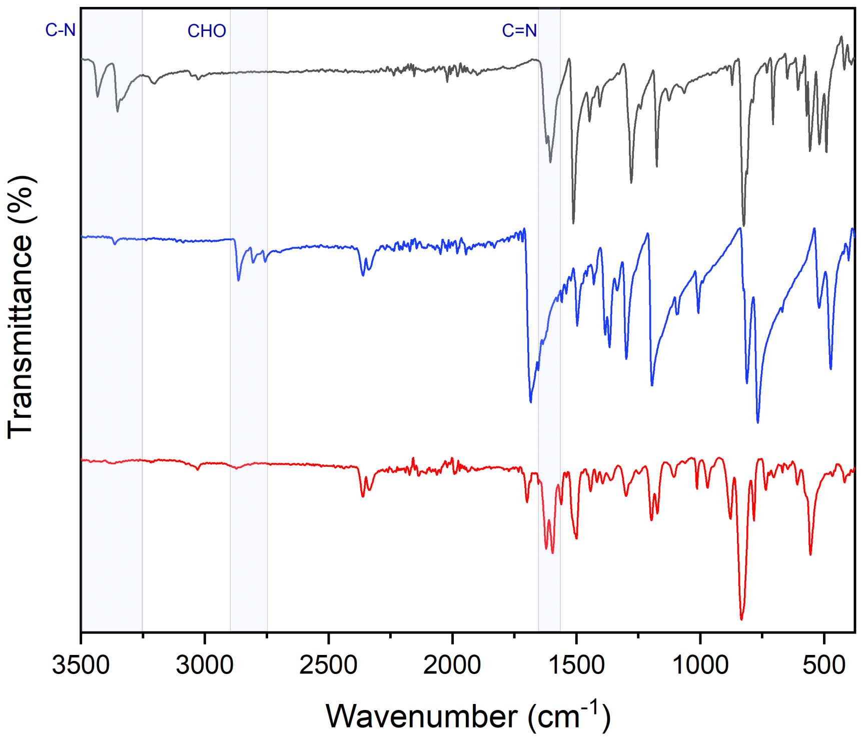

Figure S56. FT-IR spectra of urchin-like shaped TAPB-TP-COF particles (red), and TP (blue) and TAPB (black) linkers.

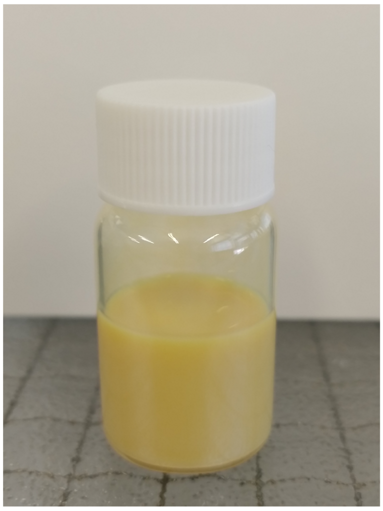
Figure S57. Photograph of a colloidal solution of TAPB-TP-COF particles.

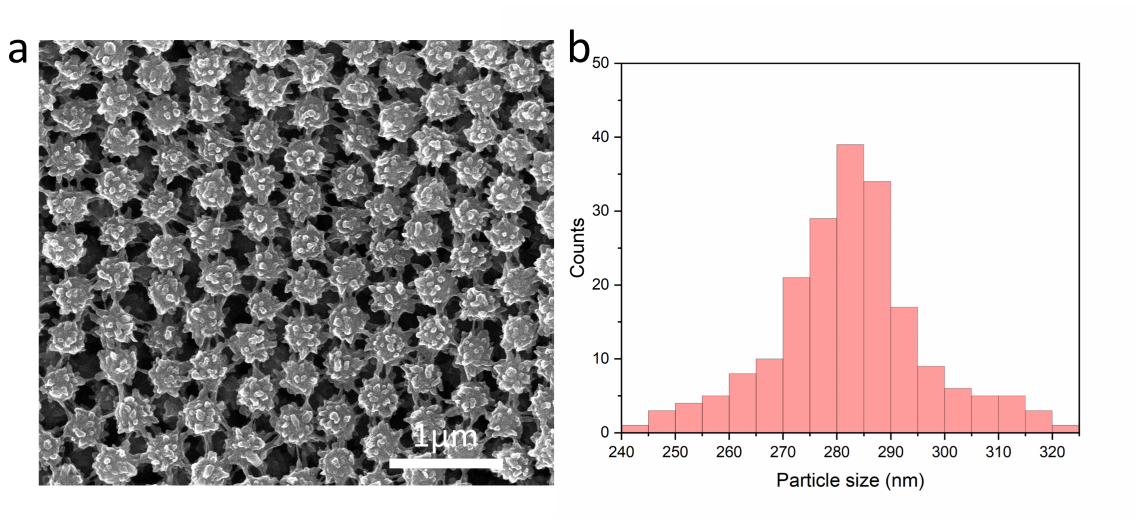

Figure S58. (a) Representative FE-SEM image of a self-assembled superstructure made of TAPB-TP-COF particles. (b) Size-distribution histogram of urchin-like shaped TAPB-TP-COF particles. Mean size = 282 nm. Standard deviation = 14.

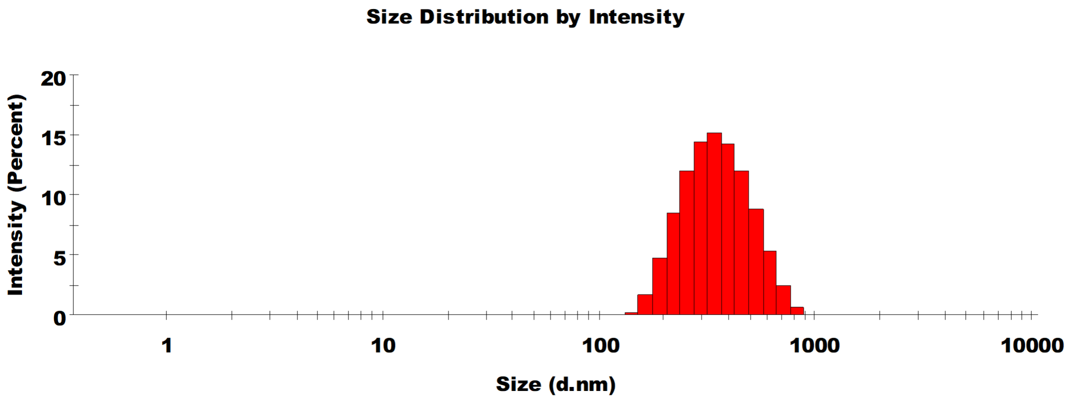

Figure S59. Dynamic light scattering histogram of the size distribution of urchin-like shaped TAPB-TP-COF particles. Mean average size: 327.5 nm (PdI: 0.104) (measured from FE-SEM images: 282 ± 14 nm).

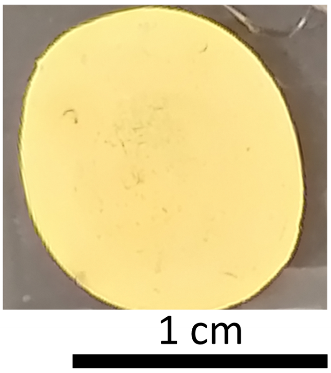

Figure S60. Photograph of the self-assembled PhC made of TAPB-TP-COF particles. The color of the COF-based PhC is strongly influenced by the yellow color of the COF particles.

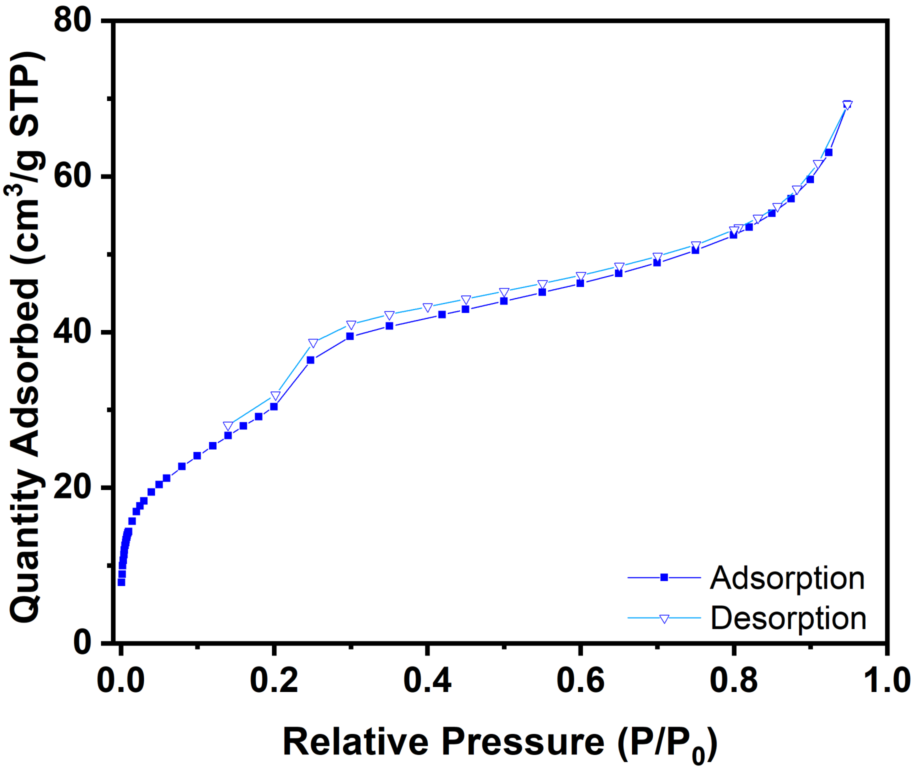

Figure S61. Nitrogen adsorption-desorption isotherm of TAPB-TP-COF particles (282 ± 14 nm) at 77 K.

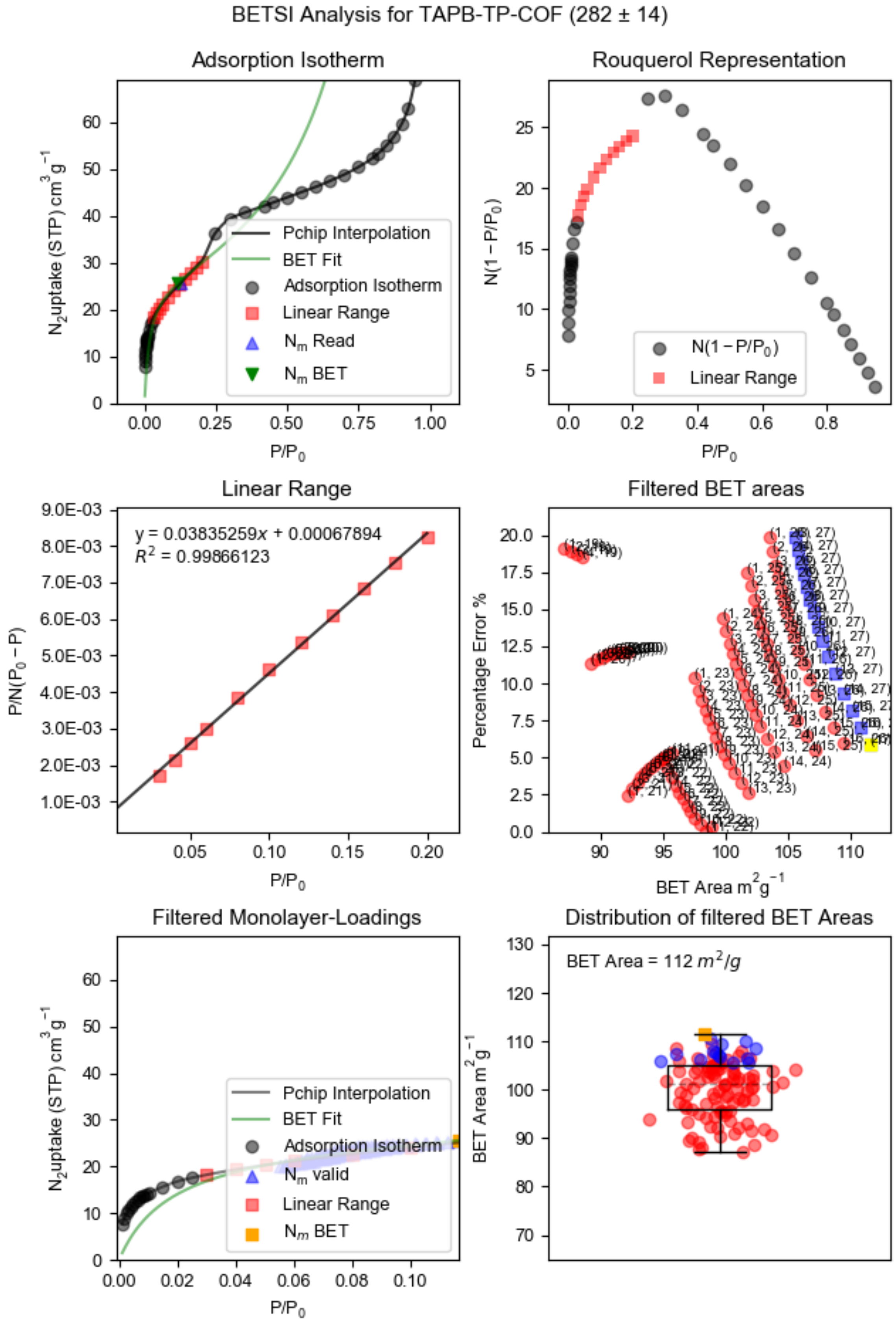

Figure S62. BETSI analysis of TAPB-TP-COF (282 ± 14 nm) (S_{BET} = 112 m² g⁻¹).

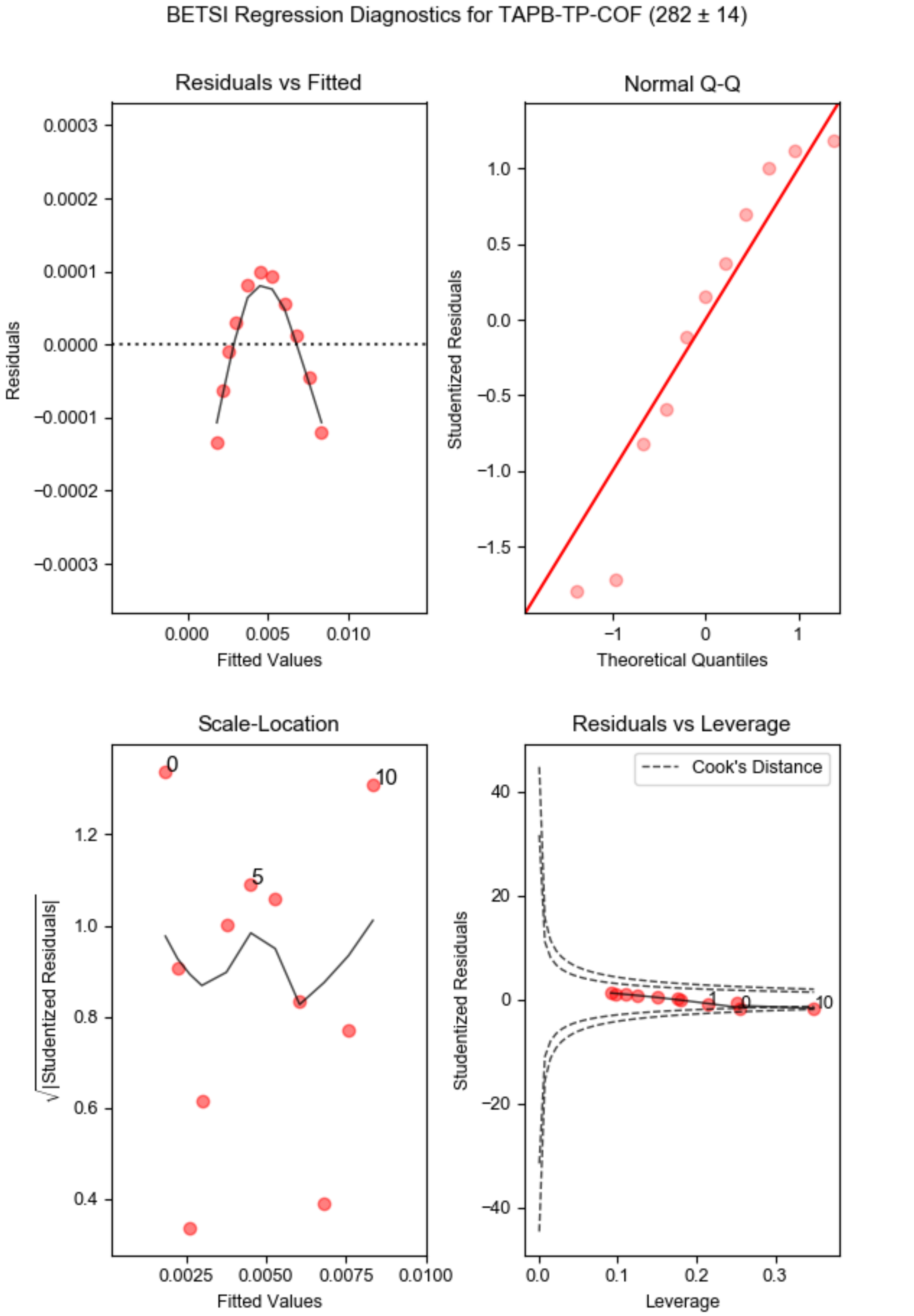

Figure S63. BETSI regression diagnostics for TAPB-TP-COF (282 ± 14 nm) (S_{BET} = 112 m^2 g^{-1}).

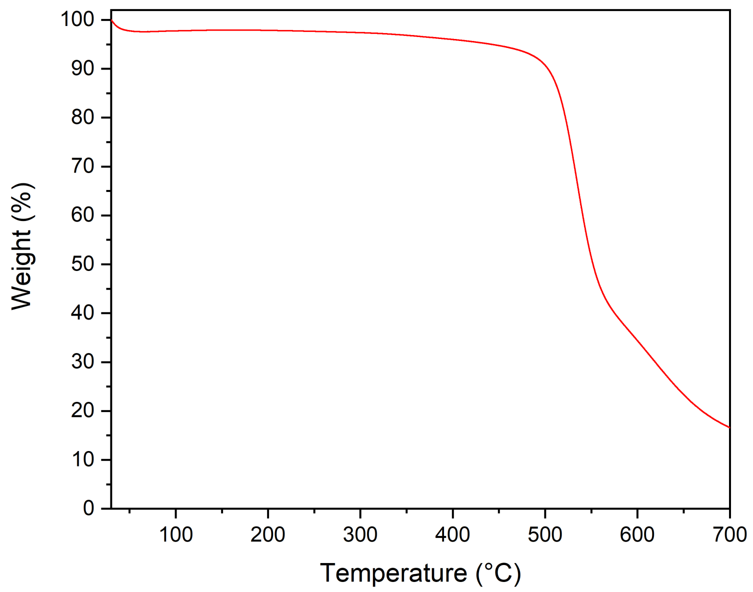

Figure S64. TGA analysis of PhCs made of TAPB-TP-COF particles. The weight loss around 500 °C is attributed to the decomposition of the COF particles.

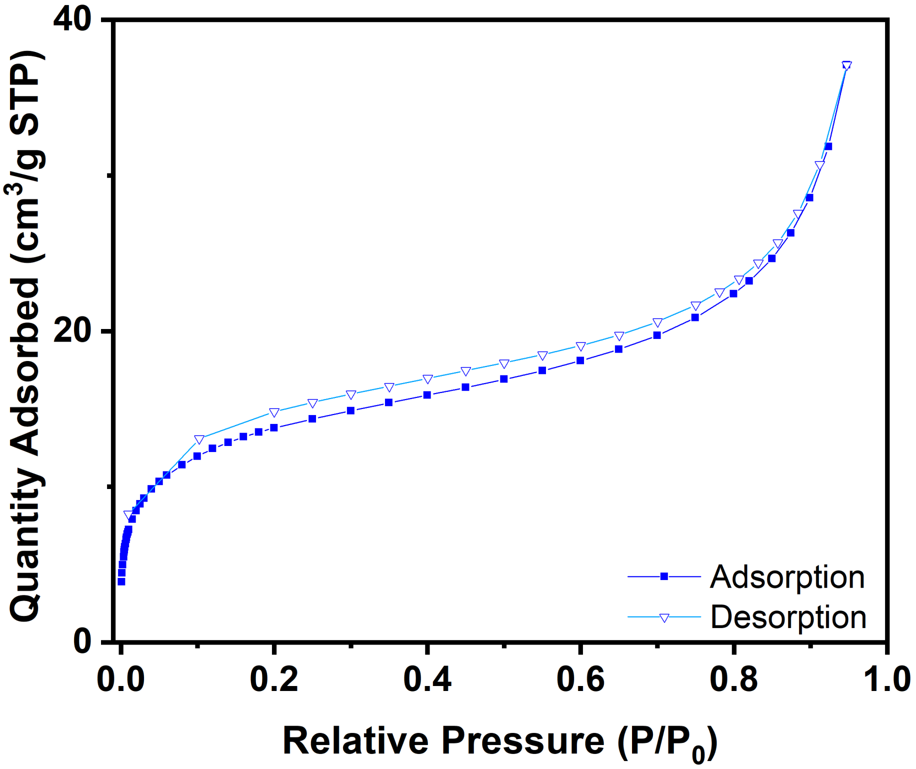

Figure S65. Nitrogen adsorption-desorption isotherm of photonic crystals made of TAPB-TP-COF particles (282 ± 14 nm) at 77 K.

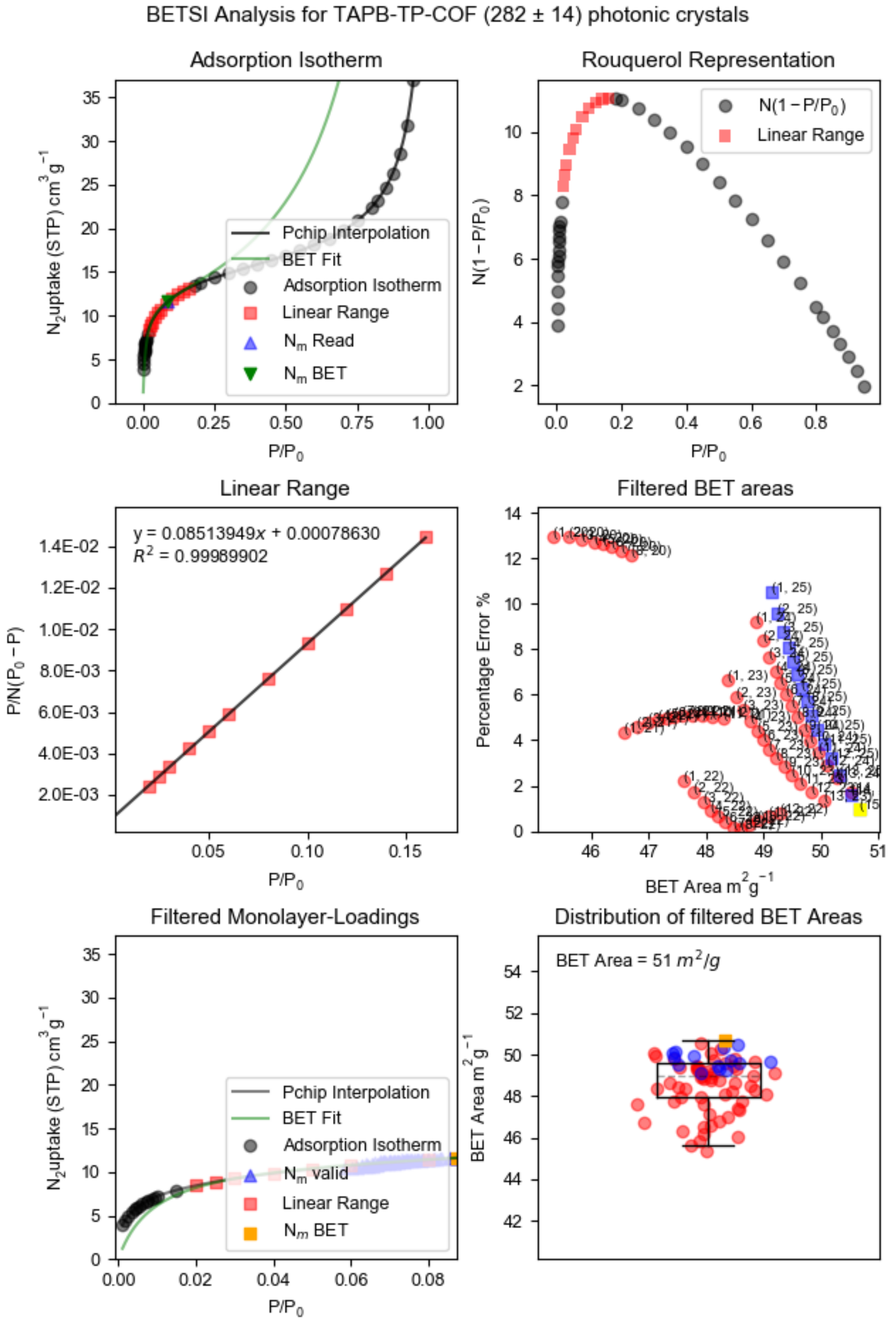

Figure S66. BETSI analysis of photonic crystals made of TAPB-TP-COF particles (282 ± 14 nm) (S_{BET} = 51 m² g⁻¹).

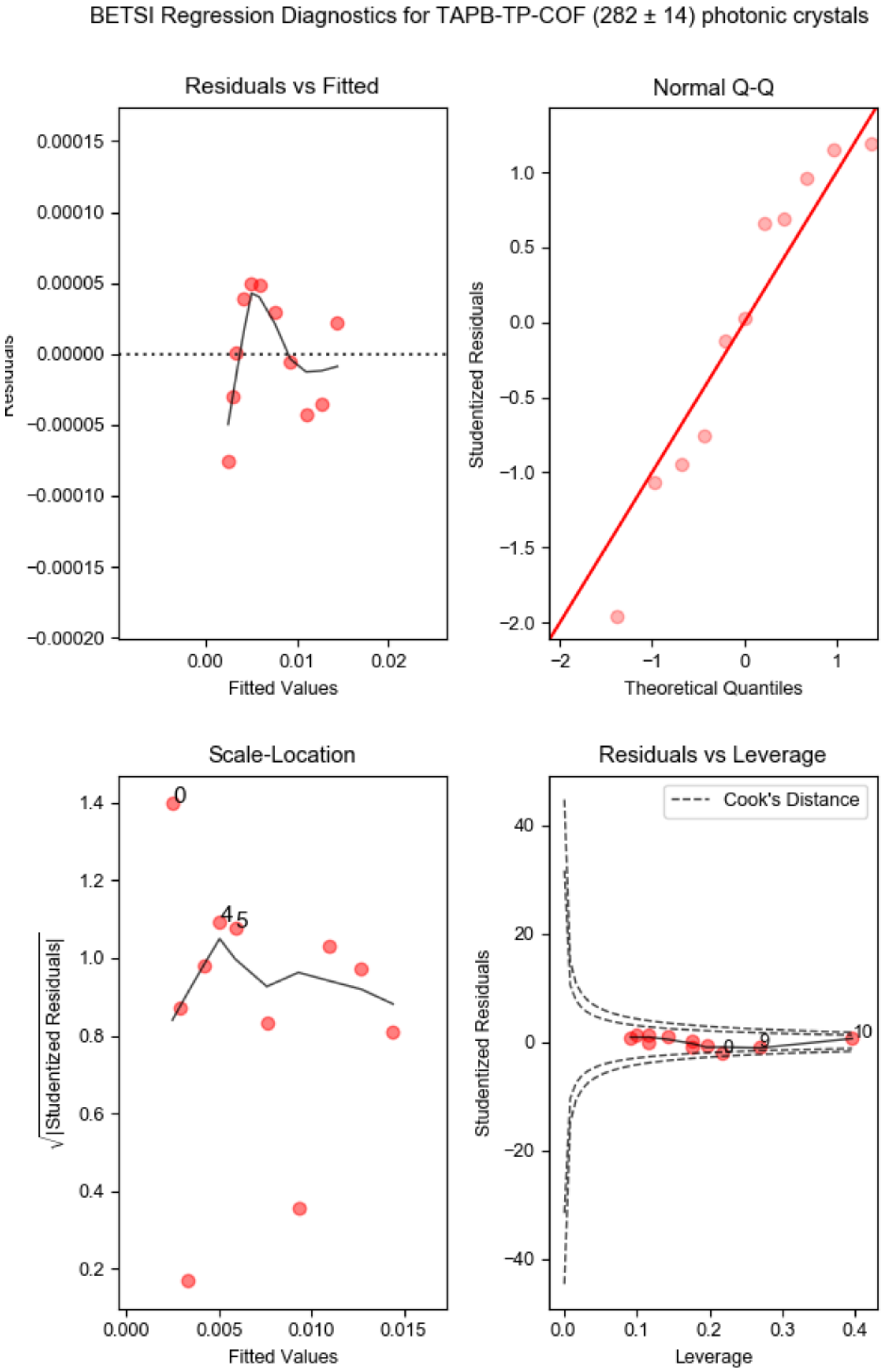

Figure S67. BETSI regression diagnostics for photonic crystals made of TAPB-TP-COF particles (282 ± 14 nm) (S_{BET} = 51 m^2 g^{-1}).

4. Supplementary tables

Supplementary Table 1. Diameter of spherical TAPB-BTCA-COF particles. BET surface area of TAPB-BTCA-COF particles and TAPB-BTCA-COF-based photonic crystals.

Particle size D (nm)*	BET surface area of COF particles (m^2 g^{-1})†	BET surface area of photonic crystals (m^2 g^{-1})†
179 ± 6 nm	1238	990
203 ± 3 nm	1131	761
220 ± 4 nm	964	584
277 ± 5 nm	534	514
416 ± 7 nm	399	272
785 ± 12 nm	223	123

* Measured from FE-SEM images.

† Determined from the nitrogen adsorption-desorption isotherms.

Supplementary Table 2. Diameter of spherical TAPB-BTCA-COF particles. Interplanar distance and optical bandgap of TAPB-BTCA-COF-based photonic crystals.

Particle size D (nm)*	Interplanar distance d_{111} (nm)†	Bandgap λ_c (nm)‡	Effective refractive index of photonic crystals formed by self-assembly of TAPB-BTCA-COF particles§	Refractive index of TAPB-BTCA-COF particlesж
179 ± 6 nm	146 ± 5	475	1.63 ± 0.07	1.80 ± 0.09
203 ± 3 nm	166 ± 2	515	1.55 ± 0.03	1.70 ± 0.04
220 ± 4 nm	180 ± 3	530	1.48 ± 0.04	1.61 ± 0.04
277 ± 5 nm	226 ± 4	634	1.40 ± 0.03	1.52 ± 0.04
416 ± 7 nm	339 ± 6	966	1.42 ± 0.03	1.55 ± 0.04
785 ± 12 nm	640 ± 10	---	---	---

* Measured from FE-SEM images.

† Calculated from $d_{111} = 0.816 \cdot D$, where D is the center-to-center distance of the COF particles.

‡ Measured from UV-vis reflectance spectra.

§ Determined using the Bragg-Snell law ($\lambda_c = 2 \cdot n_{eff} \cdot d_{111}$).

ж Calculated from $n_c = \sqrt{\frac{n_{eff}^2 - n_m^2 \cdot V_m}{V_c}}$, where n_{eff}, n_c and n_m are the effective refractive index, the refractive index of TAPB-BTCA-COF particles and the refractive index of the surrounding air (1.00).[3] V_c and V_m are the volume fractions of TAPB-BTCA-COF particles and air, respectively. For (111) planes in the fcc lattice, $V_c = 0.74$ and $V_m = 0.26$.

Supplementary Table 3. Bandgaps and calculated refractive indices of the self-assembled photonic crystals (particle size: 277 ± 5 nm) exposure to different alcohols.

Guest molecules	Bandgap (nm)†	Refractive index of guest molecule n_{guest}	Experimental effective refractive index of photonic crystals formed by self-assembly of TAPB-BTCA-COF particles§
Air	628	1.00[3]	1.39 ± 0.03
MeOH	651	1.33[4]	1.44 ± 0.03
EtOH	654	1.36[4]	1.45 ± 0.03
Isopropanol	657	1.38[4]	1.45 ± 0.03
1-butanol	658	1.40[4]	1.46 ± 0.03

† Measured from UV-vis reflectance spectra.
§ Determined from the Bragg-Snell law, $n_{eff} = \lambda_c / (2 \cdot d_{111})$. Here, λ_c is the measured bandgap, and d_{111} is 226 ± 4 nm.

Supplementary Table 4. Diameter of spherical TAPB-TP-COF particles. BET surface area of TAPB-TP-COF particles and TAPB-TP-COF-based photonic crystals.

Particle size D (nm)*	BET surface area of TAPB-TP-COF particles (m^2 g^{-1})†	BET surface area of TAPB-TP-COF-based photonic crystals (m^2 g^{-1})†
282 ± 14 nm	112	51

* Measured from FE-SEM images.

† Determined from the nitrogen adsorption-desorption isotherms.

5. References

(1) Osterrieth, J. W. M.; *et al*. How Reproducible are Surface Areas Calculated from the BET Equation? *Adv. Mater.* **2022**, 34, 2201502.
(2) Ma, W.; Zheng, Q.; He, Y.; Li, G.; Guo, W.; Lin, Z.; Zhang, L. Size-Controllable Synthesis of Uniform Spherical Covalent Organic Frameworks at Room Temperature for Highly Efficient and Selective Enrichment of Hydrophobic Peptides. *J. Am. Chem. Soc.* **2019**, 141, 18271-18277.
(3) Ciddor, P. E. Refractive index of air: new equations for the visible and near infrared. *Appl. Opt.* **1996**, 35, 1566-1573.
(4) O'Brien, R. N.; Quon, D. Refractive index of some alcohols and saturated hydrocarbons at 6328 A. *J. Chem. Eng. Data* **1968**, 13, 517.
